# Design, synthesis, and antiproliferative activity of new 1,2,3-triazole/quinazoline-4-one hybrids as dual EGFR/BRAF^V600E^ inhibitors†

**DOI:** 10.1039/d4ra06694d

**Published:** 2024-12-05

**Authors:** Amira M. Mohamed, Ola M. F. Abou-Ghadir, Yaser A. Mostafa, Zainab M. Almarhoon, Stefan Bräse, Bahaa G. M. Youssif

**Affiliations:** a Pharmaceutical Organic Chemistry Department, Faculty of Pharmacy, Assiut University Assiut 71526 Egypt bgyoussif2@gmail.com bahaa.youssif@pharm.aun.edu.eg; b Department of Chemistry, College of Science, King Saud University Riyadh 11451 Saudi Arabia; c Institute of Biological and Chemical Systems, IBCS-FMS, Karlsruhe Institute of Technology 76131 Karlsruhe Germany braese@kit.edu

## Abstract

A novel series of 1,2,3-triazole/quinazoline-4-one hybrids (8a–t) were designed and synthesized as dual-targeted antiproliferative agents. Compounds 8a–t were evaluated for their antiproliferative efficacy against a panel of four cancer cell lines. The results indicated that most of the evaluated compounds exhibited strong antiproliferative activity, with 8f, 8g, 8h, 8j, and 8l demonstrating the highest potency. These five compounds were investigated as EGFR and BRAF^V600E^ inhibitors. The *in vitro* tests showed that compounds 8g, 8h, and 8j are strong antiproliferative agents that might work as dual EGFR/BRAF^V600E^ inhibitors. Compounds 8g and 8h were further examined as activators of caspases 3, 8, and Bax and down-regulators of the anti-apoptotic protein Bcl2. The results indicated that the studied compounds had considerable apoptotic antiproliferative action. The investigation of the cell cycle and apoptosis revealed that compound 8g induces cell cycle arrest during the G1 phase transition. Molecular docking experiments are thoroughly examined to validate the binding interactions of the most active hybrids with the active sites of EGFR and BRAF^V600E^. The data indicated that the examined compounds can efficiently engage with essential amino acid residues in both kinases.

## Abstract 2

A series of new quinazoline/1,2,3-triazole hybrids were designed and synthesized. The new compounds were evaluated as antiproliferative agents targeting EGFR, and BRAF^V600E^.

## Graphical abstract

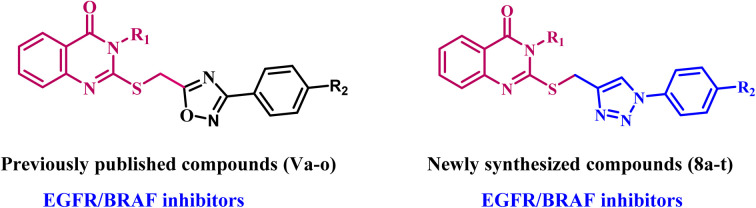

## 1. Introduction

Cancer is abnormal cellular proliferation resulting from an imbalance between cell division and apoptosis.^1,2^ Despite the availability of different therapeutic modalities, including surgical intervention, radiation, immunotherapy, and chemotherapy, cancer continues to be a contentious clinical issue. Nevertheless, effective chemotherapeutic agents with minimal side effects have garnered significant interest.^3,4^ Elucidating and revealing cancer's molecular and cellular roots has significantly contributed to advancing cancer therapies. Currently, cancer therapy identifies membrane receptors of the tyrosine kinase (TK) family as primary targets.^5,6^ The epidermal growth factor (EGF) family includes important membrane receptors, with the epidermal growth factor receptor (EGFR) getting much attention. It has been found that blocking EGFR signaling is recognized as a potent therapeutic strategy.^7,8^

**Fig. 1:**
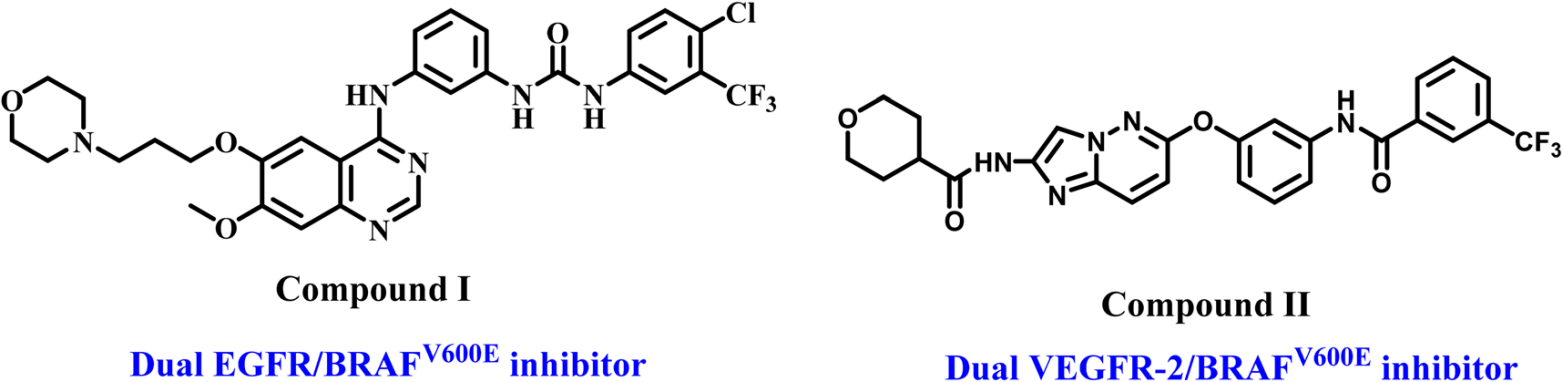
Structures of dual inhibitors BRAF/TKs compounds I and II.

Clinical studies have shown that combining B-Rapidly Accelerated Fibrosarcoma (B-raf proto-oncogene or BRAF) and tyrosine kinase (TK) inhibitors effectively stops tumor growth and overcomes resistance. Concomitant use of EGFR inhibitors may mitigate resistance to vemurafenib, a mutant BRAF (BRAF^V600E^) inhibitor, in thyroid cancer.^9^ This combination has also demonstrated encouraging outcomes in BRAF^V600E^ colorectal cancer.^10^ Furthermore, researchers have found several compounds *in vitro* that contain the critical pharmacophoric groups required to inhibit tyrosine kinases, such as epidermal growth factor receptor/vascular endothelial growth factor receptor-2 (EGFR/VEGFR-2) and BRAF.^11,12^ For example, compound I (Fig. 1) inhibited wild-type BRAF and EGFR, exhibiting IC_50_ values in the nanomolar range. Furthermore, imidazo[1,2-*b*]pyridazine II inhibited BRAF and VEGFR-2.

**Fig. 2:**
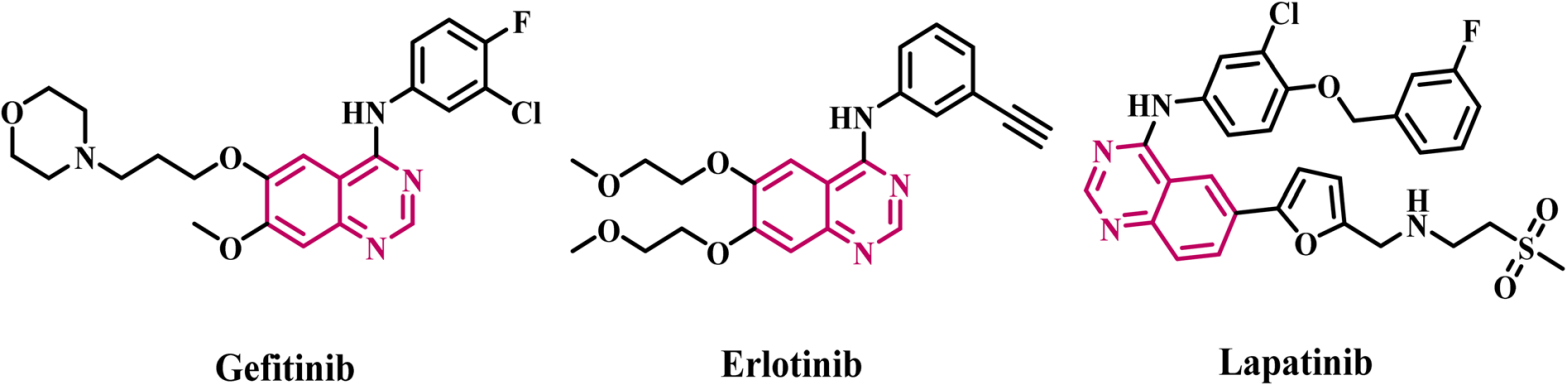
Structures of FDA-approved quinazoline-based anticancer agents.

Heterocyclic compounds have yielded a plethora of commercialized medicines and bioactive substances, and their notable cytotoxicity has been essential in the design and production of anticancer agents.^13,14^ Consequently, quinazolines have garnered significant attention owing to their efficacy and specificity.^15,16^ Quinazolines' anticancer properties can be traced back to Paganini (vaccine), a naturally occurring quinazoline discovered in 1888.^17^ Gefitinib, erlotinib, and lapatinib are FDA-approved quinazoline-based anticancer agents that function as EGFR inhibitors^18–20^ (Fig. 2).

**Fig. 3:**
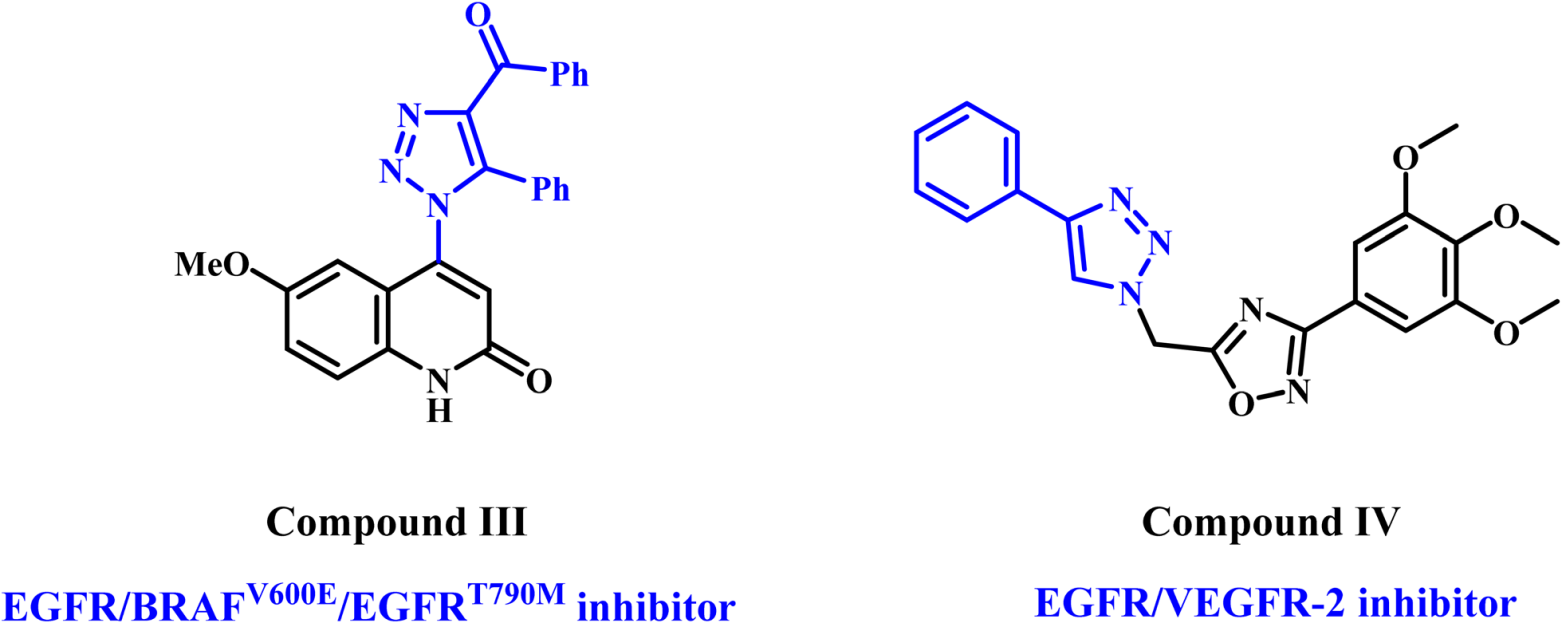
Structures of 1,2,3-triazole-based derivatives III and IV.

On the other hand, 1,2,3-triazoles are important scaffolds often synthesized using the 1,3-dipolar cycloaddition reaction (click chemistry reaction) between terminal acetylenes and azides.^21–23^ The anticancer activity of 1,2,3-triazoles has garnered significant attention regarding their therapeutic properties.^24–26^ In a recent publication from our lab,^24^ we describe the discovery of a novel class of 1,2,3-triazole/quinoline hybrids as antiproliferative compounds that act as multi-targeted inhibitors. Most novel compounds demonstrated considerable antiproliferative activity against a panel of four cancer cell lines. With IC_50_ values of 57 nM for EGFR, 68 nM for BRAF^V600E^, and 9.70 nM for EGFR^T790M^, compound III (Fig. 3) was the most effective at blocking these three proteins. The apoptotic assay results indicated that compound III functions as caspase-3, 8, and Bax activator while down-regulating the antiapoptotic protein Bcl2.

In another publication,^26^ we report compound IV (Fig. 3), a 1,2,3-triazole/1,2,4-oxadiazole hybrid, as a potent apoptotic antiproliferative agent that may function as dual inhibitors of EGFR and VEGFR-2.

### 1.1. Rational design

**Fig. 4:**
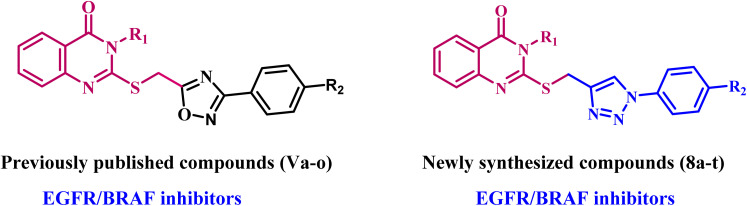
Structures of previously published quinazoline-4-one derivatives Va–o and new target compounds 8a–t.

Recently,^27^ we reported a series of 1,2,4-oxadiazole/quinazoline-4-one hybrids (Va–o, Fig. 4) designed as antiproliferative agents targeting EGFR/BRAF^V600E^. The results indicated that most evaluated compounds exhibited strong antiproliferative activity, with Vb, Vc, Vh, Vk, and Vl demonstrating the highest potency. We tested these compounds as EGFR and BRAF^V600E^ inhibitors and discovered they are good at stopping cell growth and may work as dual inhibitors of EGFR and BRAF^V600E^.

As recently discovered, hybrid molecules have addressed several challenges associated with traditional drugs, including side effects and multidrug resistance (MDR).^28^ In support of our efforts to develop anticancer drugs with dual or multi-targeted mechanisms,^27,29–35^ we synthesized new quinazolin-4-ones coupled to 1,2,3-triazoles (8a–t, Fig. 4). We assessed their antiproliferative effectiveness against four different cancer cells. The most potent compounds were further tested as dual inhibitors of EGFR/BRAF. Also, the most potent hybrids were investigated for their apoptotic potential as activators of caspase 3,8 and BAX and down-regulators of antiapoptotic Bcl2. Finally, the most potent derivative was tested for cell cycle arrest and apoptosis.

## 2. Experimental

### 2.1. Chemistry

General details: see Appendix A (ESI file).†

#### 2.1.1. General procedures for the synthesis of the target compounds (8a–t)

To a mixture of (2-propynyl)thioquinzolin-4(3*H*)-ones (0.85 mmol, 1 eq.) and *p*-azido phenyl derivatives (1.02 mmol, 1.2 eq.) in 20 mL of THF, an aqueous solution (5 mL) of sodium ascorbate (0.03 g, 0.17 mmol, 0.2 eq.) and CuSO_4_·5H_2_O (0.01 g, 0.0425 mmol, 0.05 eq.) was added. The mixture was refluxed for 24–48 h. After completion of reaction, the solvent was evaporated under vacuum, the precipitate was filtered, washed with cold water, dried and recrystallized by methanol to obtain 8a–t compounds.

##### 2.1.1.1. 2-(((1-Phenyl-1*H*-1,2,3-triazol-4-yl)methyl)thio)-3-phenyl quinazolin-4(3*H*)-one (8a)

Yield: 0.26 g (74%), white solid, m.p: 136–138 °C, *R*_f_. 0.56 (hexane : ethyl acetate, 1 : 2, v/v). ^1^H NMR (400 MHz, *δ* ppm CDCl_3_): 8.19 (d, *J* = 7.8 Hz, 1H, Ar–H), 8.01 (s, 1H, triazole CH), 7.70 (t, *J* = 7.4 Hz, 1H, Ar–H), 7.61 (d, *J* = 7.4 Hz, 3H, Ar–H), 7.47–7.37 (m, 5H, Ar–H), 7.36–7.30 (m, 2H, Ar–H), 7.24–7.14 (m, 2H, Ar–H), 4.50 (s, 2H, S–CH_2_). ^13^C NMR (100 MHz, *δ* ppm CDCl_3_): 161.71, 156.87, 147.68, 135.52, 135.19, 134.89, 130.18, 129.80, 129.78, 129.44, 129.16, 128.81, 128.44, 127.50, 126.12, 126.01, 120.59, 120.01, 27.25. LC-MS (*m*/*z*) for: C_23_H_17_N_5_OS (exact mass = 411.12); calculated [M + H]^+^: 412.12; found [M + H]^+^: 412.00.

##### 2.1.1.2. 2-(((1-(4-Chlorophenyl)-1*H*-1,2,3-triazol-4-yl)methyl)thio)-3-phenyl quinazolin-4(3*H*)-one (8b)

Yield: 0.29 g (77%), white solid, m.p: 146–148 °C, *R*_f_. 0.55 (hexane : ethyl acetate, 1 : 2, v/v). ^1^H NMR (400 MHz, *δ* ppm DMSO-*d*_6_): 8.76 (s, 1H, triazole CH), 8.08 (d, *J* = 7.6 Hz, 1H, Ar–H), 7.87 (d, *J* = 8.8 Hz, 2H, Ar–H *p*-Cl C_6_H_4_), 7.84 (d, *J* = 7.8 Hz, 1H, Ar–H), 7.77 (d, *J* = 8.0 Hz, 1H, Ar–H), 7.63 (d, *J* = 8.8 Hz, 2H, Ar–H *p*-Cl C_6_H_4_), 7.59–7.52 (m, 3H, Ar–H), 7.51–7.43 (m, 3H, Ar–H), 4.53 (s, 2H, S–CH_2_). ^13^C NMR (100 MHz, *δ* ppm DMSO-*d*_6_): 160.86, 156.49, 147.32, 143.95, 135.77, 135.37, 135.05, 133.03, 130.06, 129.92, 129.61, 129.51, 126.64, 126.30, 126.18, 122.29, 121.83, 119.71, 26.66. LC-MS (*m*/*z*) for: C_23_H_16_ClN_5_OS (exact mass = 445.08); calculated [M + H]^+^: 446.08; found [M + H]^+^: 446.00.

##### 2.1.1.3. 2-(((1-(4-Methoxyphenyl)-1*H*-1,2,3-triazol-4-yl)methyl)thio)-3-phenylquinazolin-4(3*H*)-one (8c)

Yield: 0.29 g (78%), white solid, m.p: 152–154 °C, *R*_f_. 0.58 (hexane : ethyl acetate, 1 : 2, v/v). ^1^H NMR (400 MHz, *δ* ppm DMSO-*d*_6_): 8.63 (s, 1H, triazole CH), 8.08 (d, *J* = 7.6 Hz, 1H, Ar–H), 7.84 (t, *J* = 7.2 Hz, 1H, Ar–H), 7.76 (d, *J* = 8.0 Hz, 1H, Ar–H), 7.73 (d, *J* = 8.6 Hz, 2H, Ar–H *p*-O–CH_3_ C_6_H_4_), 7.63–7.40 (m, 6H, Ar–H), 7.10 (d, *J* = 8.6 Hz, 2H, Ar–H *p*-O–CH_3_ C_6_H_4_), 4.52 (s, 2H, S–CH_2_), 3.80 (s, 3H, O–CH_3_). ^13^C NMR (100 MHz, *δ* ppm DMSO-*d*_6_): 160.88, 159.34, 156.59, 147.34, 143.32, 135.80, 135.07, 130.06, 130.01, 129.61, 129.52, 126.66, 126.28, 126.18, 122.22, 121.83, 119.72, 114.94, 55.63, 26.77. LC-MS (*m*/*z*) for: C_24_H_19_N_5_O_2_S (exact mass = 441.13); calculated [M + H]^+^: 442.13; found [M + H]^+^: 442.10.

##### 2.1.1.4. 2-(((1-(*p*-Tolyl)-1*H*-1,2,3-triazol-4-yl)methyl)thio)-3-phenylquinazolin-4(3*H*)-one (8d)

Yield: 0.27 g (75%), white solid, m.p: 138–140 °C, *R*_f_. 0.57 (hexane : ethyl acetate, 1 : 2, v/v). ^1^H NMR (400 MHz, *δ* ppm DMSO-*d*_6_): 8.68 (s, 1H, triazole CH), 8.08 (d, *J* = 7.2 Hz, 1H, Ar–H), 7.83 (d, *J* = 8.4 Hz, 1H, Ar–H), 7.76 (d, *J* = 8.0 Hz, 1H, Ar–H), 7.70 (d, *J* = 6.0 Hz, 2H, Ar–H *p*-CH_3_–C_6_H_4_), 7.60–7.41 (m, 6H, Ar–H), 7.35 (d, *J* = 6.0 Hz, 2H, Ar–H *p*-CH_3_ C_6_H_4_), 4.52 (s, 2H, S–CH_2_), 2.34 (s, 3H, CH_3_). ^13^C NMR (100 MHz, *δ* ppm DMSO-*d*_6_): 160.86, 156.55, 147.31, 138.42, 135.77, 135.05, 134.33, 130.29, 130.04, 129.59, 129.50, 126.64, 126.27, 126.17, 122.15, 122.14, 120.01, 119.70, 26.73, 20.61. LC-MS (*m*/*z*) for: C_24_H_19_N_5_OS (exact mass = 425.13): calculated [M + H]^+^: 426.13; found [M + H]^+^: 426.10.

##### 2.1.1.5. 2-(((1-Phenyl-1*H*-1,2,3-triazol-4-yl)methyl)thio)-3-(*p*-tolyl)quinazolin-4(3*H*)-one (8e)

Yield: 0.27 g (75%), white solid, m.p: 139–141 °C, *R*_f_. 0.57 (hexane : ethyl acetate, 1 : 2, v/v). ^1^H NMR (400 MHz, *δ* ppm DMSO-*d*_6_): 8.75 (s, 1H, triazole CH), 8.09 (d, *J* = 7.7 Hz, 1H, Ar–H), 7.91–7.80 (m, 3H, Ar–H), 7.77 (d, *J* = 8.0 Hz, 1H, Ar–H), 7.58 (t, *J* = 7.6 Hz, 2H, Ar–H), 7.51–7.45 (m, 2H, Ar–H), 7.36 (d, *J* = 8.0 Hz, 2H, Ar–H *p*-CH_3_ C_6_H_4_), 7.33 (d, *J* = 8.0 Hz, 2H, Ar–H *p*-CH_3_ C_6_H_4_), 4.53 (s, 2H, S–CH_2_), 2.40 (s, 3H, CH_3_). ^13^C NMR (100 MHz, *δ* ppm DMSO-*d*_6_): 160.90, 156.83, 147.33, 143.68, 139.75, 136.57, 135.00, 133.14, 130.08, 129.95, 129.20, 128.77, 126.64, 126.26, 126.12, 122.22, 120.13, 119.69, 26.73, 20.90. LC-MS (*m*/*z*) for: C_24_H_19_N_5_OS (exact mass = 425.13); calculated [M + H]^+^: 426.13; found [M + H]^+^: 426.10.

##### 2.1.1.6. 2-(((1-(4-Chlorophenyl)-1*H*-1,2,3-triazol-4-yl)methyl)thio)-3-(*p*-tolyl)quinazolin-4(3*H*)-one (8f)

Yield: 0.30 g (76%), white solid, m.p: 158–160 °C, *R*_f_. 0.54 (hexane : ethyl acetate, 1 : 2, v/v). ^1^H NMR (400 MHz, *δ* ppm CDCl_3_): 8.28 (d, *J* = 7.2 Hz, 1H, Ar–H), 8.05 (s, 1H, triazole CH), 7.76 (t, *J* = 6.4 Hz, 1H, Ar–H), 7.69 (d, *J* = 7.6 Hz, 1H, Ar–H), 7.64 (d, *J* = 7.8 Hz, 2H, Ar–H *p*-Cl C_6_H_4_), 7.48 (d, *J* = 7.8 Hz, 2H, Ar–H *p*-Cl C_6_H_4_), 7.40 (d, *J* = 8.0 Hz, 1H, Ar–H), 7.33 (d, *J* = 7.2 Hz, 2H, Ar–H *p*-CH_3_ C_6_H_4_), 7.19 (d, *J* = 7.2 Hz, 2H, Ar–H *p*-CH_3_ C_6_H_4_), 4.59 (s, 2H, S–CH_2_), 2.45 (s, 3H, CH_3_). ^13^C NMR (100 MHz, *δ* ppm CDCl_3_): 161.72, 156.88, 147.65, 139.97, 135.34, 134.86, 134.65, 131.04, 129.95, 129.56, 129.54, 127.54, 126.12, 126.02, 125.92, 121.70, 120.96, 120.03, 27.13, 21.37. LC-MS (*m*/*z*) for: C_24_H_18_ClN_5_OS (exact mass = 459.09); calculated [M + H]^+^: 460.09; found [M + H]^+^: 460.00.

##### 2.1.1.7. 2-(((1-(4-Methoxyphenyl)-1*H*-1,2,3-triazol-4-yl)methyl)thio)-3-(*p*-tolyl)quinazolin-4(3*H*)-one (8g)

Yield: 0.31 g (80%), white solid, m.p: 168–170 °C, *R*_f_. 0.58 (hexane : ethyl acetate, 1 : 2, v/v). ^1^H NMR (400 MHz, *δ* ppm CDCl_3_): 8.19 (d, *J* = 7.4 Hz, 1H, Ar–H), 7.89 (s, 1H, triazole CH), 7.70 (t, *J* = 7.2 Hz, 1H, Ar–H), 7.60 (d, *J* = 8.0 Hz, 1H, Ar–H), 7.50 (d, *J* = 8.8 Hz, 2H, Ar–H *p*-O–CH_3_ C_6_H_4_), 7.35 (t, *J* = 7.5 Hz, 1H, Ar–H), 7.25 (d, *J* = 8.0 Hz, 2H, Ar–H *p*-CH_3_ C_6_H_4_), 7.11 (d, *J* = 8.0 Hz, 2H, Ar–H *p*-CH_3_ C_6_H_4_), 6.91 (d, *J* = 8.8 Hz, 2H, Ar–H *p*-O–CH_3_ C_6_H_4_), 4.49 (s, 2H, S–CH_2_), 3.77 (s, 3H, O–CH_3_), 2.35 (s, 3H, CH_3_). ^13^C NMR (100 MHz, *δ* ppm CDCl_3_): 161.82, 159.88, 157.12, 147.70, 140.43, 134.78, 132.82, 130.58, 130.48, 130.41, 130.15, 128.81, 127.51, 126.01, 125.99, 122.23, 120.01, 114.77, 55.65, 27.27, 21.43. LC-MS (*m*/*z*) for: C_25_H_21_N_5_O_2_S (exact mass = 455.14); calculated [M + H]^+^: 456.14; found [M + H]^+^: 456.10.

##### 2.1.1.8. 2-(((1-(*p*-Tolyl)-1*H*-1,2,3-triazol-4-yl)methyl)thio)-3-(*p*-tolyl)quinazolin-4(3*H*)-one (8h)

Yield: 0.28 g (75%), white solid, m.p: 143–145 °C, *R*_f_. 0.56 (hexane : ethyl acetate, 1 : 2, v/v). ^1^H NMR (400 MHz, *δ* ppm CDCl_3_): 8.18 (d, *J* = 7.2 Hz, 1H, Ar–H), 7.92 (s, 1H, triazole CH), 7.67 (t, *J* = 6.4 Hz, 1H, Ar–H), 7.59 (d, *J* = 7.6 Hz, 1H, Ar–H), 7.46 (d, *J* = 6.8 Hz, 2H, *p*-CH_3_ C_6_H_4_), 7.34 (t, *J* = 6.6 Hz, 1H, Ar–H), 7.23 (d, *J* = 8.0 Hz, 2H, *p*-CH_3_ C_6_H_4_), 7.14 (d, *J* = 8.0 Hz, 2H, *p*-CH_3_ C_6_H_4_), 7.09 (d, *J* = 6.8 Hz, 2H, *p*-CH_3_ C_6_H_4_), 4.49 (s, 2H, S–CH_2_), 2.34 (s, 3H, CH_3_), 2.31 (s, 3H, CH_3_). ^13^C NMR (100 MHz, *δ* ppm CDCl_3_): 161.83, 157.13, 147.70, 140.42, 138.97, 134.80, 134.68, 132.84, 130.47, 130.24, 130.15, 128.81, 127.50, 126.02, 126.00, 121.10, 120.48, 120.01, 27.29, 21.44, 21.12. LC-MS (*m*/*z*) for: C_25_H_21_N_5_OS (exact mass = 439.15); calculated [M + H]^+^: 440.15; found [M + H]^+^: 440.20.

##### 2.1.1.9. 2-(((1-Phenyl-1*H*-1,2,3-triazol-4-yl)methyl)thio)-3-(*m*-tolyl)quinazolin-4(3*H*)-one (8i)

Yield: 0.25 g (70%), white solid, m.p: 155–157 °C, *R*_f_. 0.57 (hexane : ethyl acetate, 1 : 2, v/v). ^1^H NMR (400 MHz, *δ* ppm DMSO-*d*_6_): 8.75 (s, 1H, triazole CH), 8.08 (d, *J* = 7.8 Hz, 1H, Ar–H), 7.87–7.81 (m, 3H, Ar–H), 7.77 (d, *J* = 8.40 Hz, 1H, Ar–H), 7.56 (t, *J* = 7.6 Hz, 2H, Ar–H), 7.51–7.40 (m, 3H, Ar–H), 7.35 (d, *J* = 7.5 Hz, 1H, Ar–H), 7.25 (d, *J* = 8.0 Hz, 2H, Ar–H), 4.53 (s, 2H, S–CH_2_), 2.36 (s, 3H, CH_3_). ^13^C NMR (100 MHz, *δ* ppm DMSO-*d*_6_): 160.77, 156.56, 147.27, 143.58, 139.15, 136.54, 135.64, 134.96, 130.64, 129.90, 129.66, 129.33, 128.71, 126.58, 126.44, 126.24, 126.09, 122.20, 120.08, 119.66, 26.70, 20.78. LC-MS (*m*/*z*) for: C_24_H_19_N_5_OS (exact mass = 425.13); calculated [M + H]^+^: 426.13; found [M + H]^+^: 426.10.

##### 2.1.1.10. 2-(((1-(4-Chlorophenyl)-1*H*-1,2,3-triazol-4-yl)methyl)thio)-3-(*m*-tolyl)quinazolin-4(3*H*)-one (8j)

Yield: 0.28 g (72%), white solid, m.p: 177–179 °C, *R*_f_. 0.54 (hexane : ethyl acetate, 1 : 2, v/v). ^1^H NMR (400 MHz, *δ* ppm CDCl_3_): 8.19 (d, *J* = 7.7 Hz, 1H, Ar–H), 7.96 (s, 1H, triazole CH), 7.70 (t, *J* = 7.3 Hz, 1H, Ar–H), 7.60 (d, *J* = 8.0 Hz, 1H, Ar–H), 7.55 (d, *J* = 8.4 Hz, 2H, Ar–H *p*-Cl C_6_H_4_), 7.39 (d, *J* = 8.4 Hz, 2H, Ar–H *p*-Cl C_6_H_4_), 7.35 (d, *J* = 7.2 Hz, 1H, Ar–H), 7.32 (d, *J* = 8.0 Hz, 1H, Ar–H), 7.25 (d, *J* = 7.6 Hz, 1H, Ar–H), 7.02 (d, *J* = 6.4 Hz, 2H, Ar–H), 4.49 (s, 2H, S–CH_2_), 2.33 (s, 3H, CH_3_). ^13^C NMR (100 MHz, *δ* ppm CDCl_3_): 161.77, 157.03, 147.66, 145.10, 140.48, 135.41, 134.83, 134.58, 132.79, 130.49, 130.14, 129.93, 128.79, 128.06, 127.53, 126.08, 125.94, 121.69, 120.93, 120.01, 27.16, 21.44. LC-MS (*m*/*z*) for: C_24_H_18_ClN_5_OS (exact mass = 459.09); calculated [M + H]^+^: 460.09; found [M + H]^+^: 460.10.

##### 2.1.1.11. 2-(((1-(4-Methoxyphenyl)-1*H*-1,2,3-triazol-4-yl)methyl)thio)-3-(*m*-tolyl)quinazolin-4(3*H*)-one (8k)

Yield: 0.28 g (73%), white solid, m.p: 186–188 °C, *R*_f_. 0.58 (hexane : ethyl acetate, 1 : 2, v/v). ^1^H NMR (400 MHz, *δ* ppm CDCl_3_): 8.28 (d, *J* = 5.6 Hz, 1H, Ar–H), 8.00 (s, 1H, triazole CH), 7.78 (s, 1H, Ar–H), 7.71 (s, 1H, Ar–H), 7.58 (d, *J* = 5.8 Hz, 2H, Ar–H *p*-OCH_3_ C_6_H_4_), 7.43 (d, *J* = 6.4 Hz, 2H, Ar–H), 7.34 (s, 1H, Ar–H), 7.12 (s, 2H, Ar–H), 6.99 (d, *J* = 5.8 Hz, 2H, Ar–H *p*-OCH_3_ C_6_H_4_), 4.61 (s, 2H, S–CH_2_), 3.86 (s, 3H, OCH_3_), 2.42 (s, 3H, CH_3_). ^13^C NMR (100 MHz, *δ* ppm CDCl_3_): 161.76, 159.87, 157.00, 147.69, 139.93, 135.39, 134.82, 131.00, 130.38, 129.55, 129.54, 129.27, 127.47, 126.03, 126.01, 125.88, 125.34, 122.21, 120.00, 114.76, 55.65, 27.31, 21.38. LC-MS (*m*/*z*) for: C_25_H_21_N_5_O_2_S (exact mass = 455.14); calculated [M + H]^+^: 456.14; found [M + H]^+^: 456.10.

##### 2.1.1.12. 2-(((1-(*p*-Tolyl)-1*H*-1,2,3-triazol-4-yl)methyl)thio)-3-(*m*-tolyl)quinazolin-4(3*H*)-one (8l)

Yield: 0.26 g (70%), white solid, m.p: 158–160 °C, *R*_f_. 0.56 (hexane : ethyl acetate, 1 : 2, v/v). ^1^H NMR (400 MHz, *δ* ppm CDCl_3_): 8.17 (s, 1H, Ar–H), 7.93 (s, 1H, triazole CH), 7.67 (s, 1H, Ar–H), 7.60 (s, 1H, Ar–H), 7.45 (s, 2H, Ar–H *p*-CH_3_ C_6_H_4_), 7.32 (s, 2H, Ar–H), 7.19 (s, 3H, Ar–H), 7.02 (s, 2H, Ar–H *p*-CH_3_ C_6_H_4_), 4.48 (s, 2H, S–CH_2_), 2.31 (s, 6H, 2CH_3_). ^13^C NMR (100 MHz, *δ* ppm CDCl_3_): 161.78, 157.01, 147.71, 139.93, 138.93, 135.49, 135.41, 135.34, 134.82, 134.72, 130.99, 130.24, 129.56, 129.55, 127.47, 126.19, 126.04, 125.99, 120.47, 120.01, 27.30, 21.38, 21.12. LC-MS (*m*/*z*) for: C_25_H_21_N_5_OS (exact mass = 439.15); calculated [M + H]^+^: 440.15; found [M + H]^+^: 440.00.

##### 2.1.1.13. 2-(((1-Phenyl-1*H*-1,2,3-triazol-4-yl)methyl)thio)-3-ethyl quinazolin-4(3*H*)-one (8m)

Yield: 0.21 g (67%), white solid, m.p: 120–122 °C, *R*_f_. 0.51 (hexane : ethyl acetate, 1 : 2, v/v). ^1^H NMR (400 MHz, *δ* ppm DMSO-*d*_6_): 8.82 (s, 1H, triazole CH), 8.08 (d, *J* = 7.7 Hz, 1H, Ar–H), 7.86 (d, *J* = 7.0 Hz, 1H, Ar–H), 7.68–7.60 (m, 4H, Ar–H), 7.48 (t, *J* = 7.2 Hz, 1H, Ar–H), 7.30 (d, *J* = 8.4 Hz, 2H, Ar–H), 4.70 (s, 2H, S–CH_2_), 4.08 (q, *J* = 6.8 Hz, 2H, N–CH_2_), 1.16 (t, *J* = 6.8 Hz, 3H, CH_3_). ^13^C NMR (100 MHz, *δ* ppm DMSO-*d*_6_): 161.29, 155.65, 147.31, 143.74, 138.96, 135.66, 134.42, 130.33, 127.32, 125.95, 125.83, 123.12, 121.58, 119.85, 39.85, 26.68, 13.29.

##### 2.1.1.14. 2-(((1-(4-Chlorophenyl)-1*H*-1,2,3-triazol-4-yl)methyl)thio)-3-ethyl quinazolin-4(3*H*)-one (8n)

Yield: 0.23 g (69%), white solid, m.p: 132–134 °C, *R*_f_. 0.53 (hexane : ethyl acetate, 1 : 2, v/v). ^1^H NMR (400 MHz, *δ* ppm CDCl_3_): 8.15 (dd, *J* = 8.0, 1.1 Hz, 1H, Ar–H), 7.99 (s, 1H, triazole CH), 7.69–7.61 (m, 1H, Ar–H), 7.55 (d, *J* = 8.8 Hz, 2H, Ar–H *p*-Cl C_6_H_4_), 7.52 (s, 1H, Ar–H), 7.38 (d, *J* = 8.8 Hz, 2H, Ar–H *p*-Cl C_6_H_4_), 7.35–7.30 (m, 1H, Ar–H), 4.63 (s, 2H, S–CH_2_), 4.10 (q, *J* = 7.1 Hz, 2H, N–CH_2_), 1.29 (t, *J* = 7.1 Hz, 3H, CH_3_). ^13^C NMR (100 MHz, *δ* ppm CDCl_3_): 161.32, 155.62, 147.21, 135.45, 134.59, 134.49, 132.99, 129.93, 127.19, 125.95, 125.63, 124.92, 121.71, 119.56, 39.87, 26.55, 13.27. LC-MS (*m*/*z*) for: C_19_H_16_ClN_5_OS (exact mass = 397.08); calculated [M + H]^+^: 398.08; found [M + H]^+^: 398.00.

##### 2.1.1.15. 2-(((1-(4-Methoxyphenyl)-1*H*-1,2,3-triazol-4-yl)methyl)thio)-3-ethyl quinazolin-4(3*H*)-one (8o)

Yield: 0.23 g (69%), white solid, m.p: 142–144 °C, *R*_f_. 0.49 (hexane : ethyl acetate, 1 : 2, v/v). ^1^H NMR (400 MHz, *δ* ppm CDCl_3_): 8.15 (d, *J* = 7.6 Hz, 1H, Ar–H), 7.92 (s, 1H, triazole CH), 7.64 (t, *J* = 7.2 Hz, 1H, Ar–H), 7.49 (d, *J* = 8.5 Hz, 2H, Ar–H *p*-OCH_3_ C_6_H_4_), 7.32 (t, *J* = 7.4 Hz, 1H, Ar–H), 7.21 (d, *J* = 8.0 Hz, 1H, Ar–H), 6.90 (d, *J* = 8.5 Hz, 2H, Ar–H *p*-OCH_3_ C_6_H_4_), 4.62 (s, 2H, S–CH_2_), 4.10 (q, *J* = 6.8 Hz, 2H, N–CH_2_), 3.76 (s, 3H, OCH_3_), 1.28 (t, *J* = 6.8 Hz, 3H, CH_3_). ^13^C NMR (100 MHz, *δ* ppm CDCl_3_): 161.38, 159.85, 155.66, 149.57, 147.31, 134.42, 128.64, 127.13, 125.85, 123.33, 122.22, 119.56, 115.26, 114.75, 55.63, 39.83, 26.68, 13.26. LC-MS (*m*/*z*) for: C_20_H_19_N_5_O_2_S (exact mass = 393.13); calculated [M + H]^+^: 394.13; found [M + H]^+^: 394.00.

##### 2.1.1.16. 2-(((1-(*p*-Tolyl)-1*H*-1,2,3-triazol-4-yl)methyl)thio)-3-ethylquinazolin-4(3*H*)-one (8p)

Yield: 0.22 g (68%), white solid, m.p: 125–127 °C, *R*_f_. 0.52 (hexane : ethyl acetate, 1 : 2, v/v). ^1^H NMR (400 MHz, *δ* ppm CDCl_3_): 8.14 (dd, *J* = 7.9, 0.9 Hz, 1H, Ar–H), 7.95 (s, 1H, triazole CH), 7.66–7.59 (m, 1H, Ar–H), 7.52 (d, *J* = 8.0 Hz, 1H, Ar–H), 7.46 (d, *J* = 8.4 Hz, 2H, Ar–H *p*-CH_3_ C_6_H_4_), 7.34–7.27 (m, 1H, Ar–H), 7.19 (d, *J* = 8.4 Hz, 2H, Ar–H *p*-CH_3_ C_6_H_4_), 4.62 (s, 2H, S–CH_2_), 4.09 (q, *J* = 7.1 Hz, 2H, N–CH_2_), 2.31 (s, 3H, benzylic CH_3_), 1.28 (t, *J* = 7.1 Hz, 3H, CH_3_). ^13^C NMR (100 MHz, *δ* ppm CDCl_3_): 161.37, 155.65, 147.31, 144.63, 138.97, 134.66, 134.42, 130.23, 127.12, 125.84, 125.74, 121.11, 120.48, 119.56, 39.82, 26.66, 21.10, 13.26. LC-MS (*m*/*z*) for: C_20_H_19_N_5_OS (exact mass = 377.13); calculated [M + H]^+^: 378.13; found [M + H]^+^: 378.10.

##### 2.1.1.17. 2-(((1-Phenyl-1*H*-1,2,3-triazol-4-yl)methyl)thio)-3-allyl quinazolin-4(3*H*)-one (8q)

Yield: 0.21 g (66%), white solid, m.p: 124–126 °C, *R*_f_. 0.50 (hexane : ethyl acetate, 1 : 2, v/v). ^1^H NMR (400 MHz, *δ* ppm DMSO-*d*_6_): 8.70 (s, 1H, triazole CH), 8.06 (d, *J* = 7.8 Hz, 1H, Ar–H), 7.96 (d, *J* = 6.8 Hz, 2H, Ar–H), 7.74 (t, *J* = 7.5 Hz, 1H, Ar–H), 7.61–7.50 (m, 3H, Ar–H), 7.45 (t, *J* = 7.5 Hz, 1H, Ar–H), 7.35 (d, *J* = 8.1 Hz, 1H, Ar–H), 5.96–5.82 (m, 1H, =CH), 5.21 (d, *J* = 10.0 Hz, 1H, =CH_2_), 5.11 (d, *J* = 16.0 Hz, 1H, =CH_2_), 4.90 (s, 2H, S–CH_2_), 4.74 (d, *J* = 4.0 Hz, 2H, N–CH_2_); ^13^C NMR (100 MHz, *δ* ppm DMSO-*d*_6_): 160.21, 154.96, 146.46, 138.97, 134.94, 131.63, 131.26, 129.31, 126.96, 126.54, 126.36, 126.02, 125.71, 120.62, 118.74, 117.62, 45.96, 26.80.

##### 2.1.1.18. 2-(((1-(4-Chlorophenyl)-1*H*-1,2,3-triazol-4-yl)methyl)thio)-3-allyl quinazolin-4(3*H*)-one (8r)

Yield: 0.25 g (71%), white solid, m.p: 137–139 °C, *R*_f_. 0.54 (hexane : ethyl acetate, 1 : 2, v/v). ^1^H NMR (400 MHz, *δ* ppm CDCl_3_): 8.15 (d, *J* = 7.6 Hz, 1H, Ar–H), 7.97 (s, 1H, triazole CH), 7.65 (t, *J* = 7.4 Hz, 1H, Ar–H), 7.54 (d, *J* = 8.4 Hz, 2H, Ar–H *p*-Cl C_6_H_4_), 7.52 (s, 1H, Ar–H), 7.38 (d, *J* = 8.4 Hz, 2H, Ar–H *p*-Cl C_6_H_4_), 7.33 (d, *J* = 7.6 Hz, 1H, Ar–H), 5.91–5.76 (m, 1H, =CH), 5.20 (d, *J* = 12.0 Hz, 1H, =CH_2_), 5.17 (d, *J* = 6.0 Hz, 1H, =CH_2_), 4.67 (d, *J* = 5.1 Hz, 2H, N–CH_2_), 4.63 (s, 2H, S–CH_2_). ^13^C NMR (100 MHz, *δ* ppm CDCl_3_): 161.35, 155.75, 147.26, 144.87, 135.41, 134.63, 130.26, 129.94, 127.32, 126.25, 126.04, 125.75, 121.70, 121.07, 119.47, 118.59, 46.39, 26.72. LC-MS (*m*/*z*) for: C_20_H_16_ClN_5_OS (exact mass = 409.08); calculated [M + H]^+^: 410.08; found [M + H]^+^: 410.00.

##### 2.1.1.19. 2-(((1-(4-Methoxyphenyl)-1*H*-1,2,3-triazol-4-yl)methyl)thio)-3-allyl quinazolin-4(3*H*)-one (8s)

Yield: 0.25 g (72%), white solid, m.p: 148–150 °C, *R*_f_. 0.48 (hexane : ethyl acetate, 1 : 2, v/v). ^1^H NMR (400 MHz, *δ* ppm DMSO-*d*_6_): 8.70 (s, 1H, triazole CH), 8.08 (s, 1H, Ar–H), 7.81–7.70 (m, 4H, Ar–H), 7.48 (s, 1H, Ar–H), 7.12 (s, 2H, Ar–H), 5.95–5.85 (m, 1H, =CH), 5.16 (d, *J* = 16.0 Hz, 1H, =CH_2_), 5.12 (d, *J* = 8.0 Hz, 1H, =CH_2_), 4.68 (s, 4H, N–CH_2_ & S–CH_2_), 3.81 (s, 3H, OCH_3_). ^13^C NMR (100 MHz, *δ* ppm DMSO-*d*_6_): 161.63, 159.95, 154.98, 149.57, 146.73, 134.82, 131.24, 128.64, 126.89, 126.13, 125.74, 122.25, 120.22, 119.28, 117.56, 114.69, 55.37, 45.93, 26.70. LC-MS (*m*/*z*) for: C_21_H_19_N_5_O_2_S (exact mass = 405.13); calculated [M + H]^+^: 406.13; found [M + H]^+^: 406.10.

##### 2.1.1.20. 2-(((1-(*p*-Tolyl)-1*H*-1,2,3-triazol-4-yl)methyl)thio)-3-allyl quinazolin-4(3*H*)-one (8t)

Yield: 0.23 g (69%), white solid, m.p: 130–132 °C, *R*_f_. 0.53 (hexane : ethyl acetate, 1 : 2, v/v). ^1^H NMR (400 MHz, *δ* ppm CDCl_3_): 8.15 (d, *J* = 7.6 Hz, 1H, Ar–H), 7.94 (s, 1H, triazole CH), 7.64 (t, *J* = 7.4 Hz, 1H, Ar–H), 7.53 (d, *J* = 8.0 Hz, 1H, Ar–H), 7.46 (d, *J* = 8.4 Hz, 2H, Ar–H *p*-CH_3_ C_6_H_4_), 7.32 (t, *J* = 7.6 Hz, 1H, Ar–H), 7.18 (d, *J* = 8.4 Hz, 2H, Ar–H *p*-CH_3_ C_6_H_4_), 5.94–5.73 (m, 1H, =CH), 5.20 (d, *J* = 14.0 Hz, 1H, =CH_2_), 5.16 (d, *J* = 6.8 Hz, 1H, =CH_2_), 4.67 (d, *J* = 5.2 Hz, 2H, N–CH_2_), 4.62 (s, 2H, S–CH_2_), 2.31 (s, 3H, CH_3_). ^13^C NMR (100 MHz, *δ* ppm CDCl_3_): 161.38, 155.87, 147.30, 144.50, 138.96, 134.67, 134.58, 130.54, 130.23, 127.27, 125.96, 125.81, 121.12, 120.47, 119.46, 118.78, 46.37, 26.85, 21.10. LC-MS (*m*/*z*) for: C_21_H_19_N_5_OS (exact mass = 389.13); calculated [M + H]^+^: 390.13; found [M + H]^+^: 390.10.

### 2.2. Biology

#### 2.2.1. Cell viability assay

The viability of new compounds 8a–t was evaluated using the MCF-10A normal human mammary gland epithelial cell line. The cell viability of compounds 8a–t was examined using the MTT assay^36^ after four days of incubation with MCF-10A cells. For more details, see Appendix A (ESI file).†

#### 2.2.2. Antiproliferative assay

The MTT assay was used to explore the antiproliferative efficacy of 8a–t against the four human cancer cell lines, using Erlotinib as a control.^37^ The median inhibitory concentration (IC_50_) and GI_50_ (mean IC_50_) for the four cancer cell lines were calculated. Appendix A† contains more details.

#### 2.2.3. EGFR inhibitory assay

The EGFR-TK assay was employed^38^ to evaluate the inhibitory efficacy of the most potent antiproliferative derivatives 8f, 8g, 8h, 8j, and 8l against EGFR. See Appendix A† for more information.

#### 2.2.4. BRAF^V600E^ inhibitory assay

An *in vitro* study evaluated the anti-BRAF^V600E^ efficacy of compounds 8f, 8g, 8h, 8j, and 8l according to reported procedures.^39^ See Appendix A† for more details.

#### 2.2.5. Apoptotic markers assays

Compounds 8g and 8h were evaluated as caspase-3, 8 and Bax activators and as down-regulator of the anti-apoptotic protein Bcl2 against the MCF-7 breast cancer cell line.^40^ Appendix A† contains more details.

#### 2.2.6. Cell cycle analysis and apoptosis detection

The impact of compound 8g on cell cycle progression and apoptosis induction was examined in A-549 cells. A lung cancer (A-549) cell line was subjected to treatment for 24 hours with an IC_50_ concentration of 8g. We stained the cell line with PI/annexin V and performed flow cytometry using a BD FACS Caliber.^41^ Refer to Appendix A† for more details.

### 2.3. Docking study

Molecular docking simulations within EGFR (PDB ID: 1M17)^42^ and BRAF^V600E^ (PDB ID: 4RZV).^43^ Were validated with a redocking test, whereby the test proteins' structures had remained fixed while the co-crystallized ligands (Erlotinib for EGFR and Vemurafenib for BRAF^V600E^) were redocked into their crystal-binding pocket. See Appendix A† for more details.

## 3. Results and discussion

### 3.1. Chemistry

**Scheme 1:**
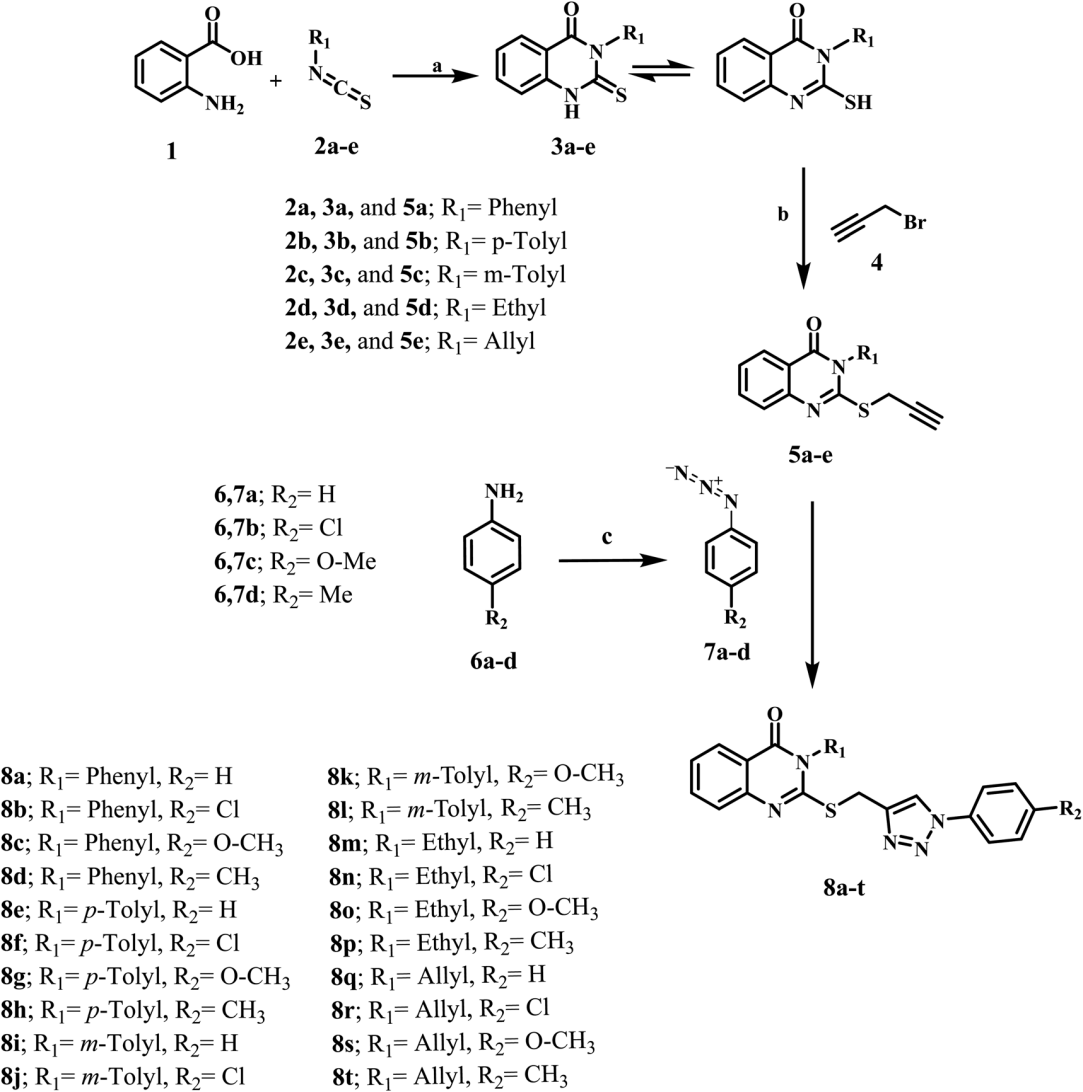
Synthesis of the new target compounds 8a–t. Reagents and conditions: (a) TEA, ethanol, reflux (8–12 h), (b) K_2_CO_3_, DMF, stirring (24 h), (c) 1-NaNO_2_, HCl, 2-NaN_3_ (d) sodium ascorbate, CuSO_4_·5H_2_O, THF, reflux (24–48 h).

Scheme 1 depicts the synthetic methods for the novel target hybrids (8a–t). We refluxed anthranilic acid (1) in ethanol with isothiocyanate derivatives (2a–e) for 8 h. After the reaction was complete (as determined by TLC), the resulting white precipitate was filtered and recrystallized from an ethanol/dioxane mixture (1 : 1), yielding quinazoline-4-ones (3a–e) in 90–95% yields.^44^ The quinazoline derivatives (3a–e) were then alkylated with propargyl bromide (4) in the presence of anhydrous K_2_CO_3_ by stirring in DMF, yielding S-propargyl derivatives (5a–e) in 70–83% yields that were purified by ethanol recrystallization.^45^ FTIR spectrum of 5c, as an example, revealed the appearance of the characteristic peak of ≡C–H stretching at *ν* 3245 cm^−1^, 3066 (=C–H stretching), 2968, 2928 (sp^3^C–H stretching), 2116 (C≡C) and 1687 (C=O). The diazotization of aniline derivatives (6a–d) with NaNO_2_/HCl at 0–5 °C, followed by the addition of sodium azide, resulted in the formation of the corresponding aromatic azides (7a–d) with a yield of 70–75%.^46^ The FTIR spectrum of 7c confirmed the appearance of the characteristic peak of the azide group at 2109 cm^−1^.

1,3-Dipolar cycloaddition reactions between propargyl derivatives (5a–e) and aromatic azides (7a–d) were carried out by refluxing in THF in the presence of CuSO_4_·5H_2_O/sodium ascorbate as a catalyst, yielding 1,4-disubstituted 1,2,3-triazoles (8a–t) in good yields (86–90%). The structures of 8a–t were elucidated using ^1^H NMR, ^13^C NMR spectroscopy, and LC-MS. The ^1^H NMR spectra of compound 8g, as an example, confirmed the presence of three characteristic singlet signals in the aliphatic range: first one at *δ* 2.36 (s, 3H, C*H*_3_), second one at *δ* 3.77 (s, 3H, O–C*H*_3_) and third one at *δ* 4.49 (s, 2H, S–C*H*_2_). Additionally, the spectrum revealed another characteristic singlet signal in the aromatic range at *δ* 7.89 of the triazole CH (s, 1H, triazole C*H*).

Also, the spectrum revealed two pairs of doublets of the aromatic ring's *p*-disubstituted patterns and extra signals for the aromatic protons in the quinazoline moiety. The ^13^C NMR spectrum of 8g indicated the presence of peaks of methoxy, methylene, and methyl groups at *δ* 55.65, *δ* 27.27, and *δ* 21.43 ppm, respectively. The ^13^C NMR spectrum of 8g also indicated a peak at *δ* 161.82 ppm of the amidic carbonyl group. LC-MS spectrum of compound 8g (C_25_H_21_N_5_O_2_S, M. Wt = 455.14) showed a signal at 456.10 *m*/*z* [M + H]^+^.

### 3.2. Biology

#### 3.2.1. Cell viability assay

We evaluated the viability of new compounds 8a–t using the MCF-10A normal human mammary gland epithelial cell line. The cell viability of compounds 8a–t was examined using the MTT assay^36^ after four days of incubation with MCF-10A cells. Table 1 indicates that none of the compounds analyzed exhibited cytotoxicity, with all hybrids maintaining above 84% cell viability at a concentration of 50 μM.

**Table 1:** Cell viability % and IC_50_ values of compounds 8a–t against four cancer cell lines using MTT assay

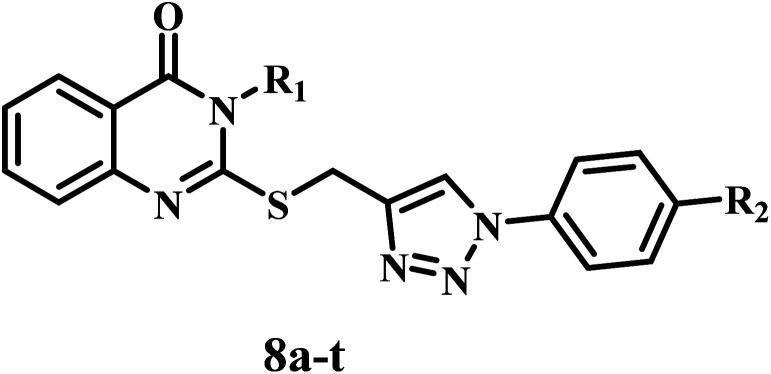								
Comp.	Cell viability %	R_1_	R_2_	Antiproliferative activity IC_50_ ± SEM (nM)				
				A-549	MCF-7	Panc-1	HT-29	Average (GI_50_)
8a	89	Phenyl	H	61 ± 5	56 ± 5	64 ± 5	64 ± 5	61
8b	90	Phenyl	Cl	40 ± 3	36 ± 3	42 ± 3	44 ± 3	41
8c	86	Phenyl	OMe	47 ± 3	42 ± 3	49 ± 3	49 ± 3	47
8d	89	Phenyl	Me	53 ± 4	48 ± 4	56 ± 4	56 ± 4	53
8e	90	*p*-Tolyl	H	88 ± 8	85 ± 8	92 ± 8	92 ± 8	89
8f	87	*p*-Tolyl	Cl	37 ± 3	34 ± 3	38 ± 3	39 ± 3	37
8g	88	*p*-Tolyl	OMe	22 ± 1	20 ± 1	24 ± 1	24 ± 1	22
8h	92	*p*-Tolyl	Me	25 ± 1	22 ± 1	26 ± 1	27 ± 1	25
8i	90	*m*-Tolyl	H	65 ± 5	59 ± 5	68 ± 6	68 ± 6	65
8j	87	*m*-Tolyl	Cl	29 ± 2	26 ± 2	31 ± 2	30 ± 2	29
8k	85	*m*-Tolyl	OMe	42 ± 3	39 ± 3	46 ± 3	45 ± 3	43
8l	87	*m*-Tolyl	Me	33 ± 2	29 ± 2	36 ± 2	35 ± 2	33
8m	90	Ethyl	H	72 ± 6	68 ± 6	74 ± 6	74 ± 6	72
8n	92	Ethyl	Cl	75 ± 6	71 ± 6	78 ± 6	79 ± 6	76
8o	89	Ethyl	OMe	69 ± 5	63 ± 5	74 ± 6	74 ± 6	70
8p	86	Ethyl	Me	82 ± 7	77 ± 7	84 ± 7	85 ± 7	82
8q	87	Allyl	H	93 ± 8	89 ± 8	96 ± 8	96 ± 8	94
8r	90	Allyl	Cl	79 ± 6	75 ± 6	81 ± 7	84 ± 7	80
8s	91	Allyl	OMe	57 ± 5	53 ± 4	59 ± 5	59 ± 5	57
8t	90	Allyl	Me	84 ± 7	80 ± 7	87 ± 7	87 ± 7	85
Erlotinib	ND	—	—	30 ± 3	40 ± 3	30 ± 3	30 ± 3	33

#### 3.2.2. Antiproliferative assay

The MTT assay was used to test the antiproliferative efficacy of hybrids 8a–t against four human cancer cell lines: colon cancer (HT-29), pancreatic cancer (Panc-1), lung cancer (A-549), and breast cancer (MCF-7), using Erlotinib as a control.^37,47^Table 1 presents the median inhibitory concentration (IC50) and GI50 (mean IC50) for the four cancer cell lines.

Compared to Erlotinib, which had a GI_50_ of 33 nM, compounds 8a–t had substantial antiproliferative action, with GI_50_ values ranging from 22 nM to 94 nM *versus* the four cancer cell lines evaluated. In that order, the most potent derivatives were 8f, 8g, 8h, 8j, and 8l, with GI_50_ values of 37, 22, 25, 29, and 33 nM. This means that 8g, 8h, and 8j were stronger than Erlotinib (GI_50_ = 33 nM). The most potent of the newly synthesized hybrids 8a–t was compound 8g (R_1_ = *p*-tolyl, R_2_ = OMe), which had a GI_50_ value of 22 nM, which is 1.5 times stronger than the standard Erlotinib (GI_50_ = 33 nM). The nature of the aryl/alkyl substituents at position 3 of the quinazoline moiety appears to be critical for the 8a–t hybrids' antiproliferative activity. The GI_50_ values of compounds 8h (R_1_ = *p*-tolyl, R_2_ = Me) and 8j (R_1_ = *m*-tolyl, R_2_ = Cl) were 25 nM and 29 nM, respectively. These values were lower than compound 8g's (GI_50_ = 22 nM) but higher than the reference erlotinib. Also, compounds 8k (R_1_ = *m*-tolyl, R_2_ = OMe) and 8l (R_1_ = *m*-tolyl, R_2_ = Me) had GI_50_ values of 43 and 33 nM, respectively. These were less potent than compounds 8g, 8h, and 8j, but 8l performed similarly to erlotinib, while 8k is less effective than erlotinib. These data demonstrated that the *p*-tolyl group at position 3 of the quinazoline moiety is more tolerated for antiproliferative action than the *m*-tolyl one. Additionally, the GI_50_ values for compounds 8c (R_1_ = phenyl, R_2_ = OMe), 8o (R_1_ = ethyl, R_2_ = OMe), and 8s (R_1_ = allyl, R_2_ = OMe) were 47, 70, and 57 nM, respectively. These values are lower than those for compound 8g (R_1_ = *p*-tolyl, R_2_ = OMe) and even Erlotinib. The results highlighted the importance of the substitution pattern at position three of the quinazoline moiety in antiproliferative activity, with efficacy increasing in the following order: *p*-tolyl > *m*-tolyl > phenyl > allyl > ethyl.

Also, the pattern of substitution at the fourth position of the phenyl group within the 1,2,3-triazole moiety (R_2_) may significantly impact how effectively 8a–t hybrids inhibit cell proliferation. The GI_50_ values for compounds 8e (R_1_ = *p*-tolyl, R_2_ = H), 8f (R_1_ = *p*-tolyl, R_2_ = Cl), and 8h (R_1_ = *p*-tolyl, R_2_ = Me) were 89, 37, and 25 nM, respectively, demonstrating lower potency than 8g (R_1_ = *p*-tolyl, R_2_ = OMe), which exhibited a GI_50_ value of 22 nM. The results show that the antiproliferative activity of these hybrids is affected by the pattern of substitutions at the fourth position of the phenyl group in the 1,2,3-triazole moiety. The activity decreases from OMe to Me to Cl, with compound 8e, the unsubstituted derivative (R_2_ = H), having the least potency. It is 4-fold less potent than compound 8g, the methoxy derivative.

Another significant feature is that all tested compounds exhibited heightened sensitivity to the breast cancer (MCF-7) cell line compared to the other cell lines investigated. For example, compound 8g (R_1_ = *p*-tolyl, R_2_ = OMe) had the most activity, with IC_50_ values of 22, 20, 24, and 24 nM against lung cancer-A-549, breast cancer-MCF-7, pancreatic cancer-Panc-1 pancreatic, and colon cancer-HT-29 cancer cell lines, respectively. It was more effective than erlotinib against all four cancer cell lines and twice as effective against the MCF-7 breast cancer cell line. The same rule applies to all derivatives, regardless of the characteristics of (R_2_) or how the quinazoline moiety is substituted at position 3.

#### 3.2.3. EGFR inhibitory assay

We employed the EGFR-TK assay^38,48^ to evaluate the inhibitory efficacy of the most potent antiproliferative derivatives 8f, 8g, 8h, 8j, and 8l against EGFR, and presented the results in Table 2. The results of this test are the same as the results of the antiproliferative test, which found that compounds 8g (R_1_ = *p*-tolyl, R_2_ = OMe), 8h (R_1_ = *p*-tolyl, R_2_ = Me), and 8j (R_1_ = *m*-tolyl, R_2_ = Cl) were the best antiproliferative agent. With IC_50_ values of 68 ± 4 nM, 74 ± 5 nM, and 78 ± 5 nM, these compounds were the most effective EGFR inhibitor derivatives. They worked better than the standard drug Erlotinib (IC_50_ = 80 ± 5 nM). Compounds 8f (R_1_ = *p*-tolyl, R_2_ = Cl) and 8l (R_1_ = *m*-tolyl, R_2_ = Me) had strong anti-EGFR activity, with IC_50_ values of 89 ± 5 nM and 83 ± 5 nM, respectively. These values are the same as Erlotinib's IC_50_ value of 80 nM. These results indicate that compounds 8g, 8h, and 8j exhibit substantial EGFR inhibitory activity and may serve as potential antiproliferative agents.

**Table 2:** IC_50_ values of compounds 8f, 8g, 8h, 8j, and 8l against EGFR and BRAF^V600E^ against MCF-7 cancer cell line

Compound	EGFR inhibition IC_50_ ± SEM (nM)	BRAF^V600E^ inhibition IC_50_ ± SEM (nM)
8f	89 ± 5	69 ± 5
8g	68 ± 4	47 ± 3
8h	74 ± 5	55 ± 5
8j	78 ± 5	61 ± 5
8l	83 ± 5	64 ± 5
Erlotinib	80 ± 5	60 ± 5
Vemurafenib	ND	30 ± 3

#### 3.2.4. BRAF^V600E^ inhibitory assay

An *in vitro* study evaluated the anti-BRAF^V600E^ efficacy of compounds 8f, 8g, 8h, 8j, and 8l.^39^ The enzyme test showed that the five hybrids tested strongly blocked BRAF^V600E^, with IC_50_ values ranging from 47 to 69 nM, as shown in Table 2. In all cases, the IC_50_ of the analyzed compounds exceeds that of the reference Vemurafenib (IC_50_ = 30 nM), indicating reduced potency. Compounds 8g, 8h, and 8j demonstrated the highest inhibitory efficacy against BRAF^V600E^ (IC_50_ = 47, 55, and 61 nM, respectively), and demonstrated significant inhibition of cancer cell growth (GI_50_ = 22, 25, and 33 nM, respectively). As a result, compounds 8g, 8h, and 8j are potential antiproliferative agents that operate as dual inhibitors of EGFR and BRAF^V600E^.

#### 3.2.5. Assay for the activation of apoptotic markers

Apoptosis, or programmed cell death, involves numerous biochemical and morphological mechanisms.^49,50^ Antiapoptotic proteins, like Bcl-2 and Bc-W, coexist with proapoptotic proteins such as Bad and Bax.^51,52^ Pro-apoptotic proteins promote cytochrome c release, while anti-apoptotic proteins regulate apoptosis by preventing cytochrome c release. The outer mitochondrial membrane becomes permeable when the concentration of proapoptotic proteins surpasses that of antiapoptotic proteins, initiating a cascade of events. The release of cytochrome c activates caspase-3 and caspase-9. Caspase-3 initiates apoptosis by targeting multiple essential proteins that are required for cellular function.^53,54^

We further investigated the ability of compounds 8g and 8h, the most effective in all *in vitro* studies, to activate apoptotic markers.

##### 3.2.5.1. Caspase-3 activation assay

Compounds 8g and 8h were evaluated as caspase-3 activators against the MCF-7 breast cancer cell line,^40^ with results presented in Table 3. It was found that derivatives 8g and 8h had significantly higher levels of caspase-3 protein (695 ± 5 and 650 ± 5 pg mL^−1^, respectively) compared to the standard substance staurosporine (503 ± 4 pg mL^−1^). The compounds 8g and 8h increased the concentration of caspase-3 protein in the MCF-7 cancer cell line, showing levels that were 11 and 10 times higher than the untreated control and higher than staurosporine. These data indicate that apoptosis may play a role in the antiproliferative effects of the investigated compounds, possibly due to caspase-3 upregulation.

**Table 3:** Caspase-3 activation of compounds 8g and 8h against MCF-7 cancer cell line

Compound number	Caspase-3	
	Conc. (pg mL^−1^)	Fold change
8g	695 ± 5	11
8h	650 ± 5	10
Staurosporine	503 ± 4	8
Control	63	1

##### 3.2.5.2. Assay for caspase-8, Bax and Bcl-2 levels

We further examined the effects of compounds 8g and 8h on caspase-8, Bax, and the anti-apoptotic Bcl2 levels in the MCF-7 cancer cell line, using staurosporine as a reference.^40^Table 4 displays the findings. The results indicated that 8g and 8h markedly elevated Bax and caspase-8 levels in comparison to staurosporine. Compound 8g had the most overexpression of caspase-8 (2.60 ng mL^−1^), followed by compound 8h (2.10 ng mL^−1^) and staurosporine as a control (1.80 ng mL^−1^). Furthermore, 8g and 8h demonstrated greater Bax induction (315 and 295 pg mL^−1^, respectively) relative to staurosporine (280 pg mL^−1^), exhibiting a 39-fold and 37-fold increase compared to untreated control MCF-7 cancer cells.

**Table 4:** Caspase-8, Bax, and Bcl-2 levels of compounds 8g and 8h in cancer MCF-7 cell line

Compound number	Caspase-8		Bax		Bcl-2	
	Conc. (ng mL^−1^)	Fold change	Conc. (pg mL^−1^)	Fold change	Conc. (ng mL^−1^)	Fold reduction
8g	2.60 ± 0.20	28	315 ± 9	39	0.70	7
8h	2.10 ± 0.20	23	295 ± 9	37	0.90	6
Staurosporine	1.80 ± 0.10	20	280 ± 7	35	1.10	5
Control	0.09	1	8	1	5	1

Finally, compound 8g caused a marketed drop in the concentration of Bcl-2 protein (0.70 ng mL^−1^), followed by compound 8h (0.90 ng mL^−1^) in the MCF-7 cell line compared to staurosporine (1.10 ng mL^−1^). The apoptosis assay showed that compounds 8g and 8h have dual inhibitory effects against EGFR and BRAF^V600E^, showing strong apoptotic antiproliferative effect.

#### 3.2.6. Cell cycle analysis and apoptosis assays

##### 3.2.6.1. Cell cycle analysis

**Fig. 5:**
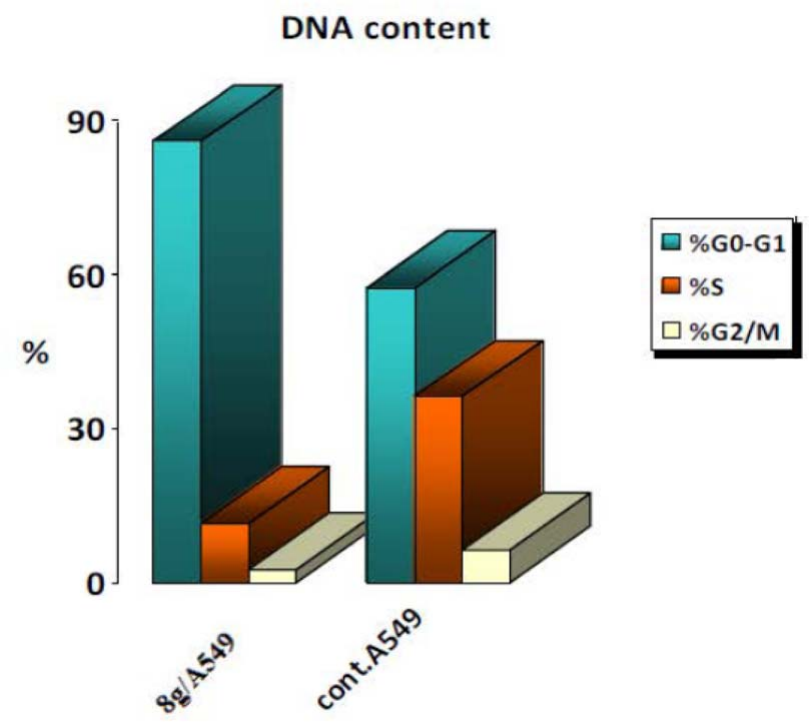
Results for cell cycle analysis of 8g in A-549 cell line.

The effects of 8g on cell cycle progression and apoptosis induction in A-549 cells were examined. A lung cancer (A-549) cell line was subjected to treatment for 24 hours with an IC_50_ concentration of 8g. We stained the cell line with PI/annexin V and performed flow cytometry using a BD FACS Caliber.^41^ The results (Fig. 5) showed that A-549 cells treated with compound 8g had a significant accumulation of 86% in the G0/G1 phase after 24 hours of incubation. This means a cell cycle arrest at the G1 transition.

##### 3.2.6.2. Apoptosis induction assay

**Fig. 6:**
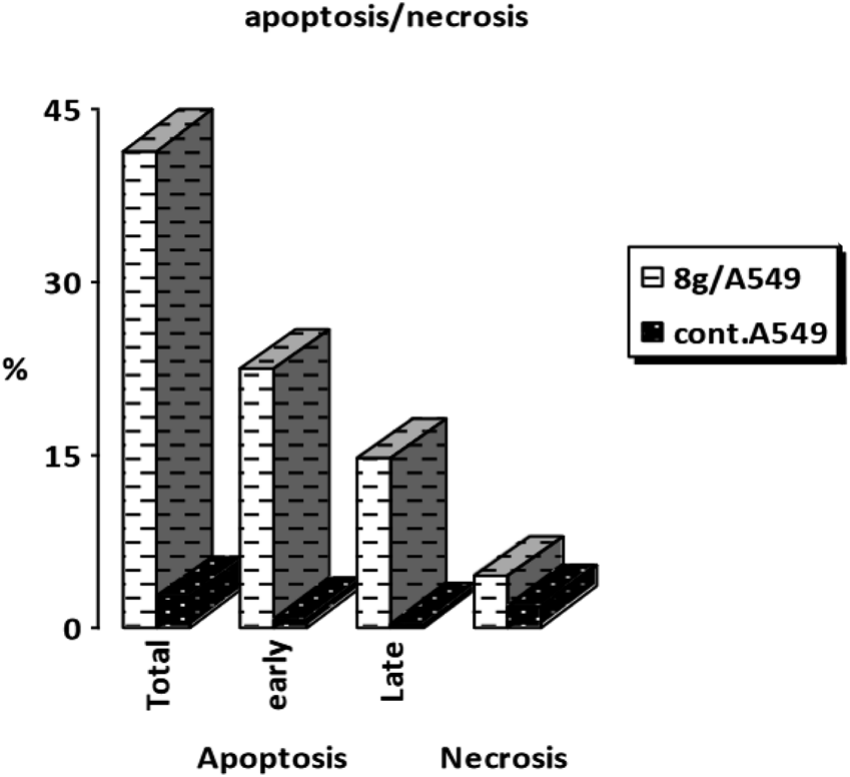
The apoptosis induction results of 8g.

**Fig. 7:**
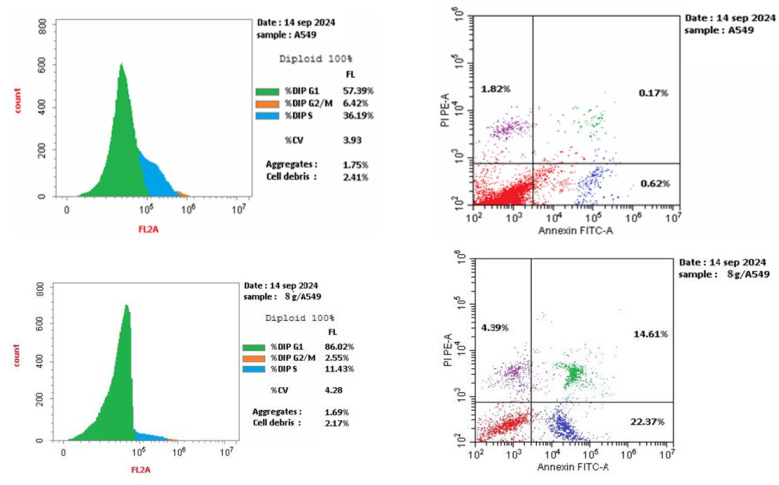
Cell cycle analysis and apoptosis induction results of 8g.

A-549 cells was stained with annexin V/PI, cultured for 24 hours, and analyzed them to examine 8g's capacity to cause apoptosis. Analysis of early and late apoptosis revealed that compound 8g induced significant apoptosis, with a necrosis percentage of 4.39 (Fig. 6 and 7).

### 3.3. *In silico* docking simulation

This study conducted a thorough computer docking analysis to determine the binding relationships between chemicals 8f, 8g, 8h, 8j, and 8l and EGFR-TK. The present study employed the Discovery Studio program to conduct a comprehensive analysis of the interaction mechanism.^55,56^ To improve this analysis, we incorporated the crystallographic structure of the EGFR-erlotinib complex, as documented in the Protein Data Bank (PDB ID: 1M17),^42,57^ to give the study a strong structural base, as well as the crystallographic configuration of EGFR complexed with Erlotinib. To test the docking technique's efficacy, we re-docked the co-crystallized ligand erlotinib into the active site of the EGFR protein. The technique produced an S score of −7.30 kcal mol^−1^, indicating the procedure's precision. A strong hydrogen bond interaction was made between the pyrimidine nitrogen in Erlotinib and the amino acid residue Met769 in the EGFR structure, which made the docking result even more clear. This interaction is crucial for stabilizing the ligand in the active site, underscoring the significance of molecular interactions in the binding process. We docked compounds 8f, 8g, 8h, 8j, and 8l with EGFR kinase to determine the binding affinity of derivatives with the best *in vitro* activity against EGFR, and Table 5 shows their docking scores.

**Table 5:** Docking scores (*S*; kcal mol^−1^) of 8f, 8g, 8h, 8j, and 8l against EGFR and BRAF^V600E^

Compound	EGFR (PDB ID: 1M17)	BRAF^V600E^ (PDB ID: 4RZV)
8f	−6.13	−6.77
8g	−6.66	−7.97
8h	−6.63	−7.23
8j	−6.39	−7.07
8l	−6.3	−6.53
Erlotinib	−7.3	—
Vemurafenib	—	−10.53

**Fig. 8:**
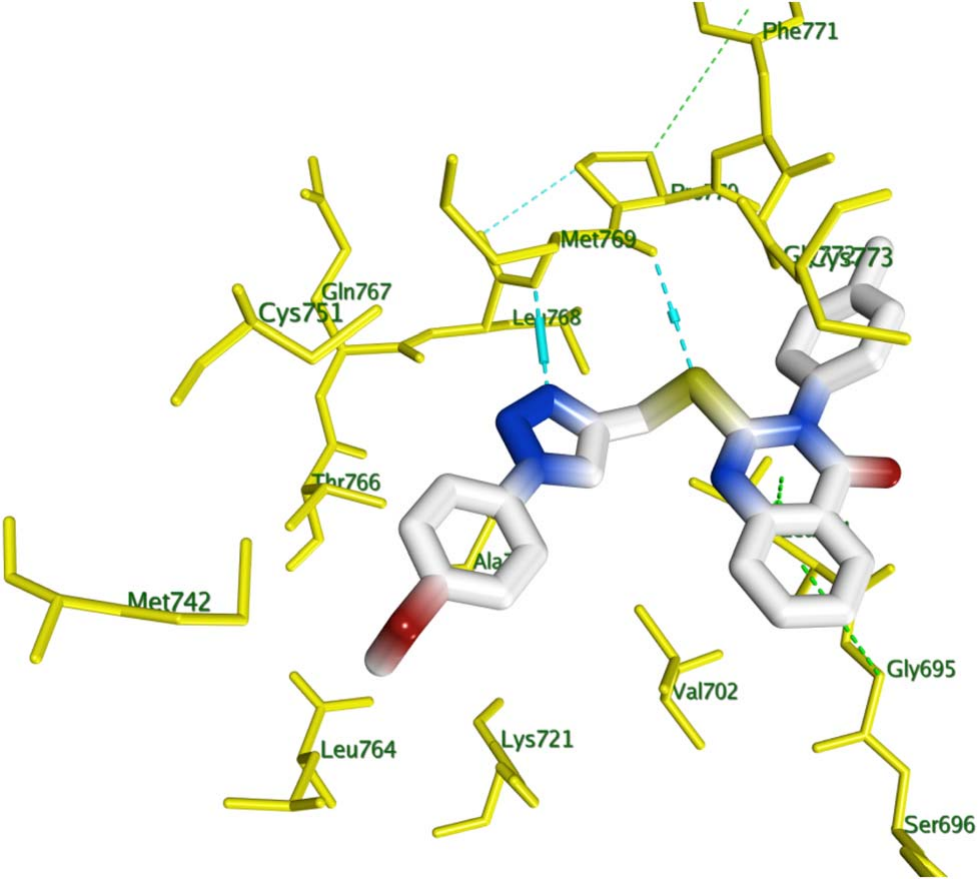
3D closest interactions between active site amino acid residues of EGFR kinase (PDB ID: 1M17) and best docking score compound 8g.

All the tested compounds had a strong and similar ability to bind to erlotinib at the EGFR enzyme's active site. A visual analysis of the optimal docking pose was conducted to determine potential interactions between the test compounds and the amino acid residues constituting the active site. Fig. 8 illustrates how two H-bond interactions with MET 769 and a pi–H interaction with LEU 694 stabilized the structure of compound 8g, which had the highest docking score among its congeners, within EGFR binding site.

**Fig. 9:**
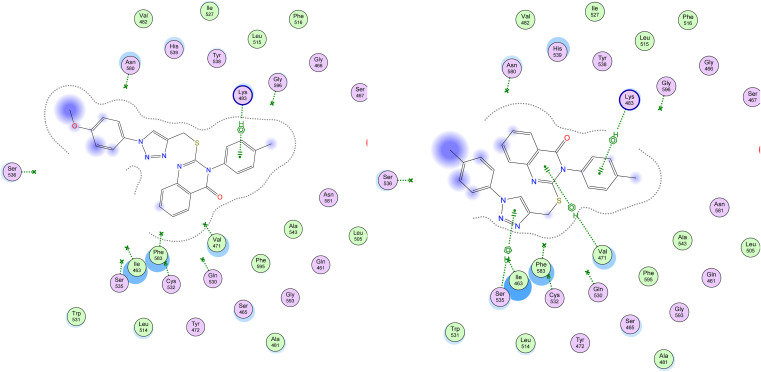
Depicted 2D figures of binding interactions of compounds 8g and 8h within active site of BRAF^V600E^ (PDB ID: 4RZV) showing pi–H bonds as green-dotted lines with LYS 483, VAL 471, and SER 535.

The study's most effective antiproliferative hybrids, 8g and 8h, was looked at in more detail using *in silico* docking to find out how it binds to the active site of BRAF^V600E^. This exploratory method utilized the crystal structure of BRAF^V600E^ in complex with Vemurafenib (PDB ID: 4RZV) as a reference point.^43,58^ Compounds 8g and 8h showed the highest binding affinity (*S* = −7.97 and −7.23 kcal mol; respectively) among their test congeners, and visual inspection of their docking poses revealed a number of pi-H interactions *via* –*N-p*-tolyl moiety and quinazoline ring with LYS 483 and VAL 471, in addition to triazole ring with SER 535, as shown in Fig. 9.

In summary, these docking experiments provide good insights into the potential inhibitory activity of these new quinazolines against EGFR and BRAF kinases, and their high binding affinity within test crystal structures suggesting that they can effectively interact with crucial amino acid residues of both kinases.

## 4. Conclusion

In search of novel antiproliferative scaffolds, we designed and synthesized twenty new 1,2,3-oxadiazole/quinazoline-4-one hybrids (8a–t) that inhibit both EGFR and BRAF^V600E^. The novel hybrids showed promising antiproliferative activities. We investigated the inhibitory effects of derivatives 8f, 8g, 8h, 8j, and 8l on EGFR and BRAF^V600E^. *In vitro* experiments revealed that compounds 8g, 8h, and 8l are effective cancer-fighting medicines that inhibit both EGFR and BRAF^V600E^. Additionally, compounds 8g and 8h may have apoptotic action because they activated caspase 3, 8, and Bax while inhibiting Bcl2. Cell cycle analysis and apoptosis induction assays on 8g revealed cell cycle arrest in the G1 phase. Docking experiments revealed structural insights, with compound 8g exhibiting strong binding affinities for both EGFR and BRAF^V600E^, as indicated by its high docking scores. Upon optimization, these novel hybrids may have the potential to act as anticancer agents.

## Data availability

Samples of compounds 8a–t are available from the authors.

## Conflicts of interest

The author reported no potential conflicts of interest(s).

## Supplementary Material

RA-014-D4RA06694D-s001

## References

[cit1] Letai A. (2017). Annu. Rev. Cancer Biol..

[cit2] Lee E. J. (2016). Cystogenesis.

[cit3] Zhu S., Zhang T., Zheng L., Liu H., Song W., Liu D., Li Z., Pan C.-x. (2021). J. Hematol. Oncol..

[cit4] Lu N., Wu J., Tian M., Zhang S., Li Z., Shi L. (2024). Eur. J. Med. Chem..

[cit5] Sudhesh Dev S., Zainal Abidin S. A., Farghadani R., Othman I., Naidu R. (2021). Front. Pharmacol.

[cit6] Esteban-Villarrubia J., Soto-Castillo J. J., Pozas J., San Román-Gil M., Orejana-Martín I., Torres-Jiménez J., Carrato A., Alonso-Gordoa T., Molina-Cerrillo J. (2020). Int. J. Mol. Sci..

[cit7] Al-Wahaibi L. H., Elshamsy A. M., Ali T. F., Youssif B. G., Bräse S., Abdel-Aziz M., El-Koussi N. A. (2024). ACS Omega.

[cit8] Al-Wahaibi L. H., Mohammed A. F., Abdel Rahman F. E.-Z. S., Abdelrahman M. H., Gu X., Trembleau L., Youssif B. G. (2023). J. Enzyme Inhib. Med. Chem..

[cit9] Notarangelo T., Sisinni L., Condelli V., Landriscina M. (2017). Cancer Cell Int..

[cit10] Mondaca S., Lacouture M., Hersch J., Yaeger R. (2018). JCO Precis. Oncol..

[cit11] Okaniwa M., Hirose M., Imada T., Ohashi T., Hayashi Y., Miyazaki T., Arita T., Yabuki M., Kakoi K., Kato J. (2012). J. Med. Chem..

[cit12] Zhang Q., Diao Y., Wang F., Fu Y., Tang F., You Q., Zhou H. (2013). MedChemComm.

[cit13] Laxmikeshav K., Kumari P., Shankaraiah N. (2022). Med. Res. Rev..

[cit14] Banik B. K. and Banerjee B., Heterocyclic Anticancer Agents, Walter de Gruyter GmbH & Co KG, 2022

[cit15] Moradi M., Mousavi A., Emamgholipour Z., Giovannini J., Moghimi S., Peytam F., Honarmand A., Bach S., Foroumadi A. (2023). Eur. J. Med. Chem..

[cit16] Sharma S., Sharma K., Pathak S., Kumar M., Sharma P. K. (2020). Open Med. Chem. J..

[cit17] Nepali K., Sharma S., Ojha R., Dhar K. L. (2013). Med. Chem. Res..

[cit18] Panwar V. , Mukherji K., Ghate M., Jindal D. K. and Kumar D., in Biomedical Translational Research: Drug Design and Discovery, Springer, 2022, pp. 387–399

[cit19] Abdel-Mohsen H. T., Anwar M. M., Ahmed N. S., Abd El-Karim S. S., Abdelwahed S. H. (2024). Molecules.

[cit20] Patel R. V., Mistry B. M., Gujarati A. V., Patel A. B., Patel D. K. (2023). ChemistrySelect.

[cit21] Kolb H. C., Finn M., Sharpless K. B. (2001). Angew. Chem., Int. Ed..

[cit22] Akter M., Rupa K., Anbarasan P. (2022). Chem. Rev..

[cit23] Wang Z. Y., Li J., Wang N., Liu H., Wang K. K. (2023). Asian J. Org. Chem..

[cit24] El-Sheref E. M., Bräse S., Tawfeek H. N., Alasmary F. A., Youssif B. G. (2023). Int. J. Mol. Sci..

[cit25] El-Sheref E. M., Elbastawesy M. A., Brown A. B., Shawky A. M., Gomaa H. A., Bräse S., Youssif B. G. (2021). Molecules.

[cit26] Mahmoud M. A., Mohammed A. F., Salem O. I., Almutairi T. M., Bräse S., Youssif B. G. (2024). J. Enzyme Inhib. Med. Chem..

[cit27] Mohamed A. M., Abou-Ghadir O. M., Mostafa Y. A., Dahlous K. A., Bräse S., Youssif B. G. (2024). Front. Chem..

[cit28] Kucuksayan E., Ozben T. (2017). Curr. Top. Med. Chem..

[cit29] Abou-Zied H. A., Beshr E. A., Gomaa H. A., Mostafa Y. A., Youssif B. G., Hayallah A. M., Abdel-Aziz M. (2023). Arch. Pharm..

[cit30] Alshammari M. B., Aly A. A., Youssif B. G., Bräse S., Ahmad A., Brown A. B., Ibrahim M. A., Mohamed A. H. (2022). Front. Chem..

[cit31] Al-Wahaibi L. H., Abou-Zied H. A., Beshr E. A., Youssif B. G., Hayallah A. M., Abdel-Aziz M. (2023). Int. J. Mol. Sci..

[cit32] Al-Wahaibi L. H., El-Sheref E. M., Hammouda M. M., Youssif B. G. (2023). Pharmaceuticals.

[cit33] Al-Wahaibi L. H., Gouda A. M., Abou-Ghadir O. F., Salem O. I., Ali A. T., Farghaly H. S., Abdelrahman M. H., Trembleau L., Abdu-Allah H. H., Youssif B. G. (2020). Bioorg. Chem..

[cit34] Al-Wahaibi L. H., Hisham M., Abou-Zied H. A., Hassan H. A., Youssif B. G., Bräse S., Hayallah A. M., Abdel-Aziz M. (2023). Pharmaceuticals.

[cit35] Al-Wahaibi L. H., Mahmoud M. A., Mostafa Y. A., Raslan A. E., Youssif B. G. (2023). J. Enzyme Inhib. Med. Chem..

[cit36] Mekheimer R. A., Allam S. M., Al-Sheikh M. A., Moustafa M. S., Al-Mousawi S. M., Mostafa Y. A., Youssif B. G., Gomaa H. A., Hayallah A. M., Abdelaziz M. (2022). Bioorg. Chem..

[cit37] Hisham M., Hassan H. A., Gomaa H. A., Youssif B. G., Hayallah A. M., Abdel-Aziz M. (2022). J. Mol. Struct..

[cit38] El-Kalyoubi S. A., Gomaa H. A., Abdelhafez E. M., Ramadan M., Agili F., Youssif B. G. (2023). Pharmaceuticals.

[cit39] Al-Wahaibi L. H., Mohammed A. F., Abdelrahman M. H., Trembleau L., Youssif B. G. (2023). Molecules.

[cit40] Youssif B. G., Mohamed A. M., Osman E. E. A., Abou-Ghadir O. F., Elnaggar D. H., Abdelrahman M. H., Treamblu L., Gomaa H. A. (2019). Eur. J. Med. Chem..

[cit41] El-Sherief H. A., Youssif B. G., Bukhari S. N. A., Abdelazeem A. H., Abdel-Aziz M., Abdel-Rahman H. M. (2018). Eur. J. Med. Chem..

[cit42] Bhat M. A., Tüzün B., Alsaif N. A., Khan A. A., Naglah A. M. (2022). J. Mol. Struct..

[cit43] Singh A. K., Kumar A., Thareja S., Kumar P. (2023). Anti-Cancer Agents Med. Chem..

[cit44] ElZahabi H. S., Nafie M. S., Osman D., Elghazawy N. H., Soliman D. H., El-Helby A. A. H., Arafa R. K., of E. J. (2021). Med. Chem..

[cit45] Moussa G., Alaaeddine R., Alaeddine L. M., Nassra R., Belal A. S., Ismail A., El-Yazbi A. F., Abdel-Ghany Y. S., Hazzaa A. (2018). Eur. J. Med. Chem..

[cit46] Mangione M. I., Spanevello R. A., Anzardi M. (2017). RSC Adv..

[cit47] El-Sherief H. A., Youssif B. G., Abdelazeem A. H., Abdel-Aziz M., Abdel-Rahman H. M. (2019). Anti-Cancer Agents Med. Chem..

[cit48] Borude A. S., Deshmukh S. R., Tiwari S. V., Kumar S. H., Thopate S. R. (2024). Eur. J. Med. Chem..

[cit49] Elmore S. (2007). Toxicol. Pathol..

[cit50] D'arcy M. S. (2019). Cell Biol. Int..

[cit51] Foight G. W. , Determinants of protein-peptide interaction specificity in the Bcl-2 and TRAF families, Massachusetts Institute of Technology, 2015

[cit52] Chen Y.-F., Lee A.-S., Chen W.-Y., Lin C.-H., Kuo C.-L., Chung J.-G. (2020). Am. J. Chin. Med..

[cit53] Naumova N., Šachl R. (2020). Membranes.

[cit54] Ponder K. G., Boise L. H. (2019). Cell Death Discovery.

[cit55] Ibrahim T. S., Bokhtia R. M., Al-Mahmoudy A. M., Taher E. S., AlAwadh M. A., Elagawany M., Abdel-Aal E. H., Panda S., Gouda A. M., Asfour H. Z. (2020). Bioorg. Chem..

[cit56] Shaykoon M. S., Marzouk A. A., Soltan O. M., Wanas A. S., Radwan M. M., Gouda A. M., Youssif B. G., Abdel-Aziz M. (2020). Bioorg. Chem..

[cit57] Jakhmola V., Parashar T., Ghildiyal P., Ansori A., Sharma R. K., Rao N. R., Kalra K., Singh N., Nainwal N., Singh R. K. (2022). Pharmacogn. J..

[cit58] Kircher T., Pantsar T., Oder A., von Kries J. P., Juchum M., Pfaffenrot B., Kloevekorn P., Albrecht W., Selig R., Laufer S. (2021). Eur. J. Med. Chem..

