# Supplementary material for: Design, synthesis, and antiproliferative activity of new 1,2,3-triazole/quinazoline-4-one hybrids as dual EGFR/BRAFV600E inhibitors
Source: RSC Adv. 2024 Dec 5;14(52):38403–15. doi: 10.1039/d4ra06694d (PMC11618052; doi:10.1039/d4ra06694d)
Supplement: RA-014-D4RA06694D-s001 [file RA-014-D4RA06694D-s001.pdf]

Supplementary Information (SI) for RSC Advances.
This journal is © The Royal Society of Chemistry 2024

Supplementary Data

Design, synthesis, and antiproliferative activity of new 1,2,3-Triazole/Quinazoline-4-one hybrids as dual EGFR/BRAF^{V600E} inhibitors

Amira M. Mohamed[1], Ola M. F. Abou-Ghadir[1], Yaser A. Mostafa[1], Kholood A. Dahlous[2], Stefan Bräse[3]*, Bahaa G. M. Youssif[1]*

[1]Pharmaceutical Organic Chemistry Department, Faculty of Pharmacy, Assiut University, Assiut 71526, Egypt; [2]Department of Chemistry, College of Science, King Saud University, Riyadh 11451, Saudi Arabia; [3]Institute of Biological and Chemical Systems, IBCS-FMS, Karlsruhe Institute of Technology, 76131 Karlsruhe, Germany

**To whom correspondence should be addressed:*

Bahaa G. M. Youssif, Ph.D. Pharmaceutical Organic Chemistry Department, Faculty of Pharmacy, Assiut University, Assiut 71526, Egypt.

Tel.: (002)-01098294419

E-mail address: bgyoussif2@gmail.com, bahaa.youssif@pharm.aun.edu.eg

Stefan Bräse

Institute of Biological and Chemical Systems, IBCS-FMS, Karlsruhe Institute of Technology, 76131 Karlsruhe, Germany. E-mail: braese@kit.edu

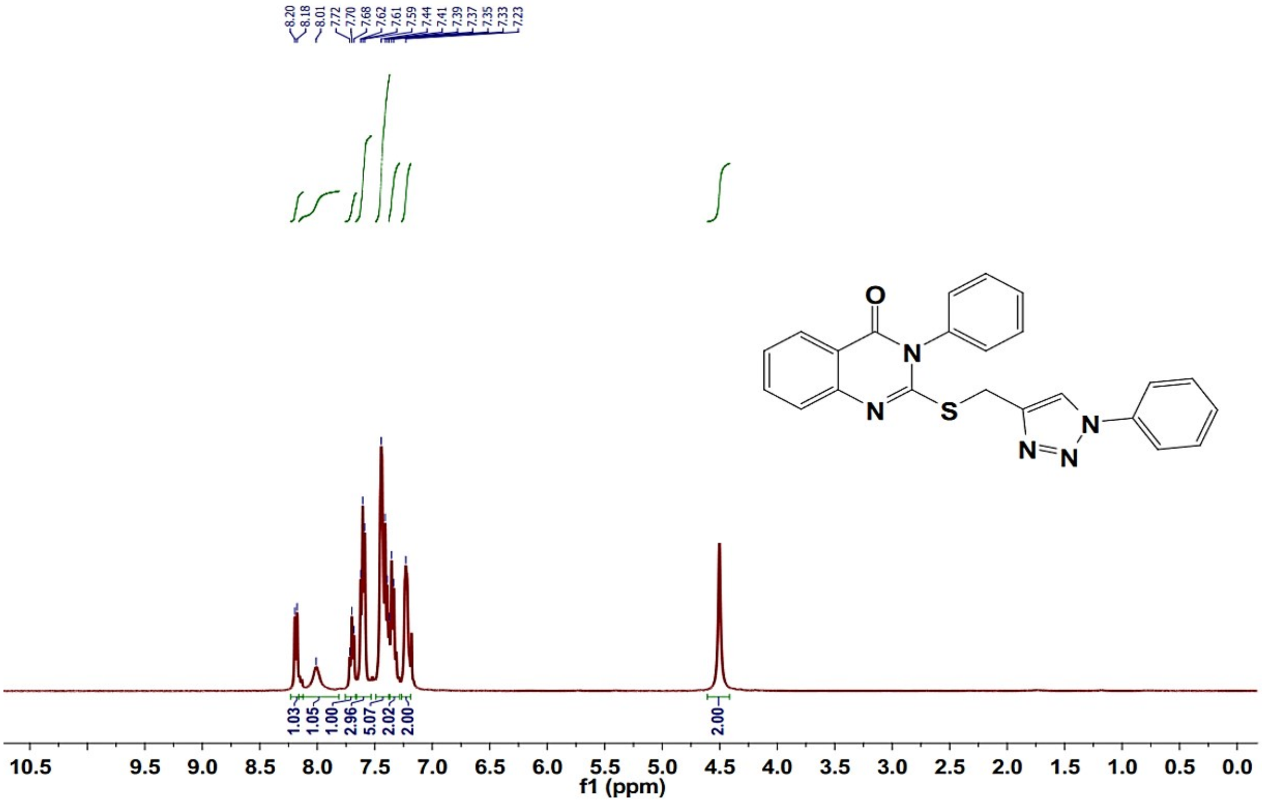

Figure S1: ^{1}H NMR spectrum (400 MHz, $CDCl_3$) of compound **8a**

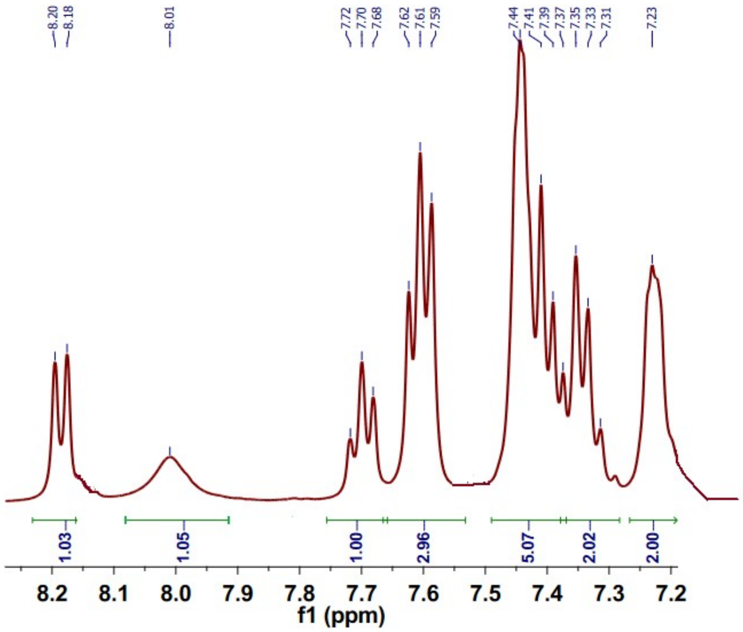

Figure S2: Expanded aromatic region of ^{1}H NMR spectrum (400 MHz, $CDCl_3$) of compound **8a**

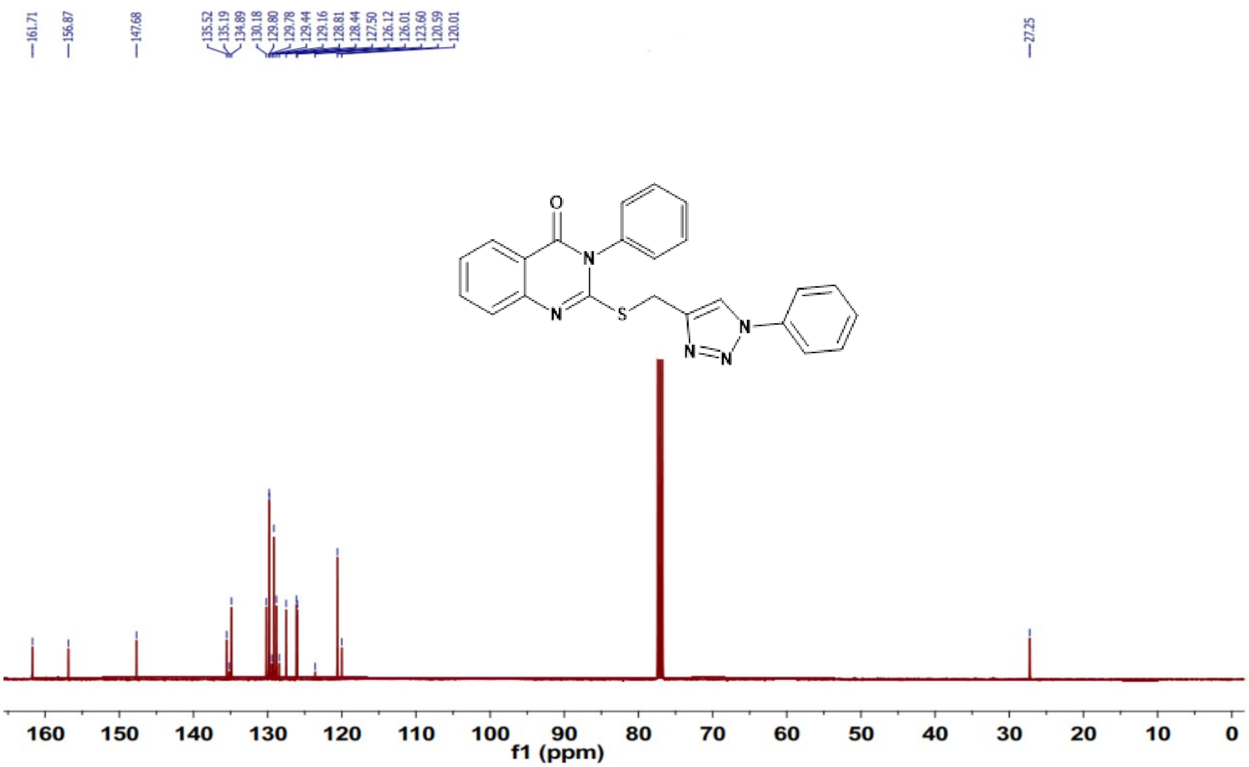

Figure S3: ^{13}C NMR spectrum (100 MHz, $CDCl_3$) of compound **8a**

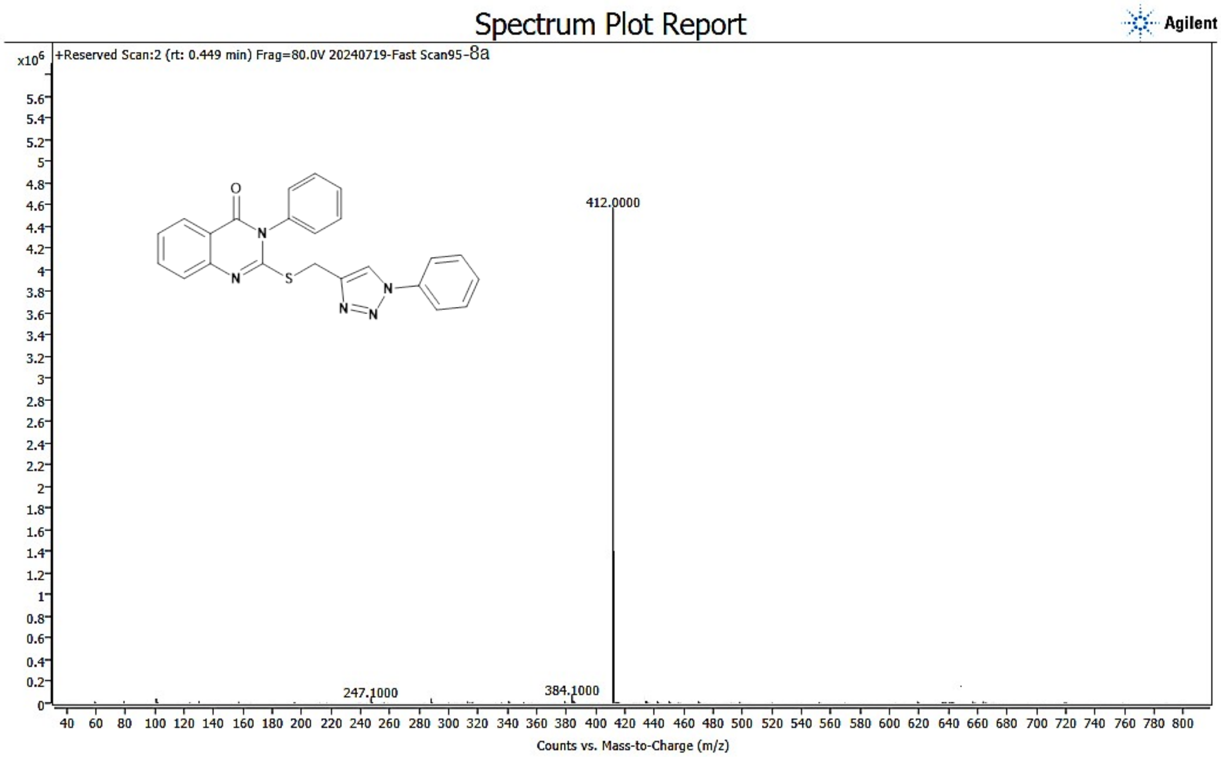

Figure S4: ESI-Mass spectrum of compound **8a**

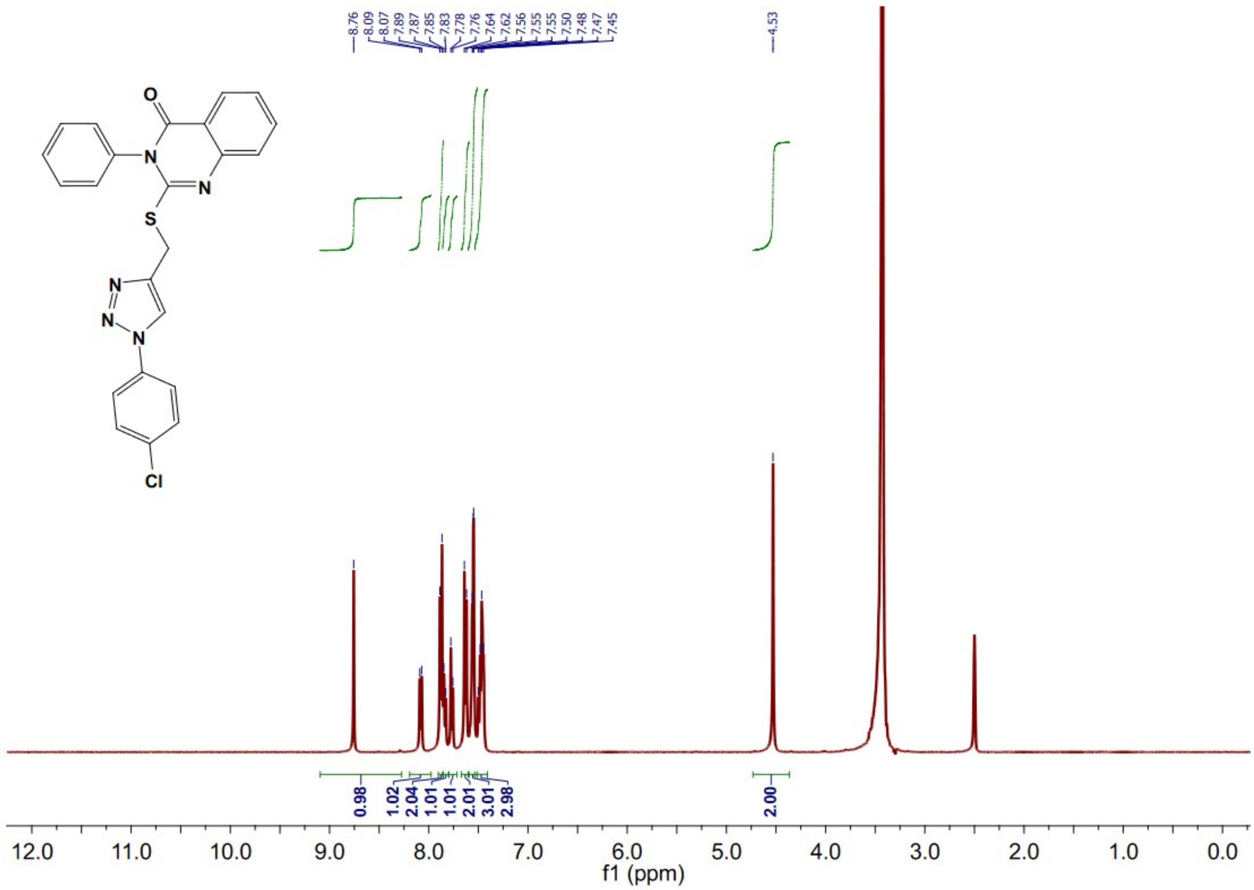

Figure S5: ^{1}H NMR spectrum (400 MHz, DMSO-d_6) of compound **8b**

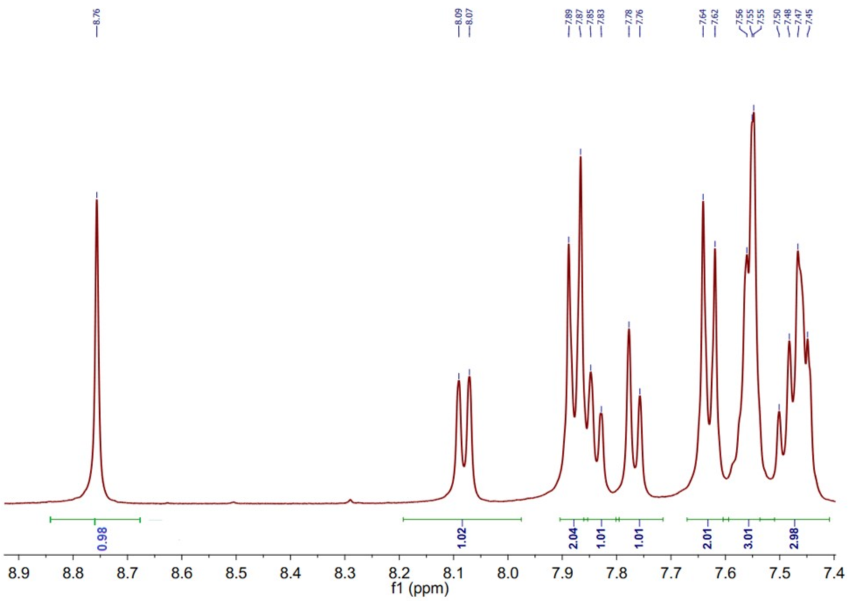

Figure S6: Expanded aromatic region of ^{1}H NMR spectrum (400 MHz, DMSO-d_6) of compound **b**

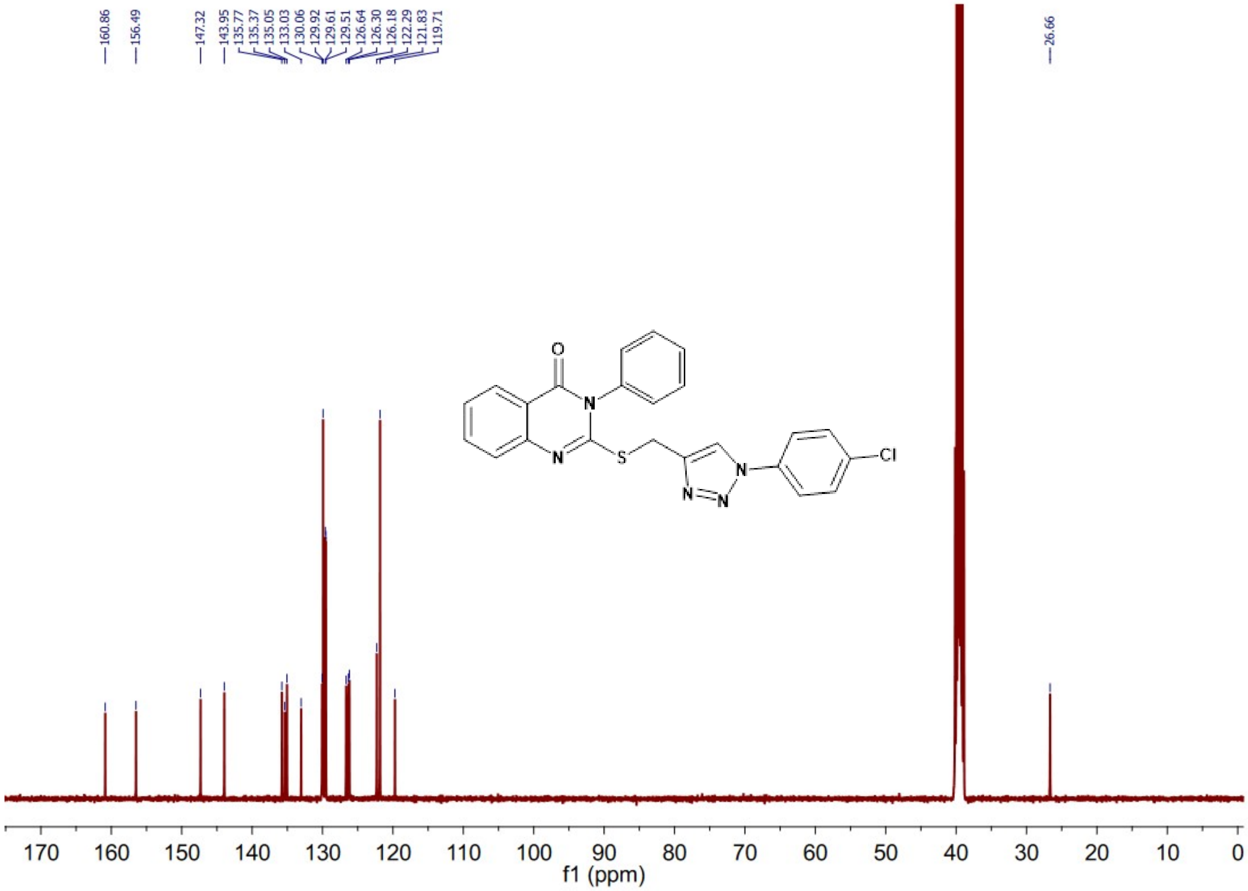

Figure S7: ^{13}C NMR spectrum (100 MHz, DMSO-d_6) of compound **8b**

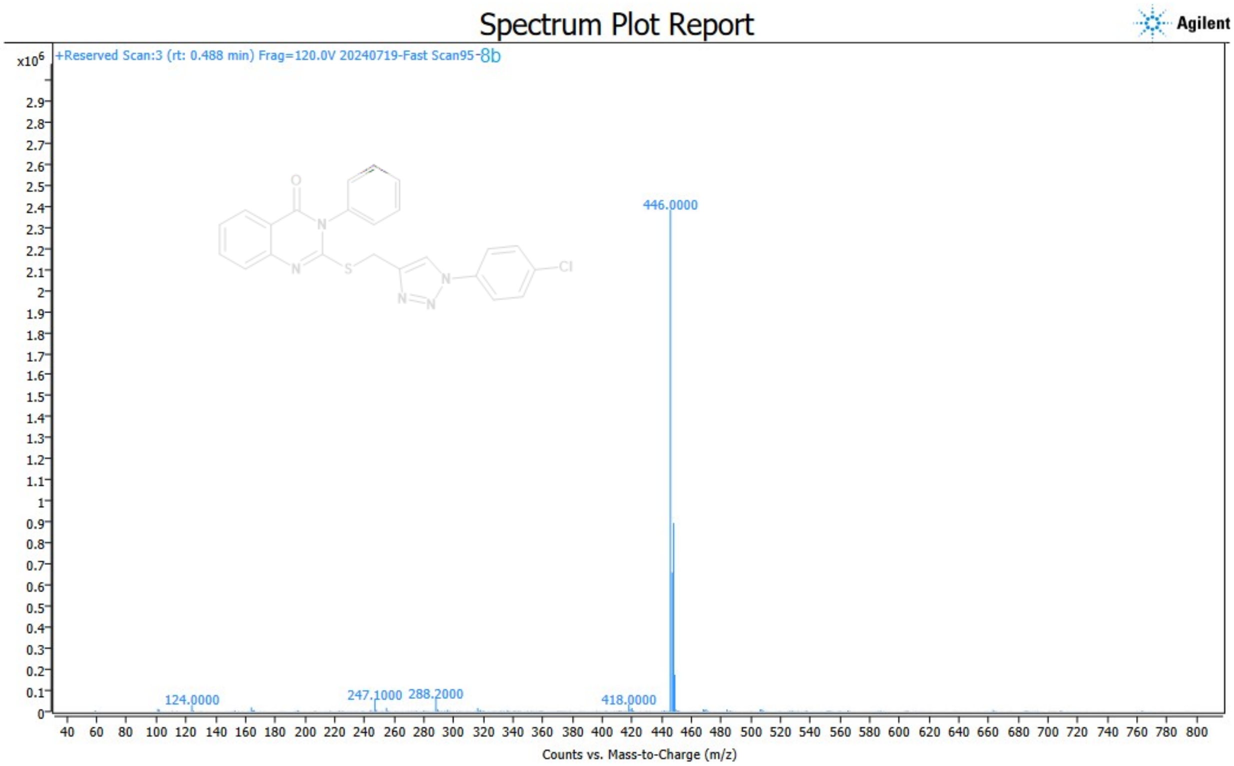

Figure S8: ESI-Mass spectrum of compound **8b**

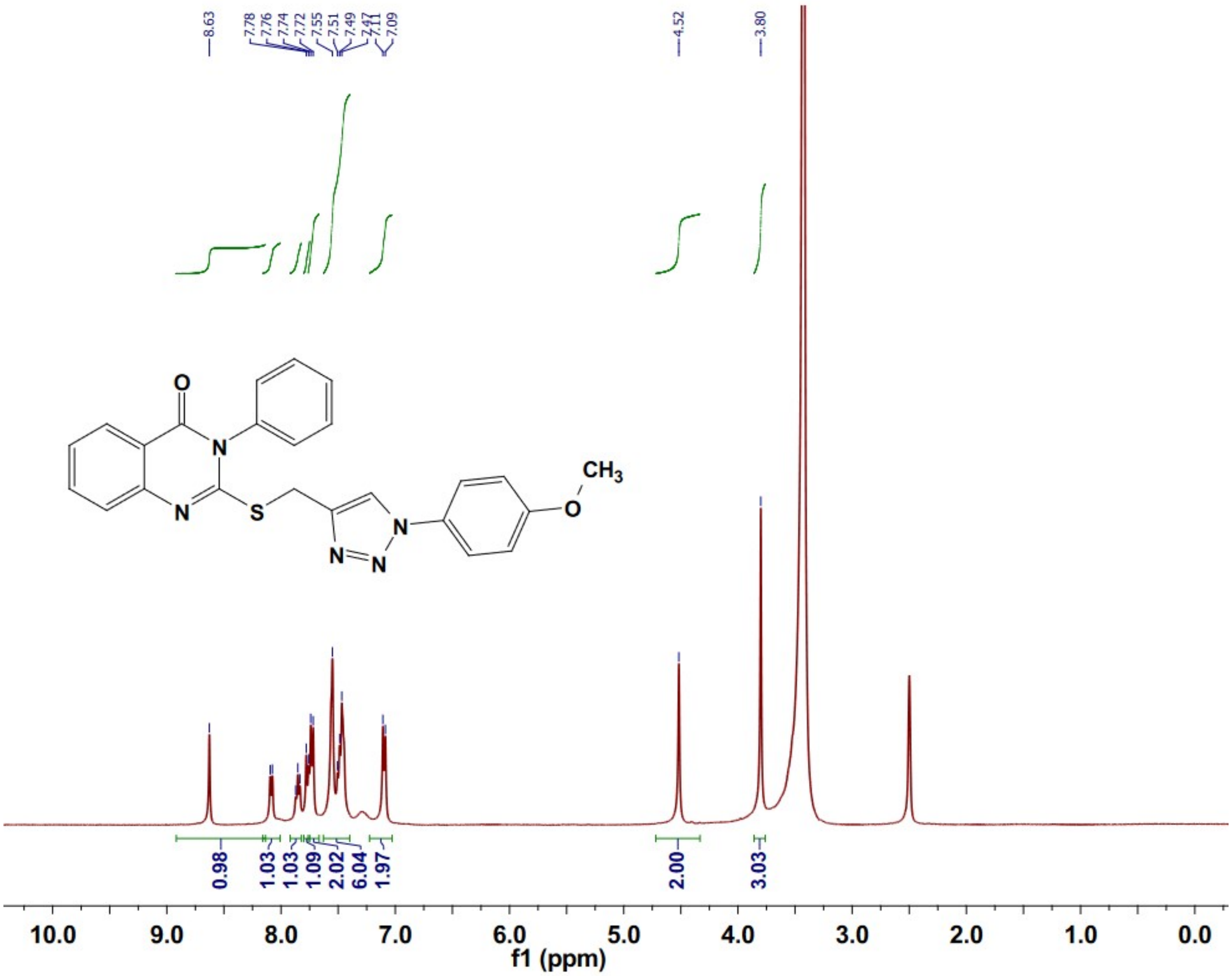

Figure S9: ^{1}H NMR spectrum (400 MHz, DMSO-d_6) of compound **8c**

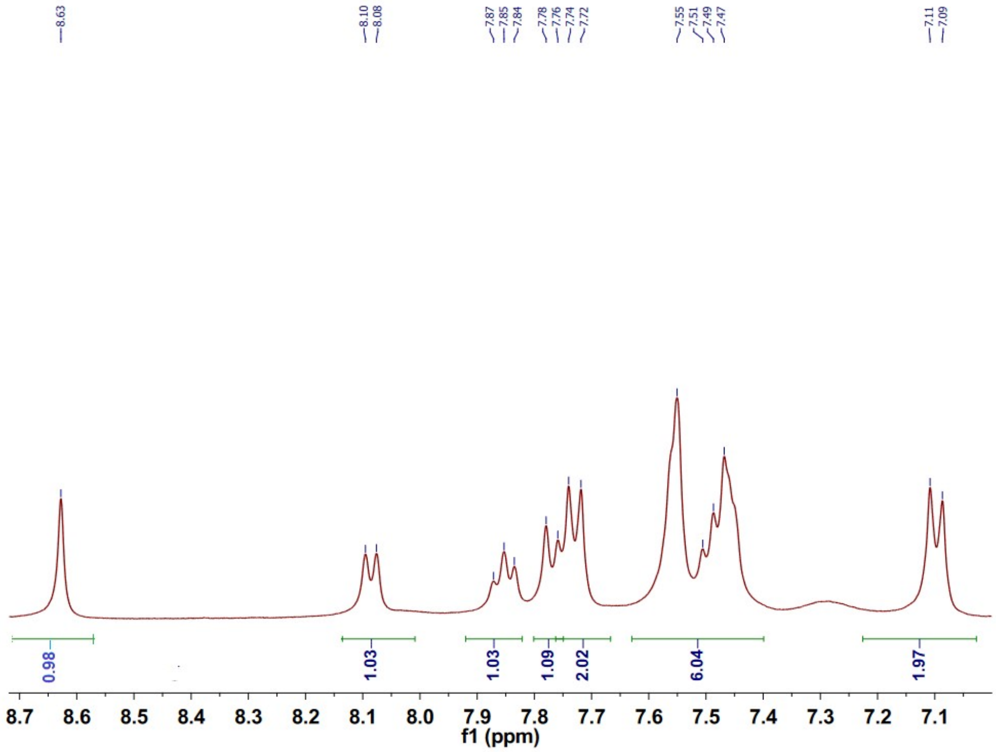

Figure S10: Expanded aromatic region of ^{1}H NMR spectrum (400 MHz, DMSO-d_6) of compound **c**

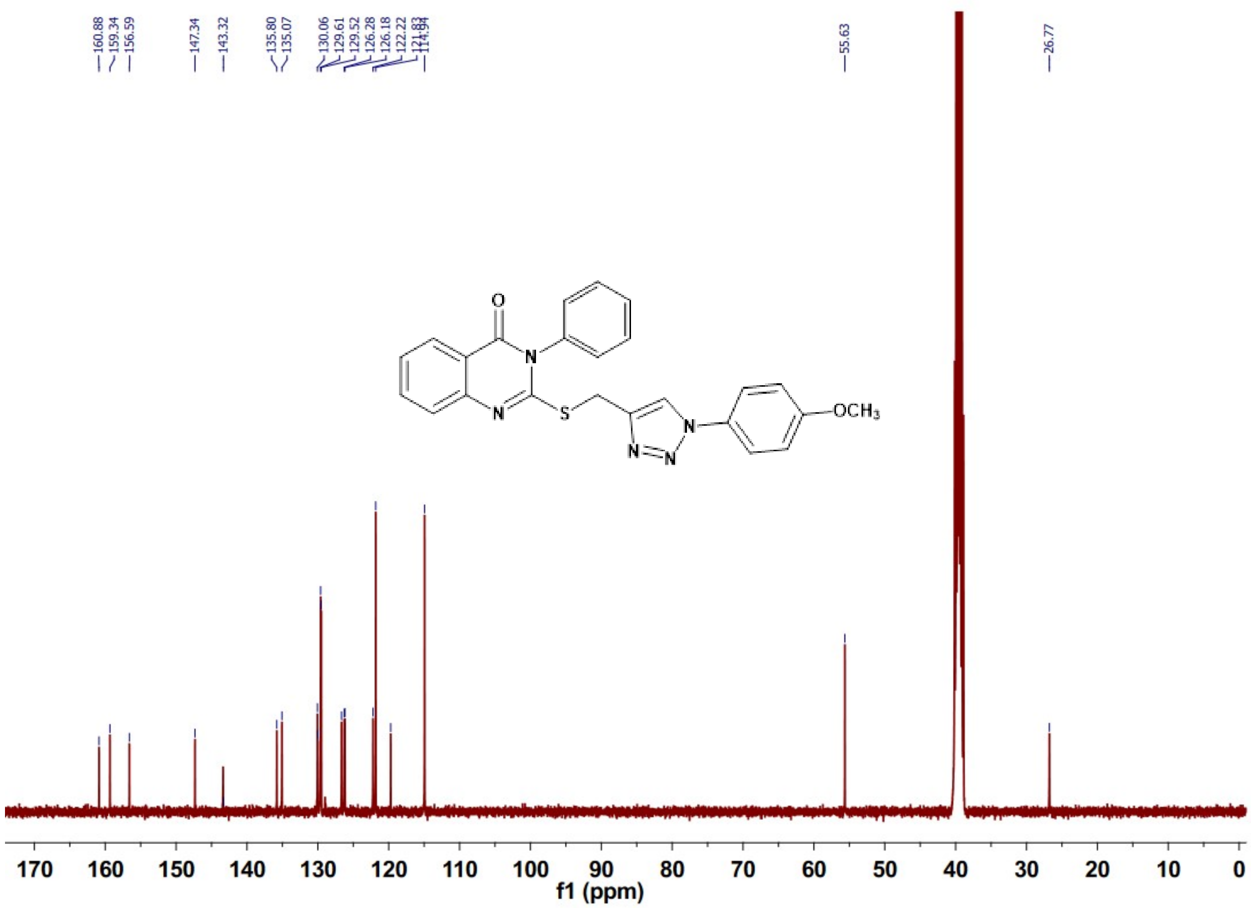

Figure S11: ^{13}C NMR spectrum (100 MHz, DMSO-d_6) of compound **8c**

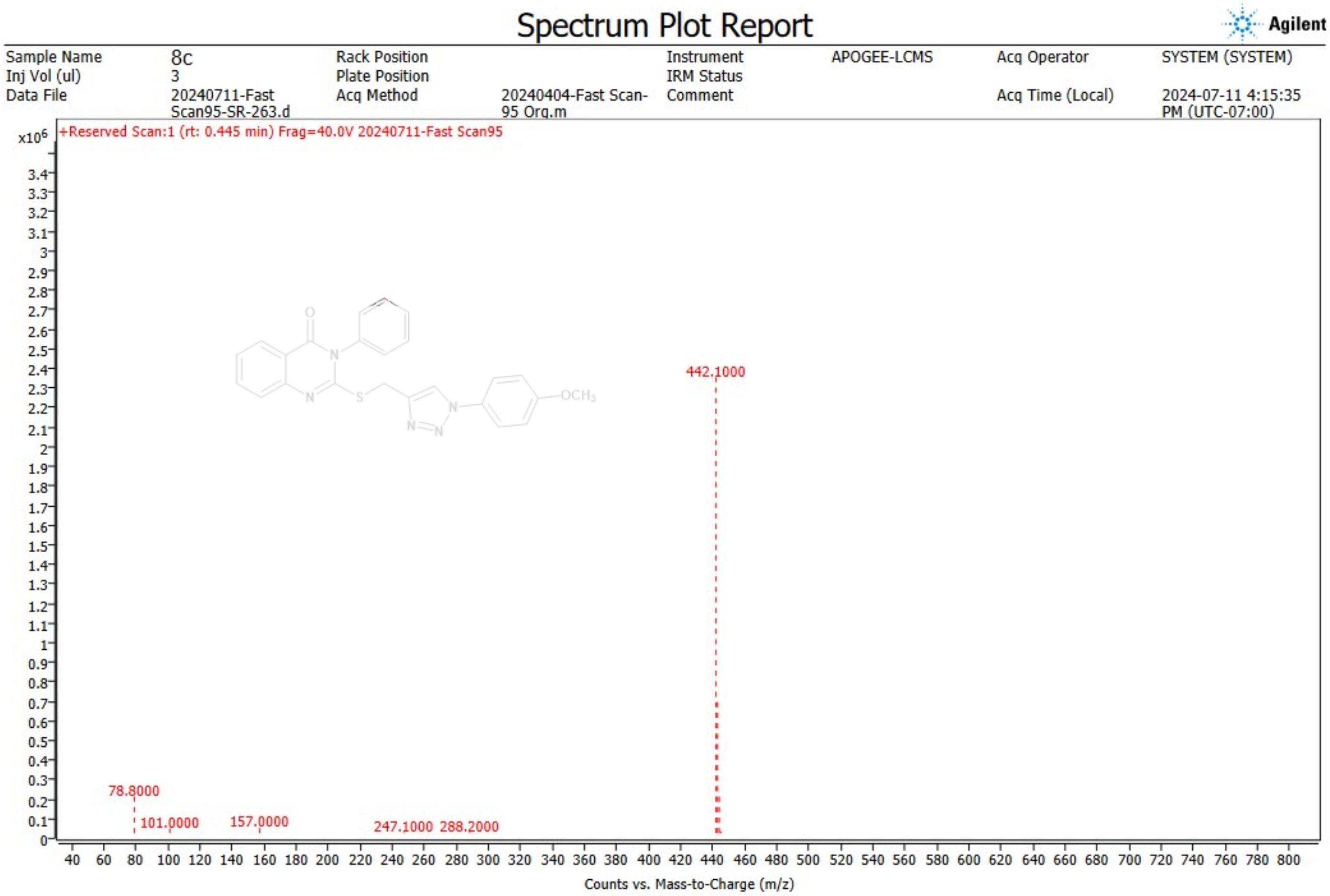

Figure S12: ESI-Mass spectrum of compound **8c**

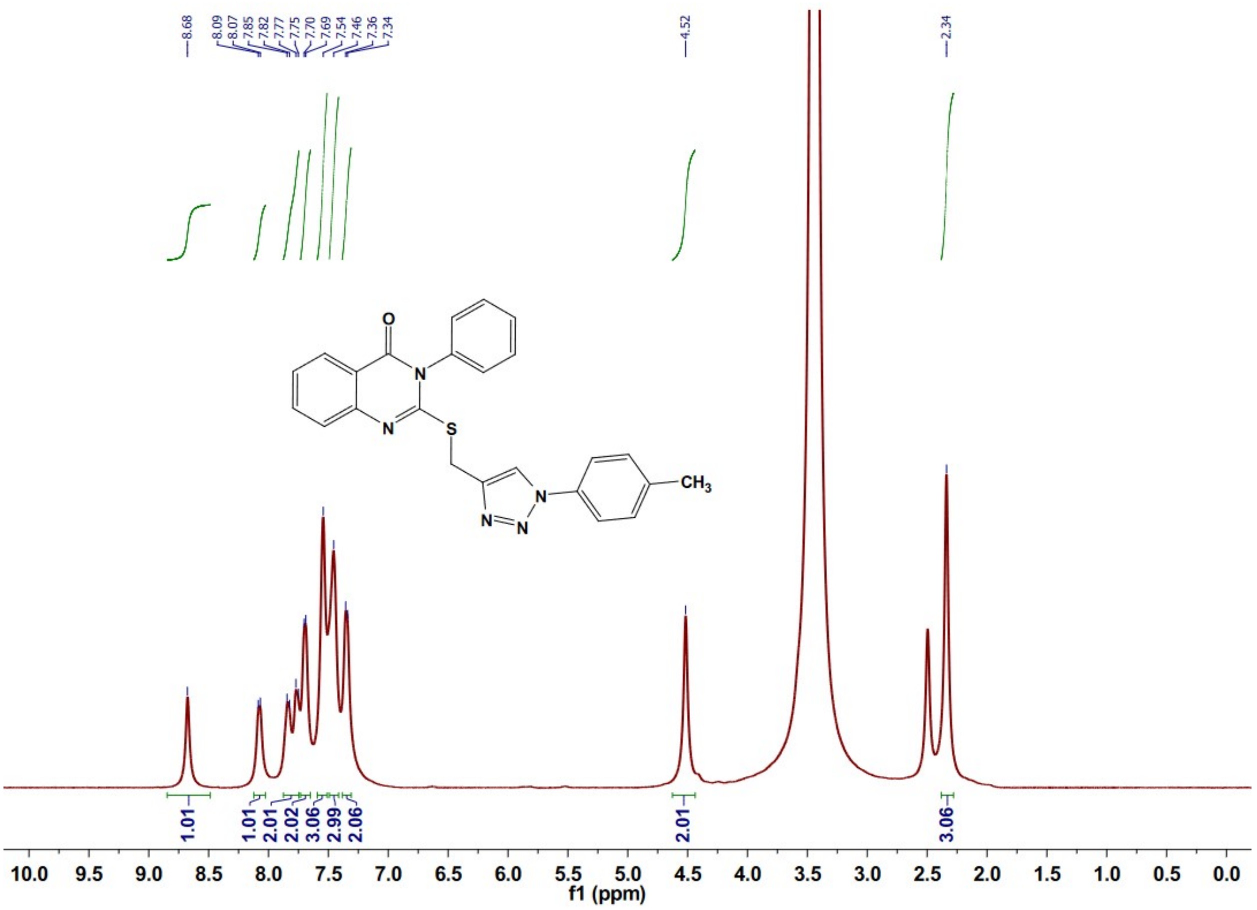

Figure S13: ^{1}H NMR spectrum (400 MHz, DMSO-d_6) of compound **8d**

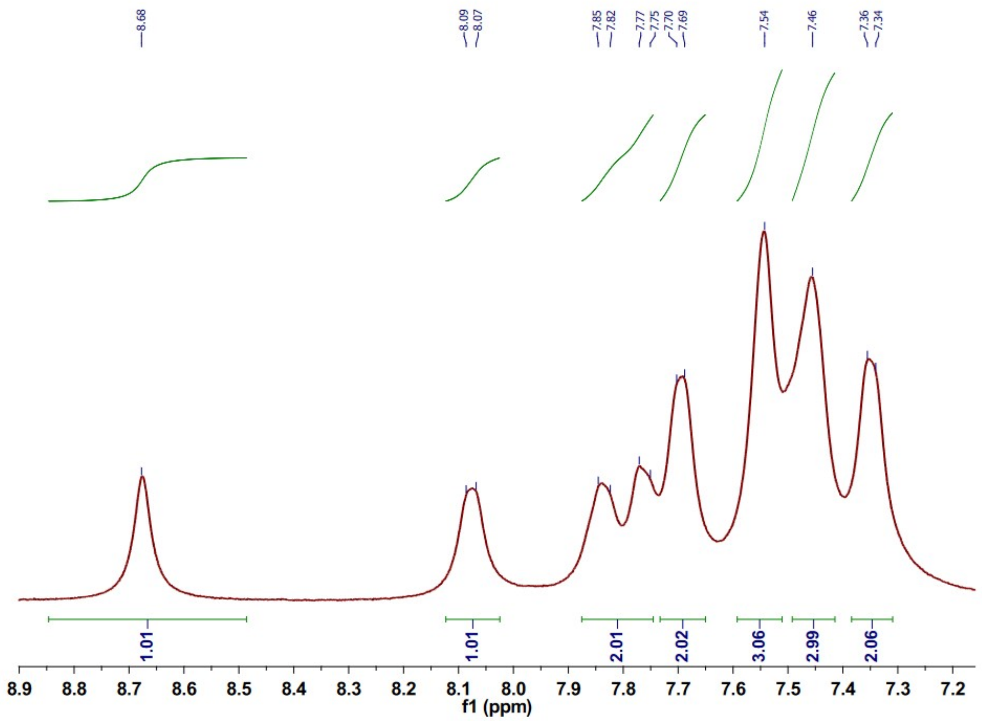

Figure S14: Expanded aromatic region of ^{1}H NMR spectrum (400 MHz, DMSO-d_6) of compound **d**

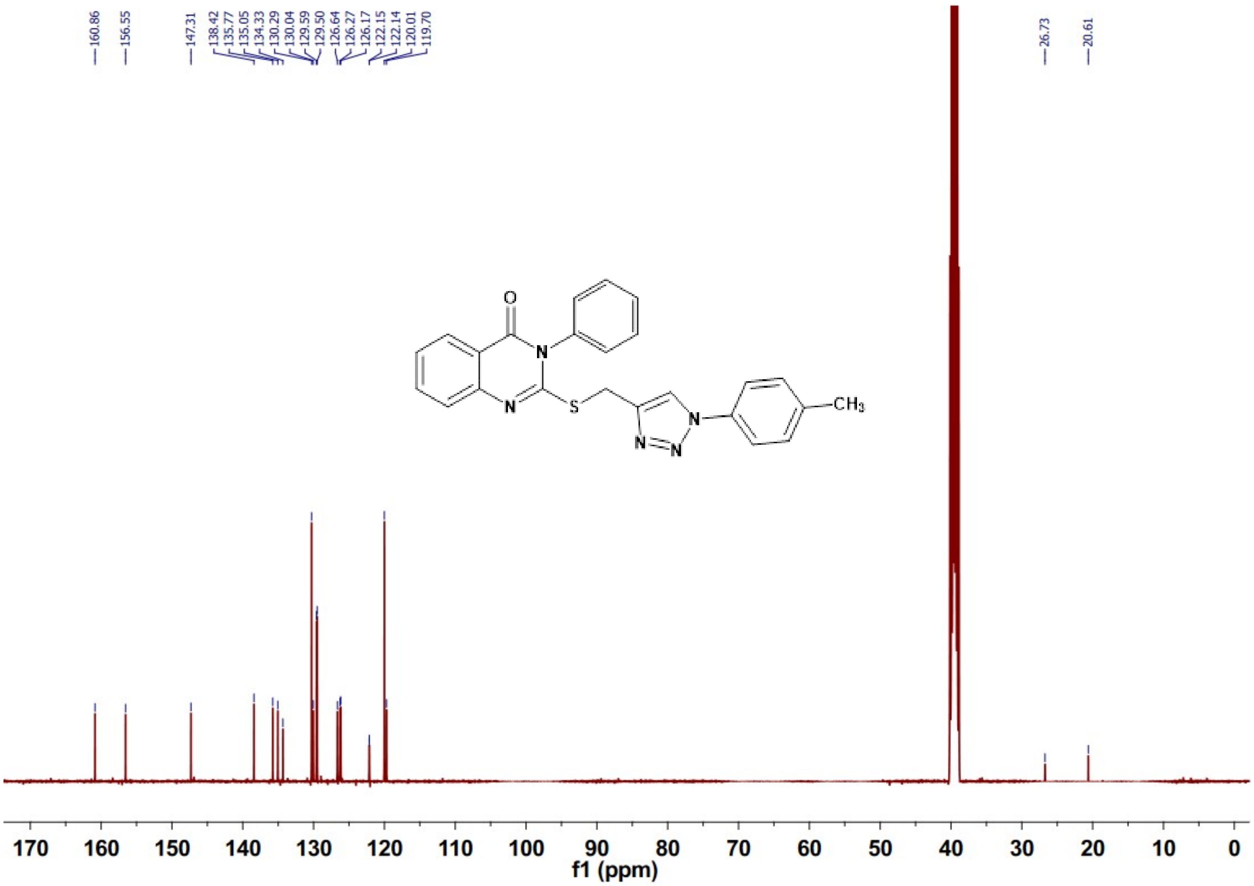

Figure S15: ^{13}C NMR spectrum (100 MHz, DMSO-d_6) of compound **8d**

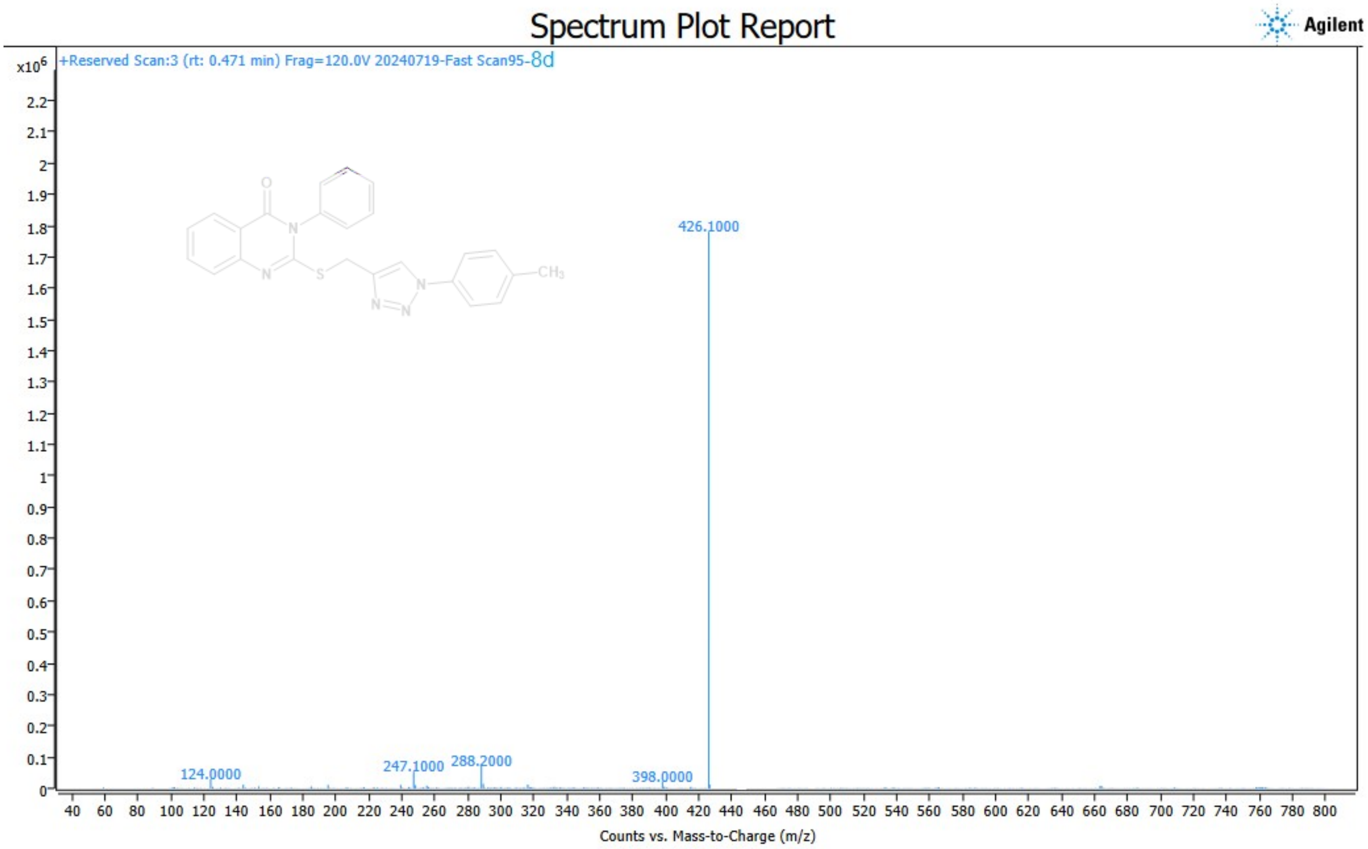

Figure S16: ESI-Mass spectrum of compound **8d**

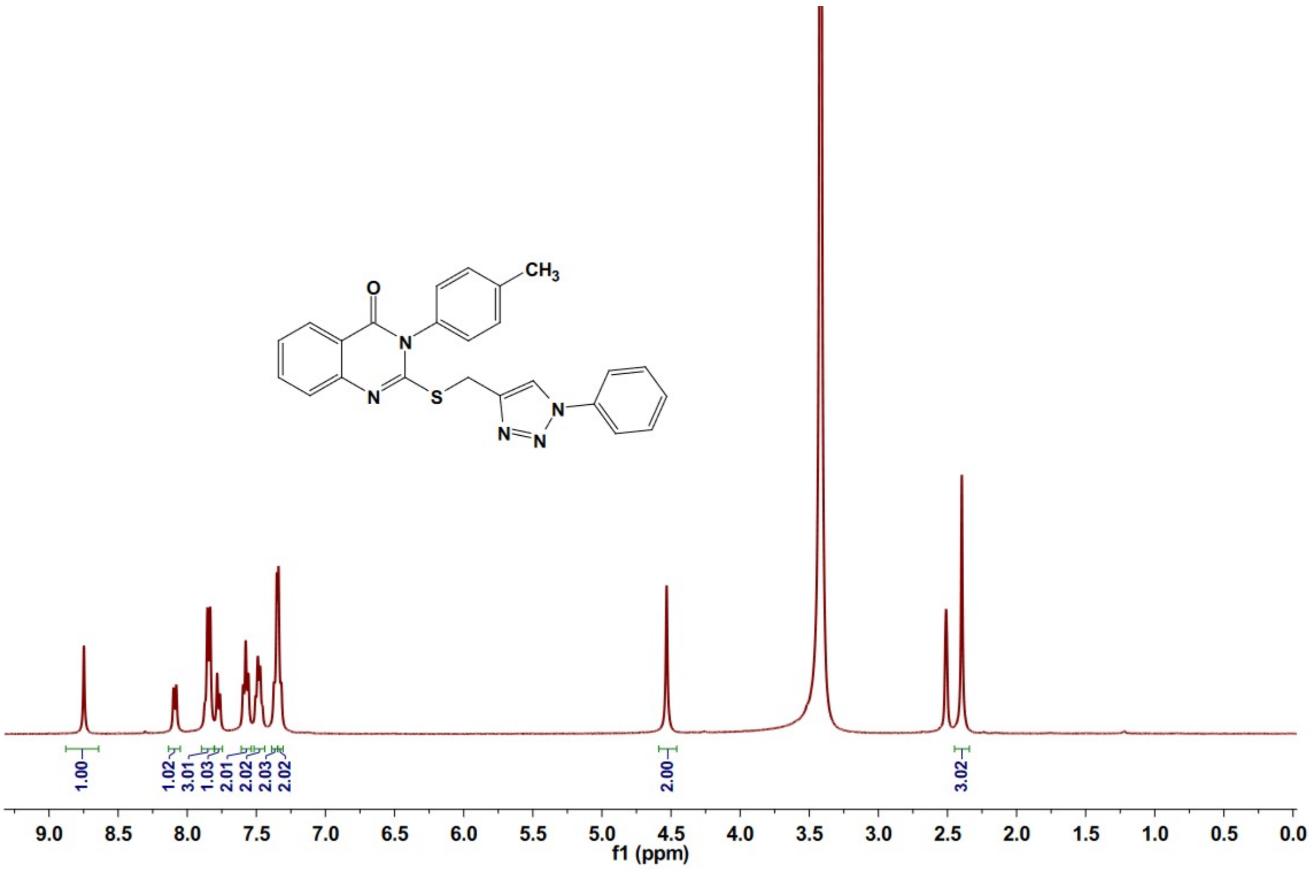

Figure S17: ^{1}H NMR spectrum (400 MHz, DMSO-d_6) of compound **8e**

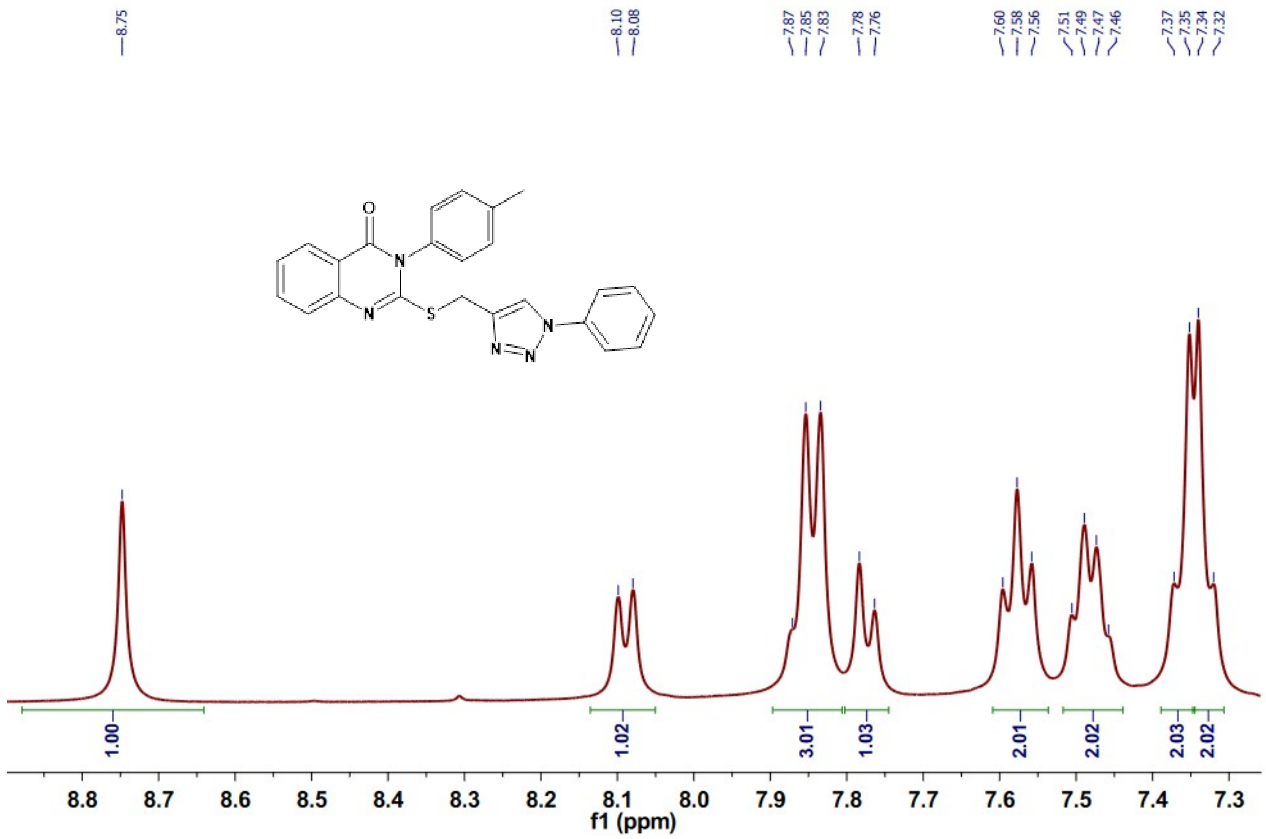

Figure S18: Expanded aromatic region of ^{1}H NMR spectrum (400 MHz, DMSO-d_6) of compound **8e**

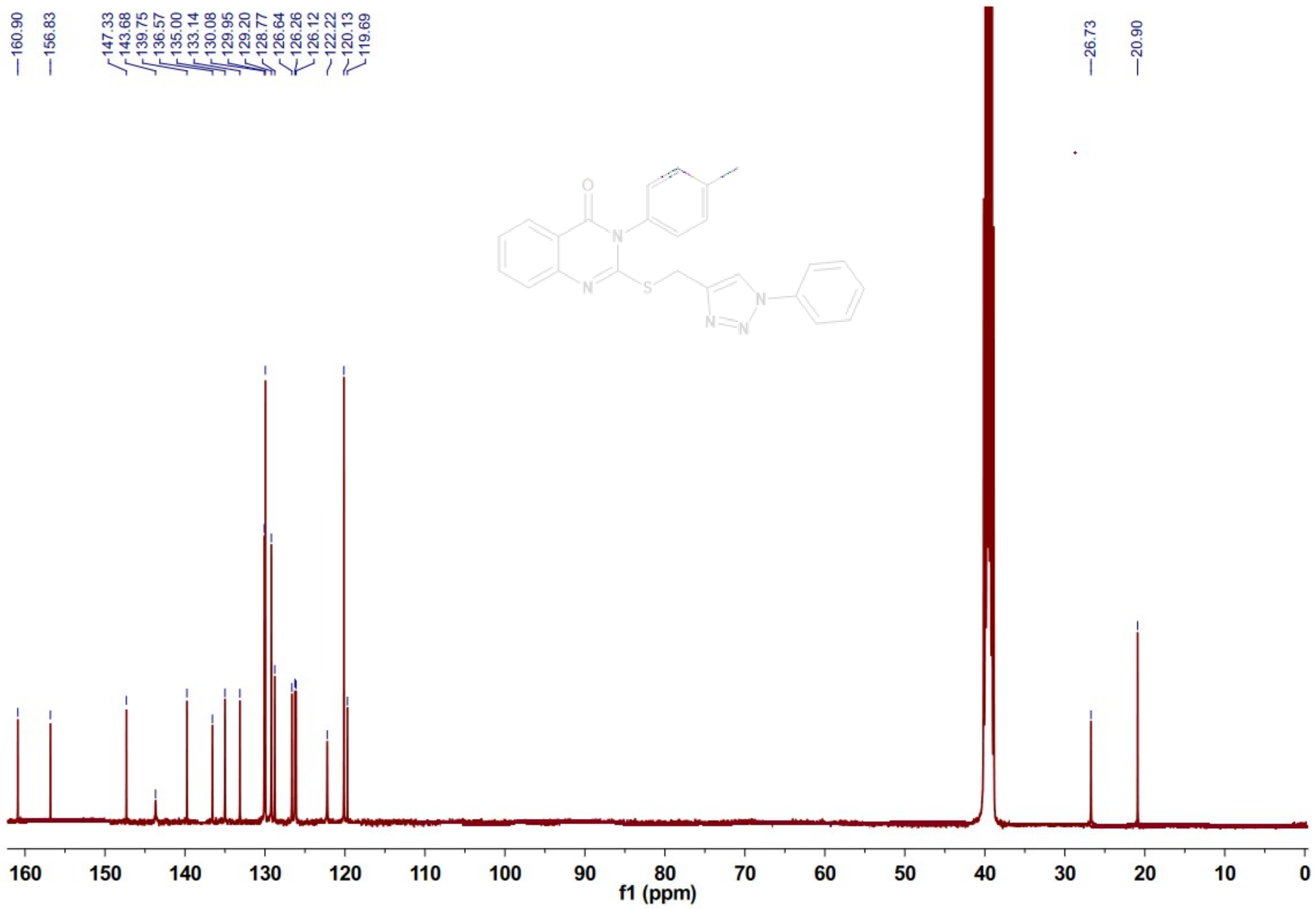

Figure S19: ^{13}C NMR spectrum (100 MHz, DMSO-d_6) of compound **8e**

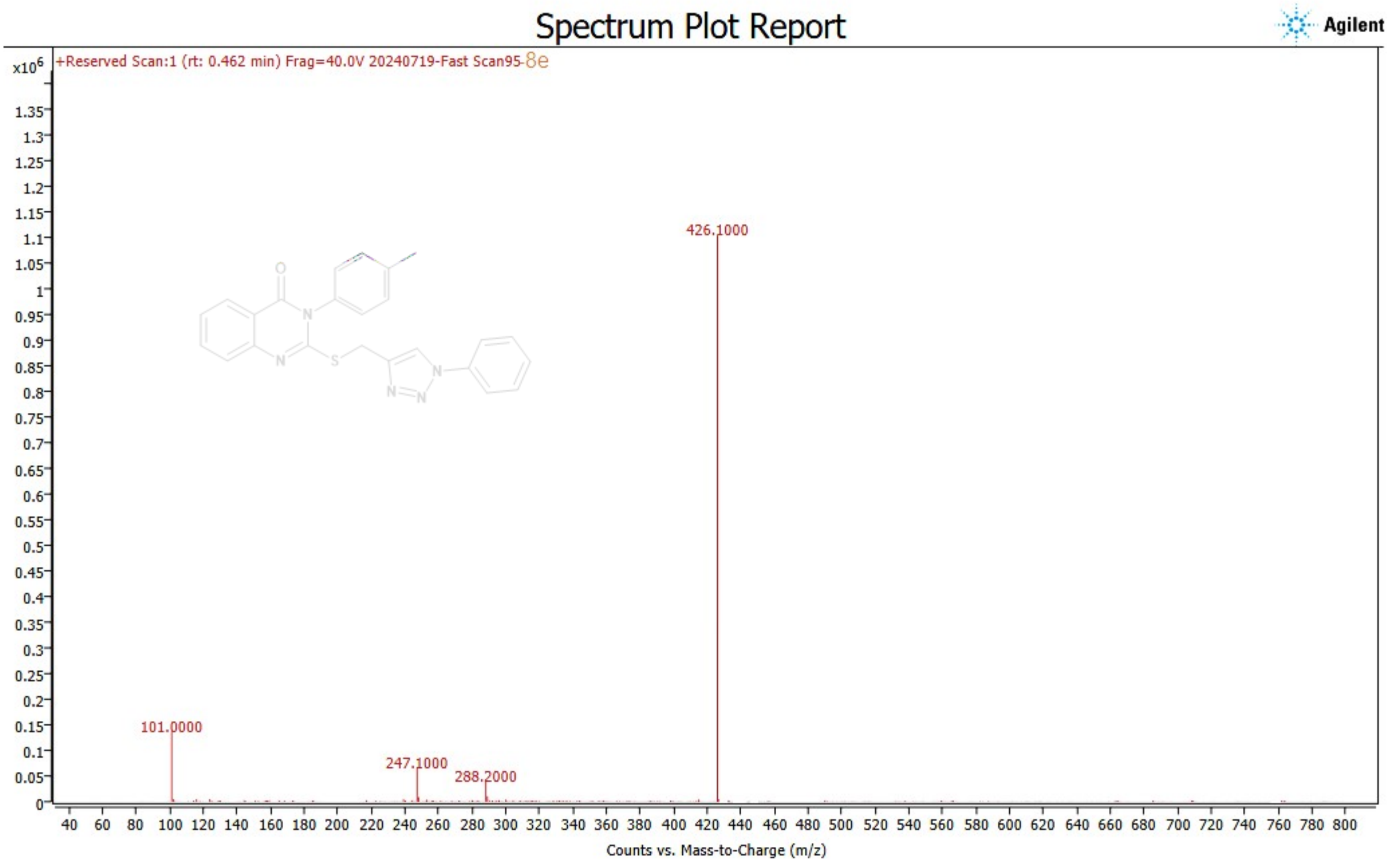

Figure S20: ESI-Mass spectrum of compound **8e**

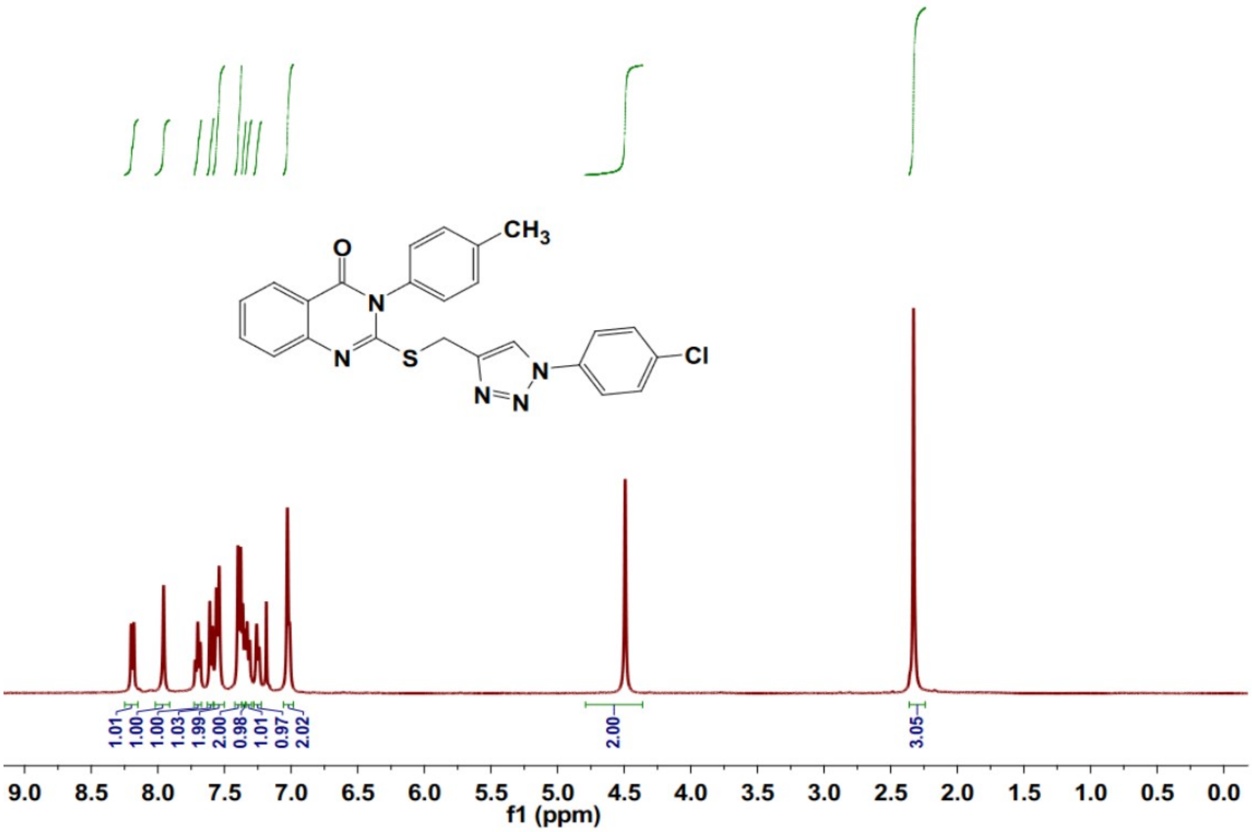

Figure S21: ^{1}H NMR spectrum (400 MHz, $CDCl_3$) of compound **8f**

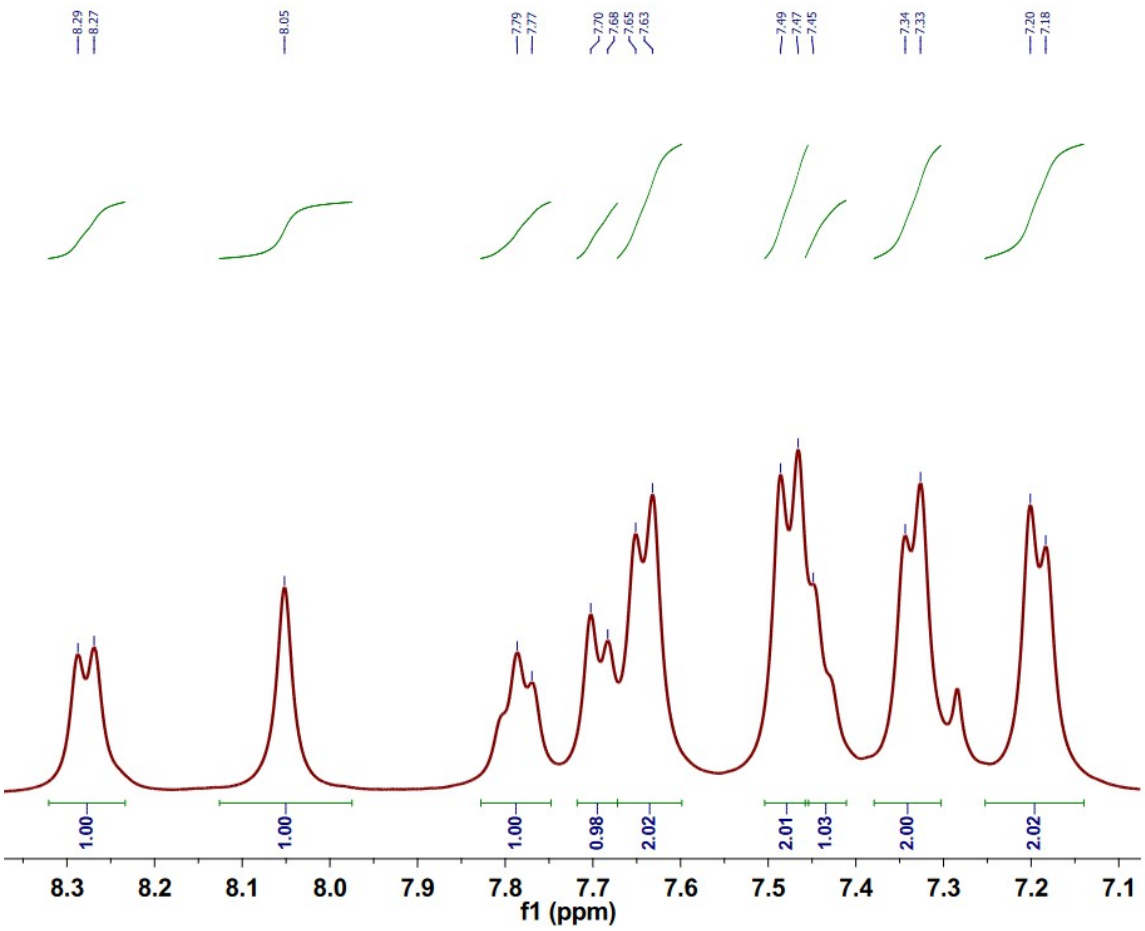

Figure S22: Expanded aromatic region of ^{1}H NMR spectrum (400 MHz, $CDCl_3$) of compound **8f**

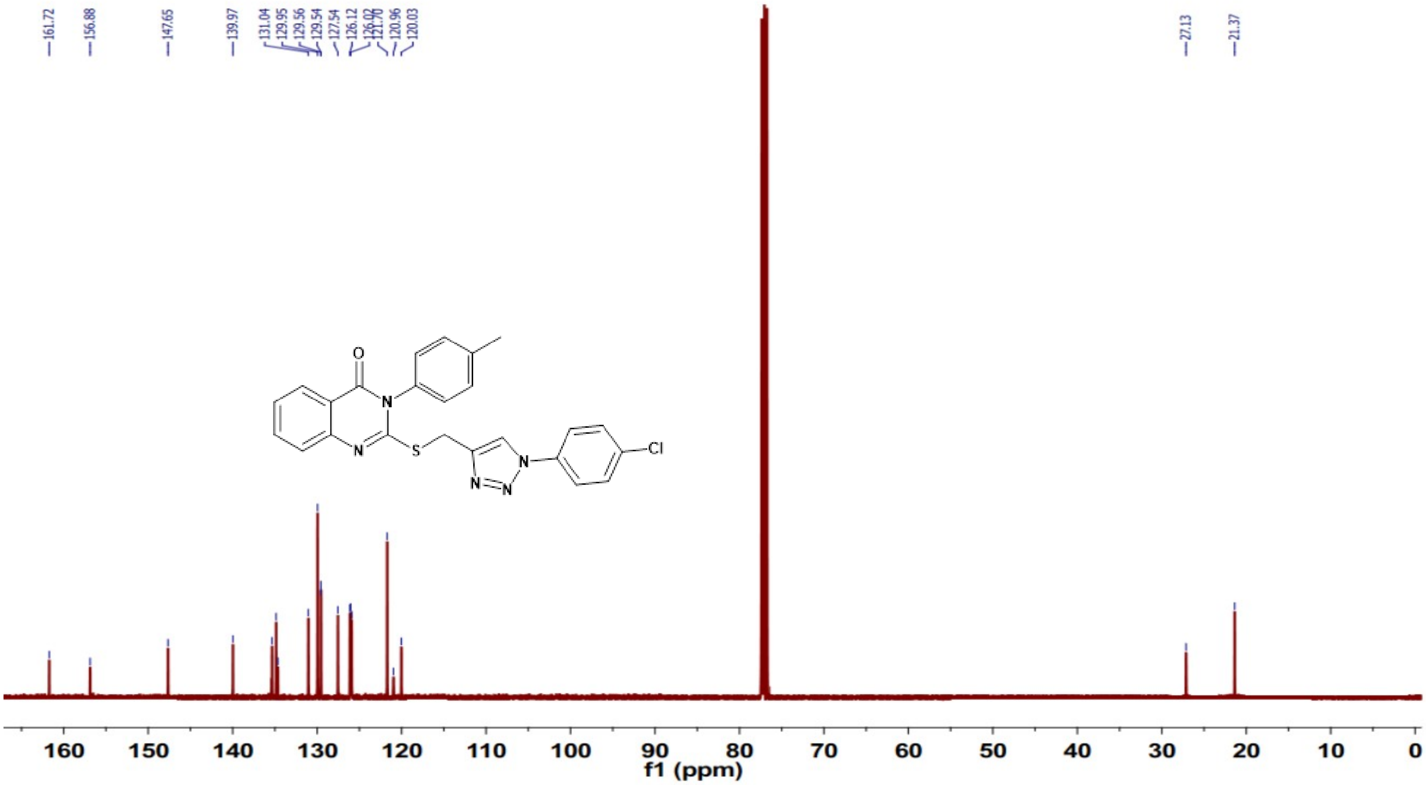

Figure S23: ^{13}C NMR spectrum (100 MHz, $CDCl_3$) of compound **8f**

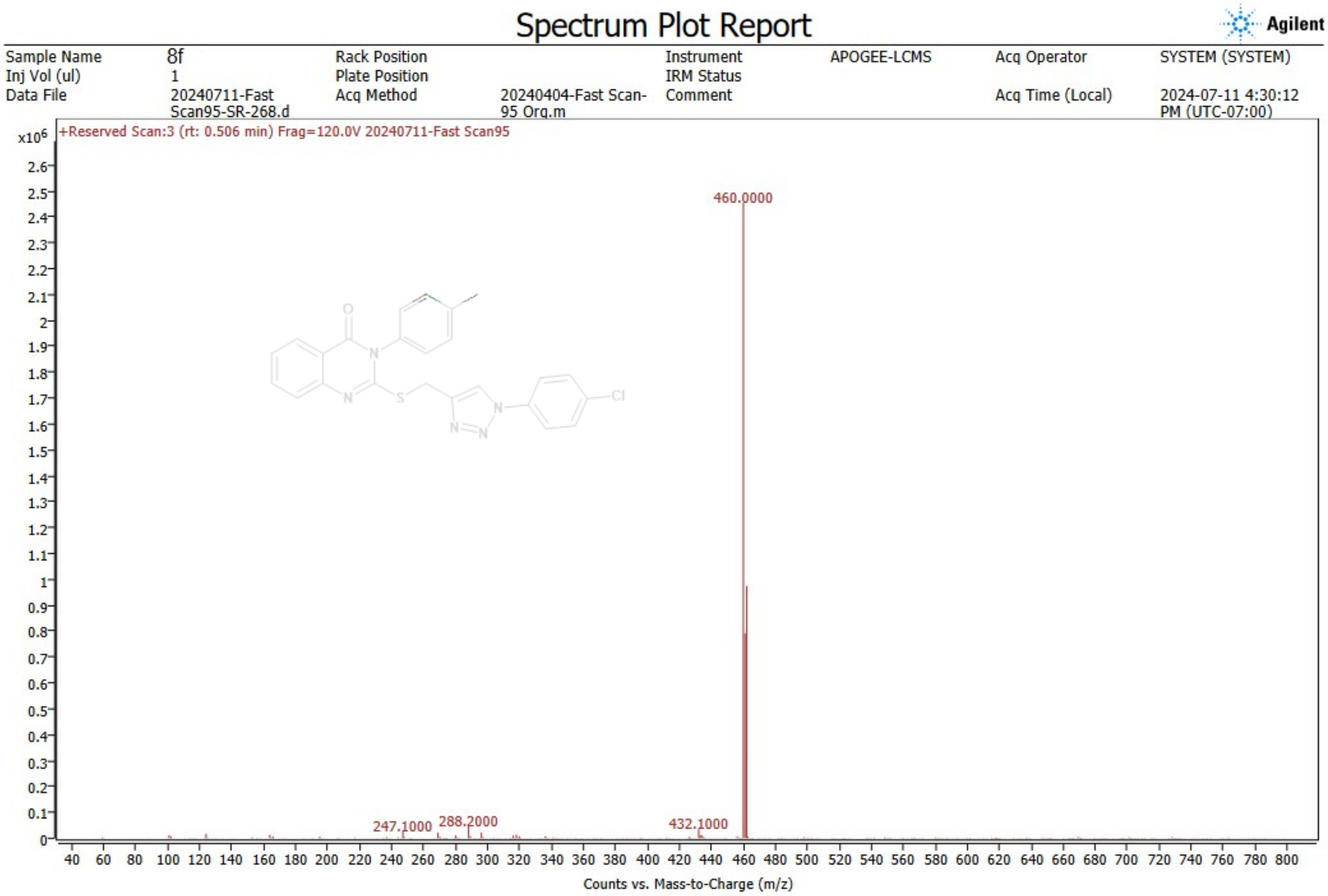

Figure S24: ESI-Mass spectrum of compound **8f**

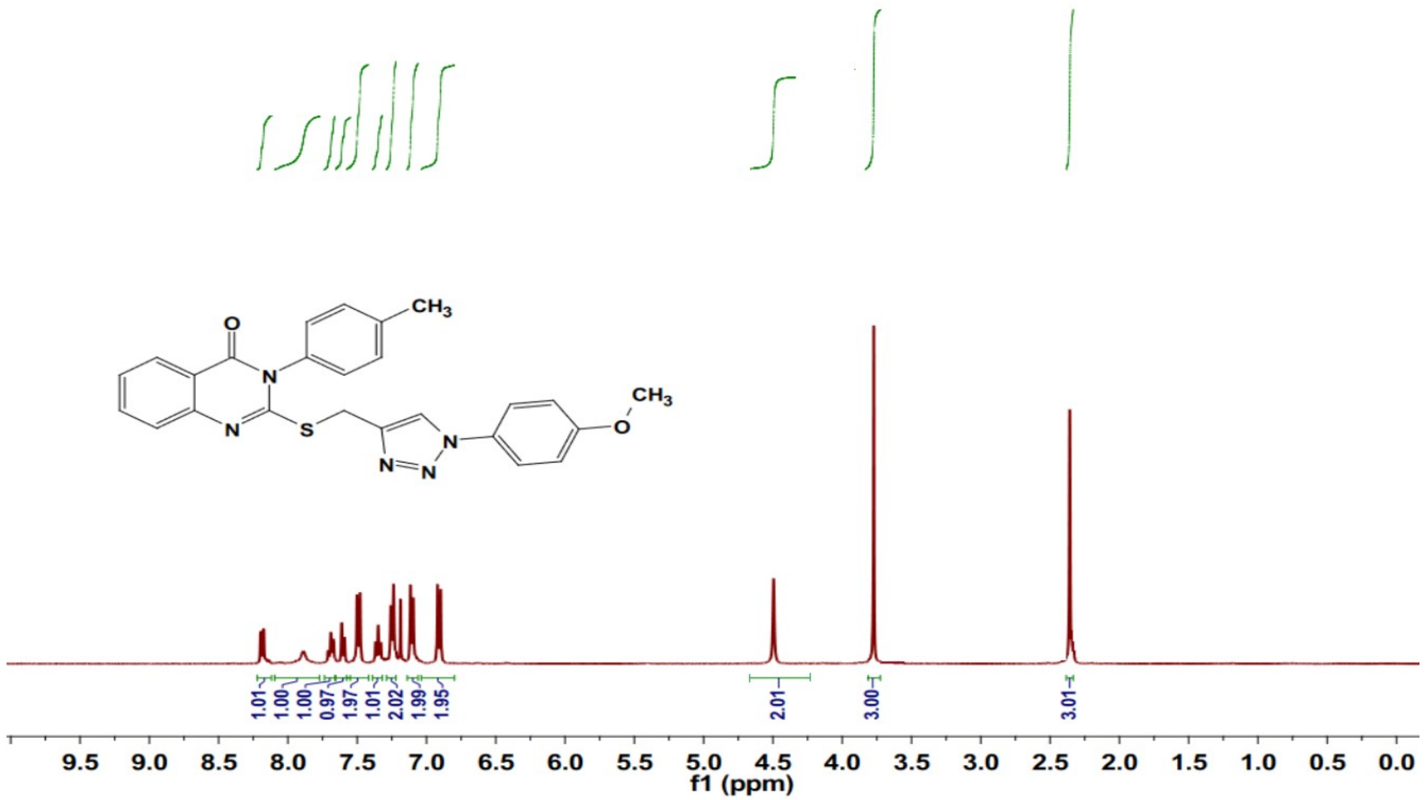

Figure S25: ^{1}H NMR spectrum (400 MHz, $CDCl_3$) of compound **8g**

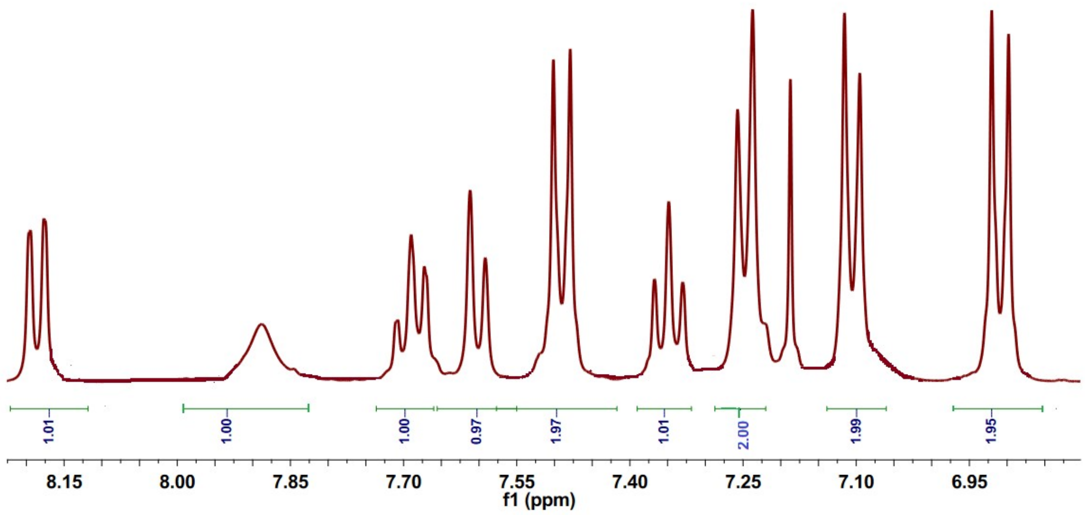

Figure S26: Expanded aromatic region of ^{1}H NMR spectrum (400 MHz, $CDCl_3$) of compound **8g**

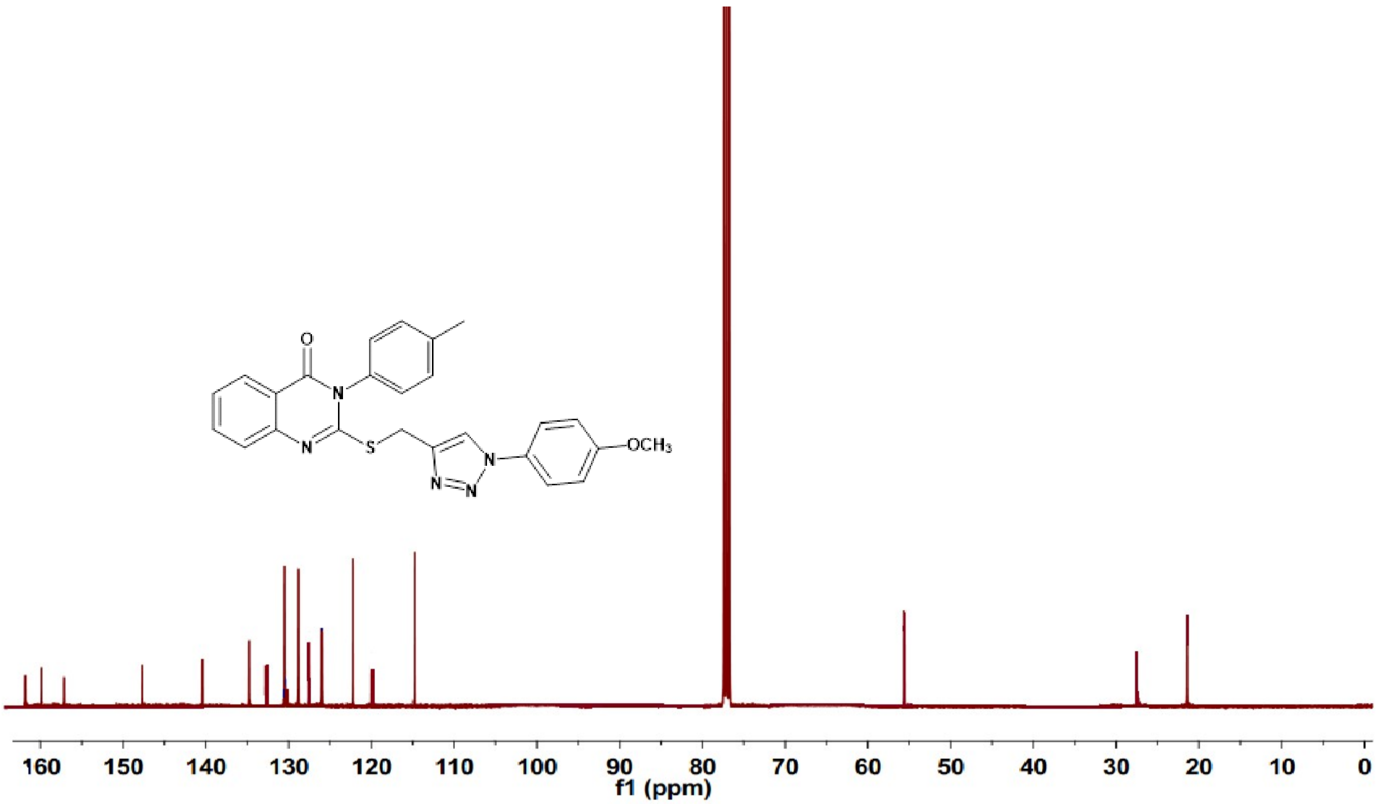

Figure S27: ^{13}C NMR spectrum (100 MHz, $CDCl_3$) of compound **8g**

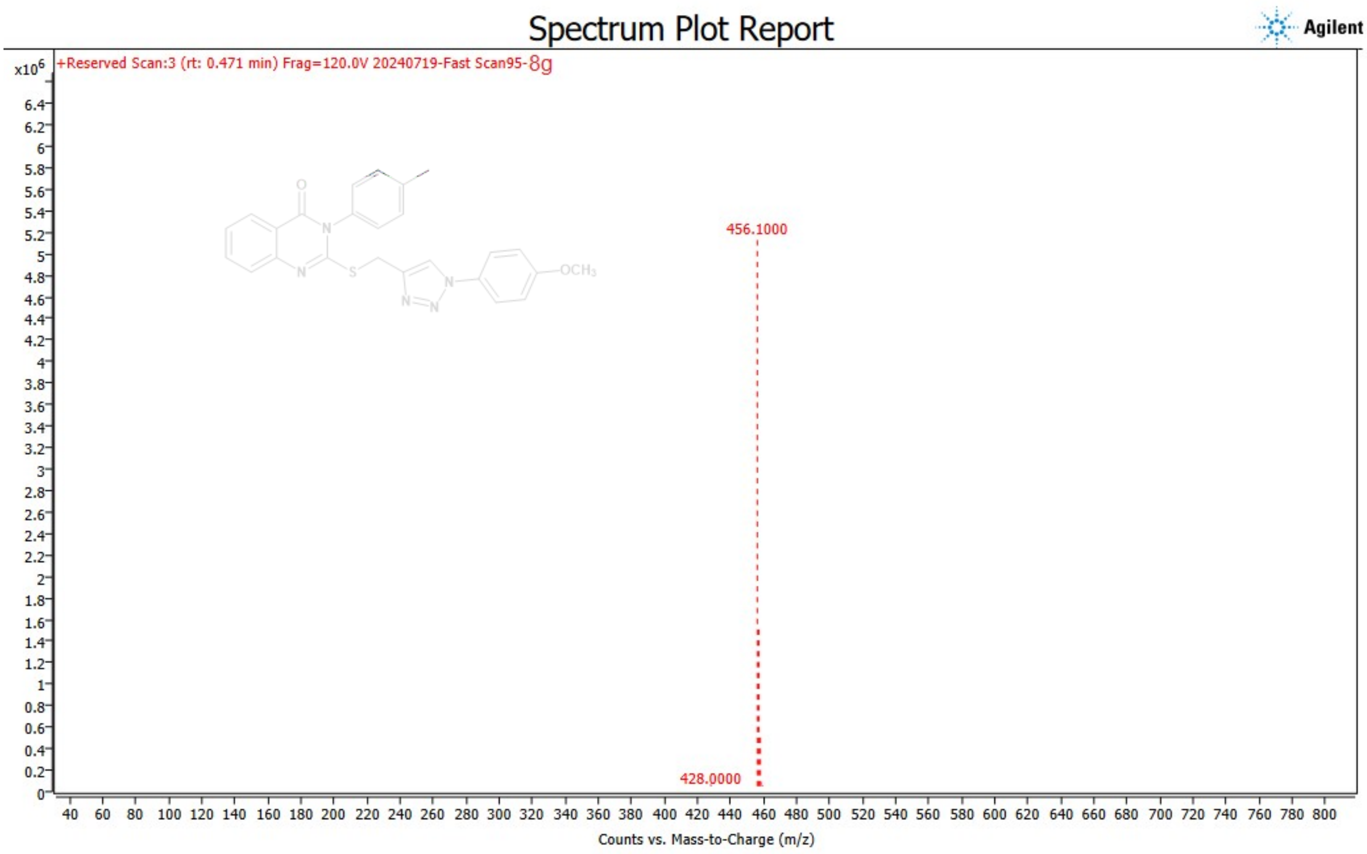

Figure S28: ESI-Mass spectrum of compound **8g**

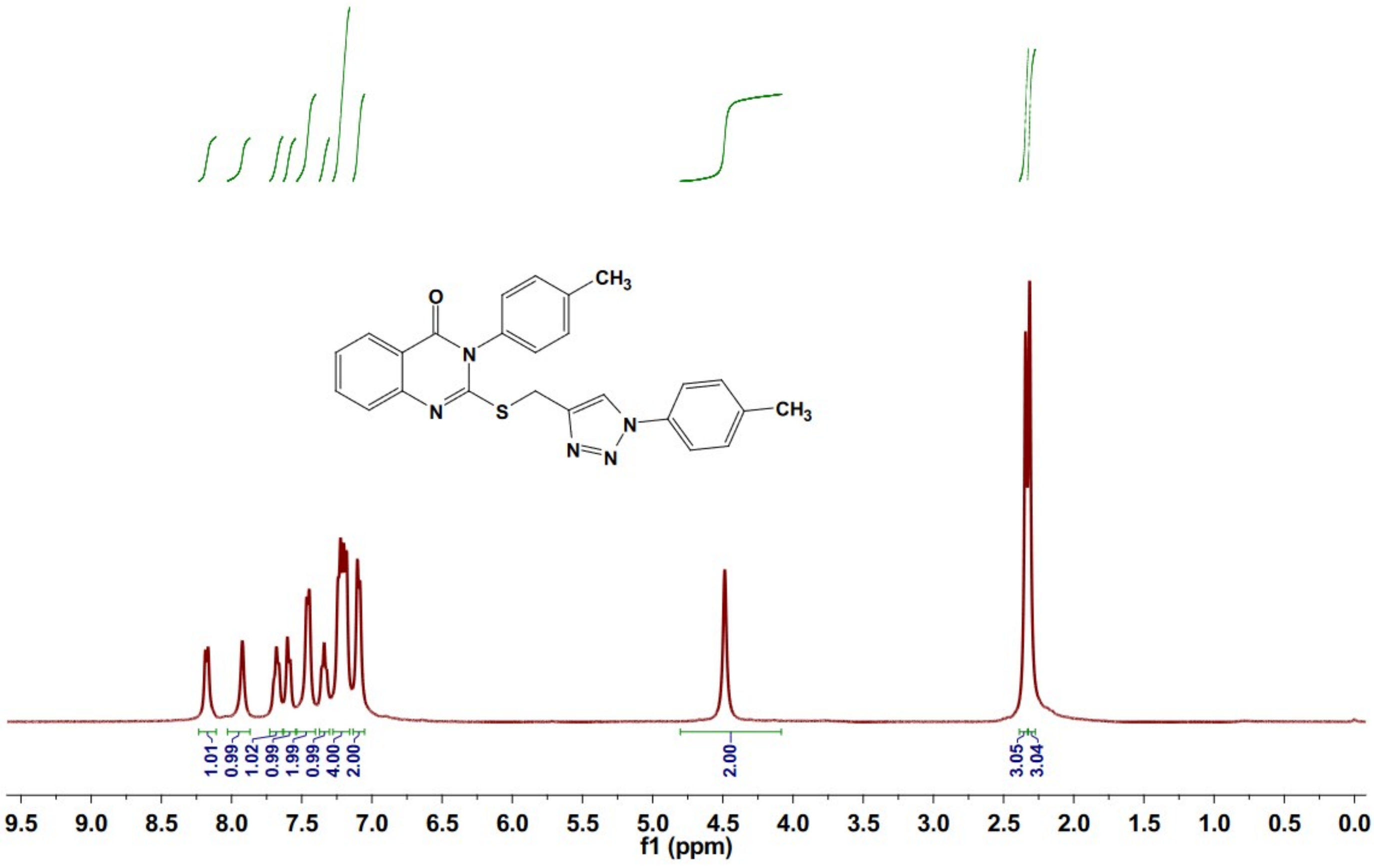

Figure S29: ^{1}H NMR spectrum (400 MHz, $CDCl_3$) of compound **8h**

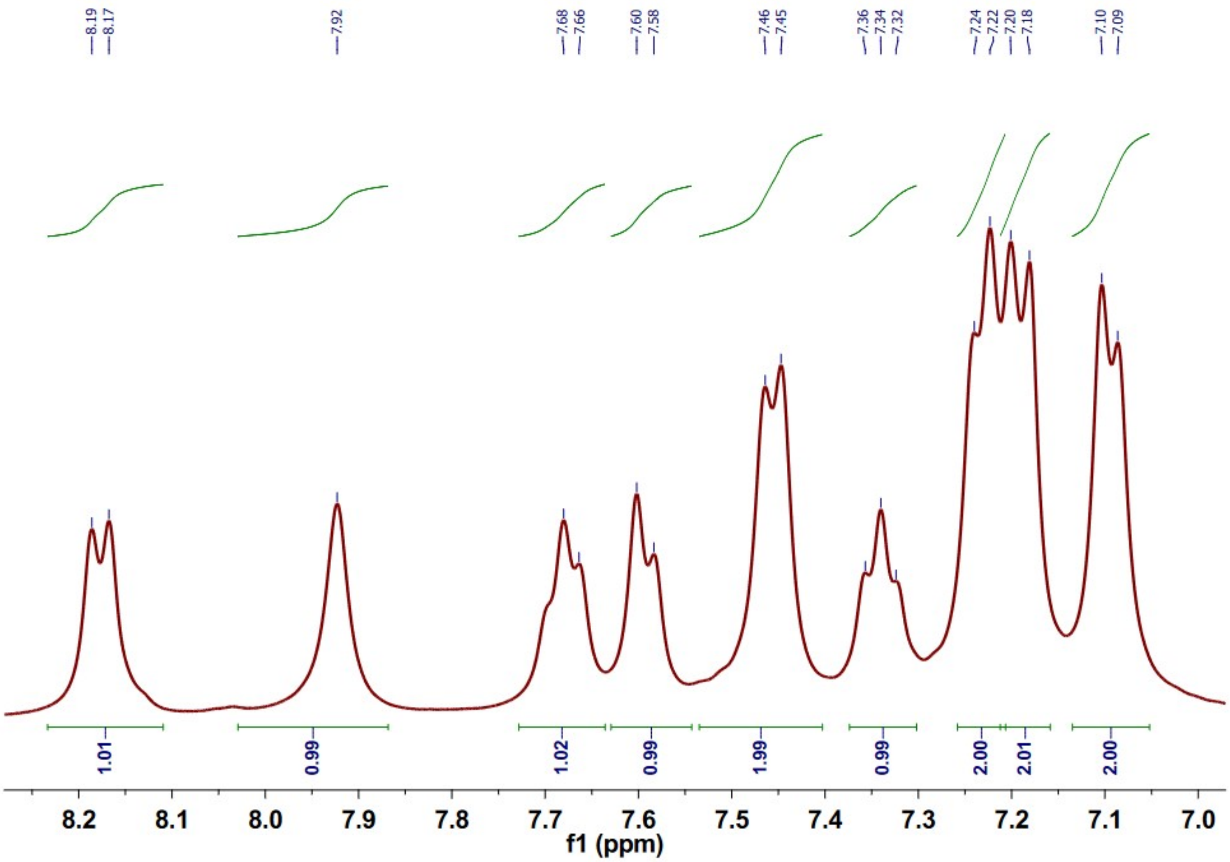

Figure S30: Expanded aromatic region of ^{1}H NMR spectrum (400 MHz, $CDCl_3$) of compound **8h**

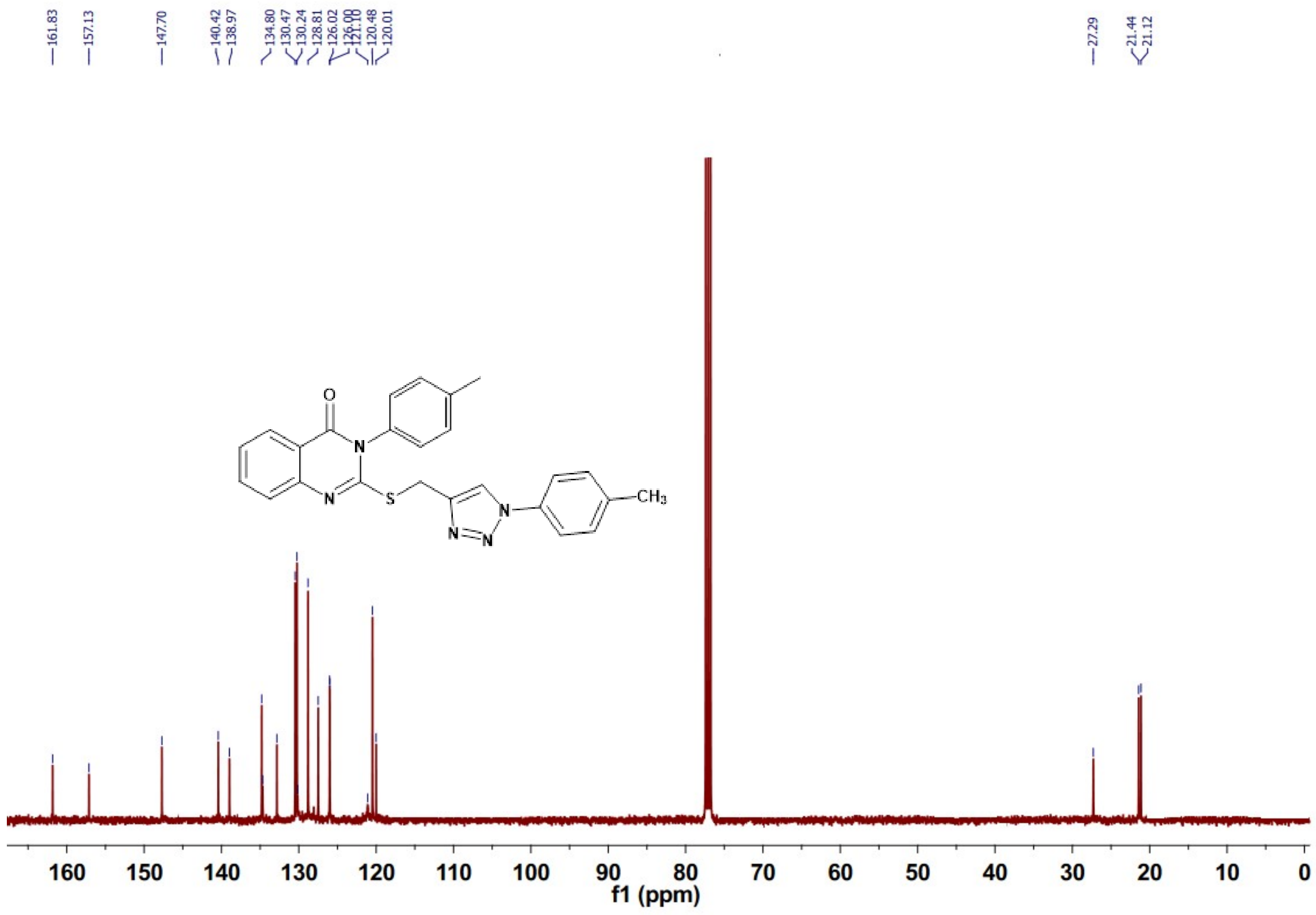

Figure S31: ^{13}C NMR spectrum (100 MHz, $CDCl_3$) of compound **8h**

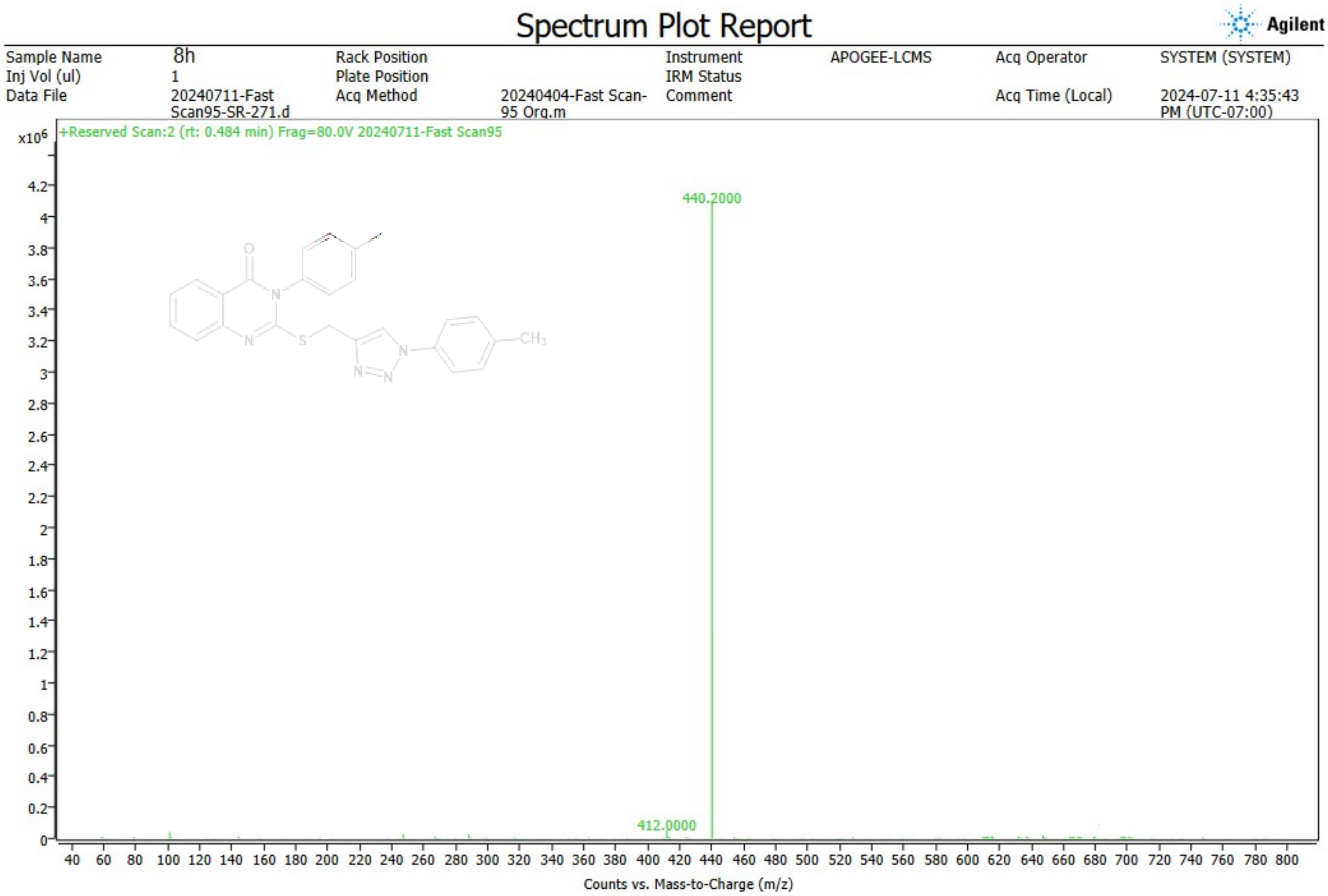

Figure S32: ESI-Mass spectrum of compound **8h**

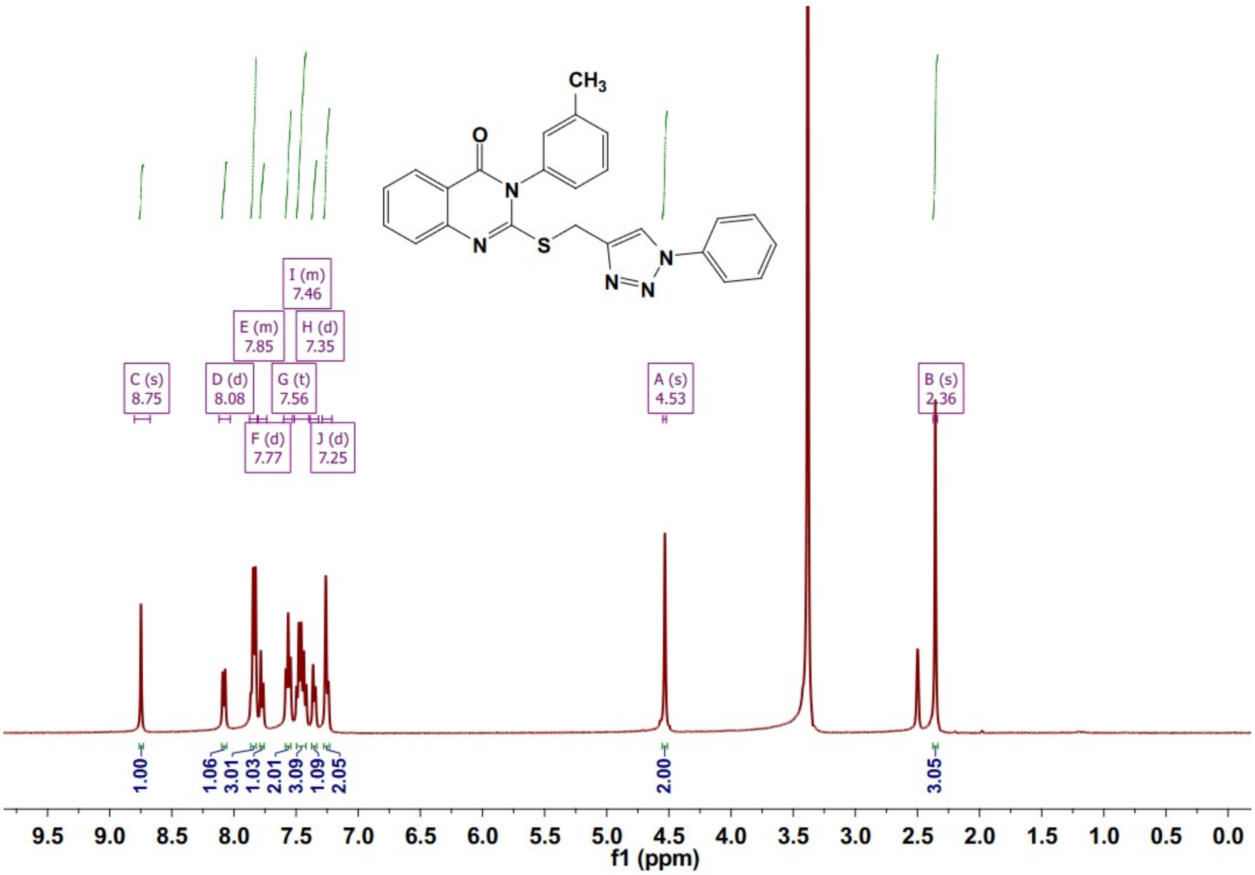

Figure S33: ^{1}H NMR spectrum (400 MHz, DMSO-d_6) of compound **8i**

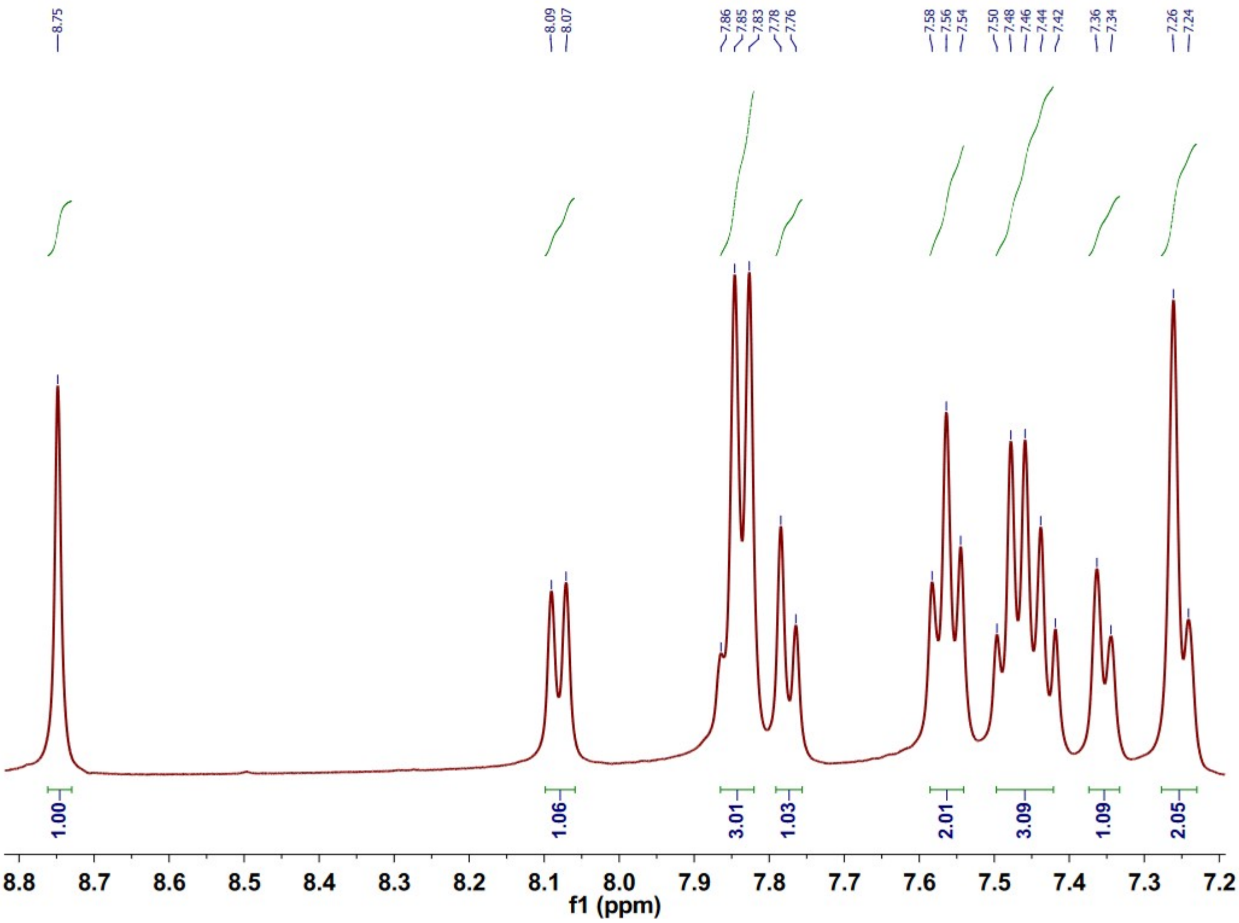

Figure S34: Expanded aromatic region of ^{1}H NMR spectrum (400 MHz, DMSO-d_6) of compound **8i**

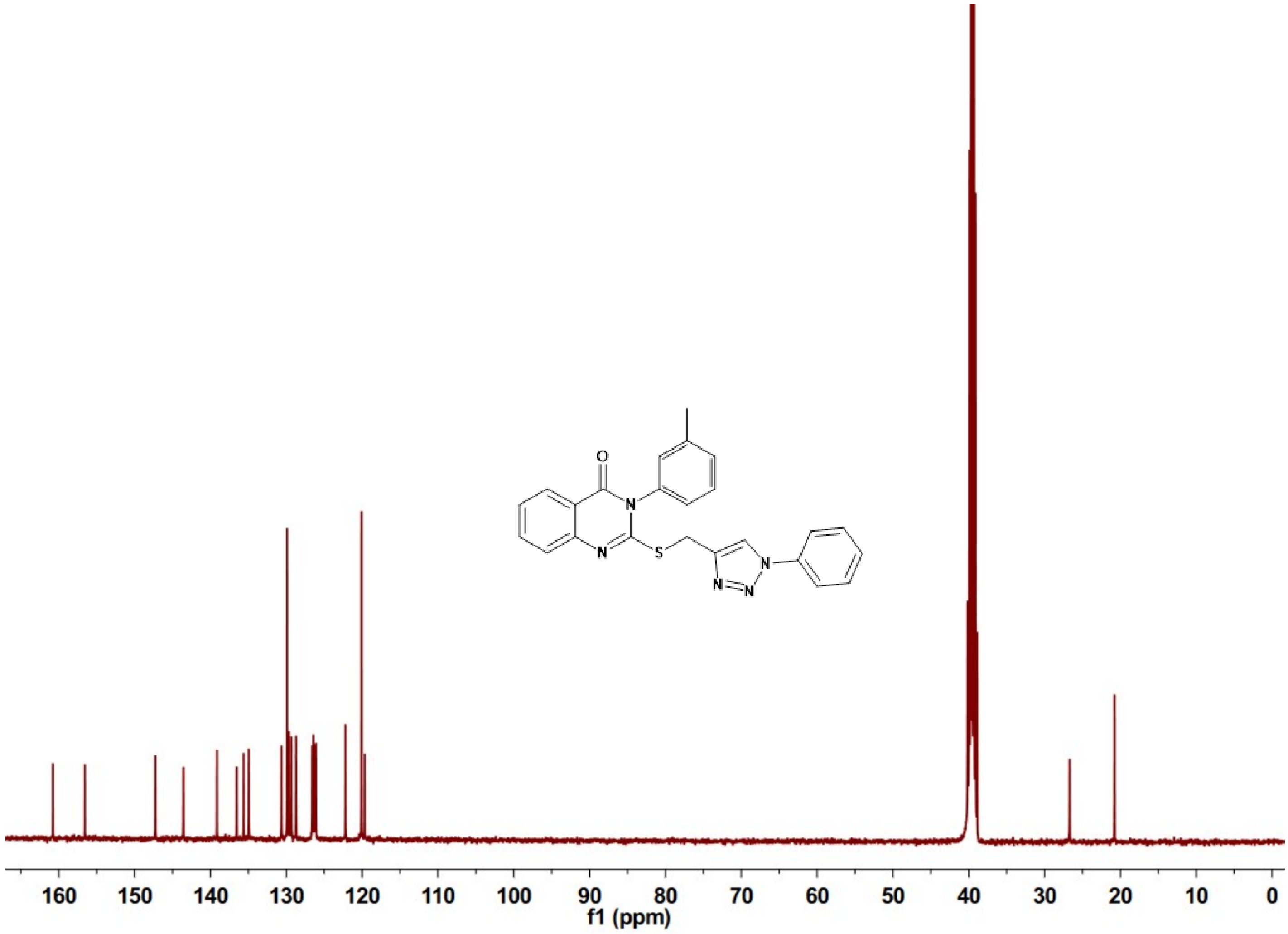

Figure S35: ^{13}C NMR spectrum (100 MHz, DMSO-d_6) of compound **8i**

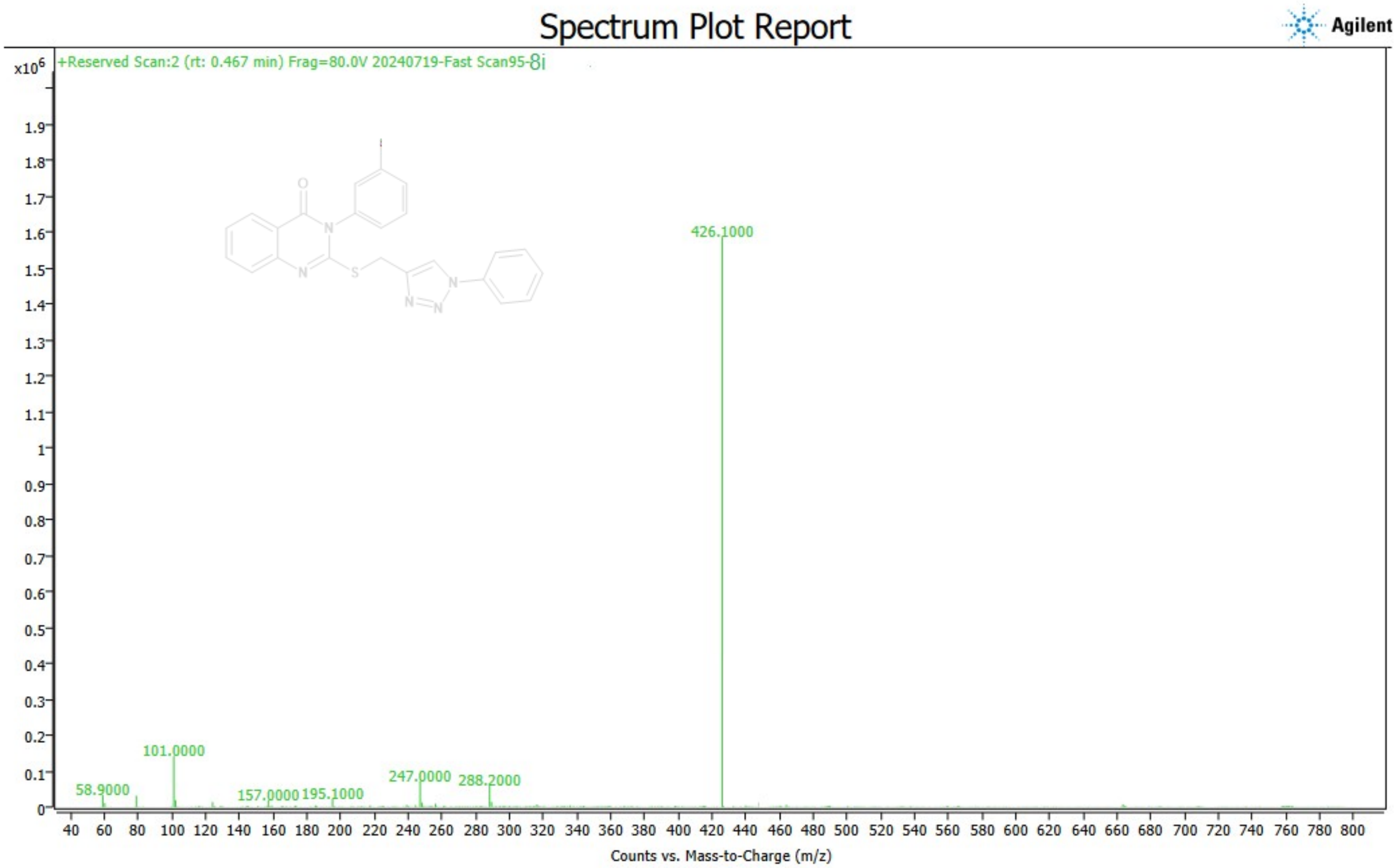

Figure S36: ESI-Mass spectrum of compound **8i**

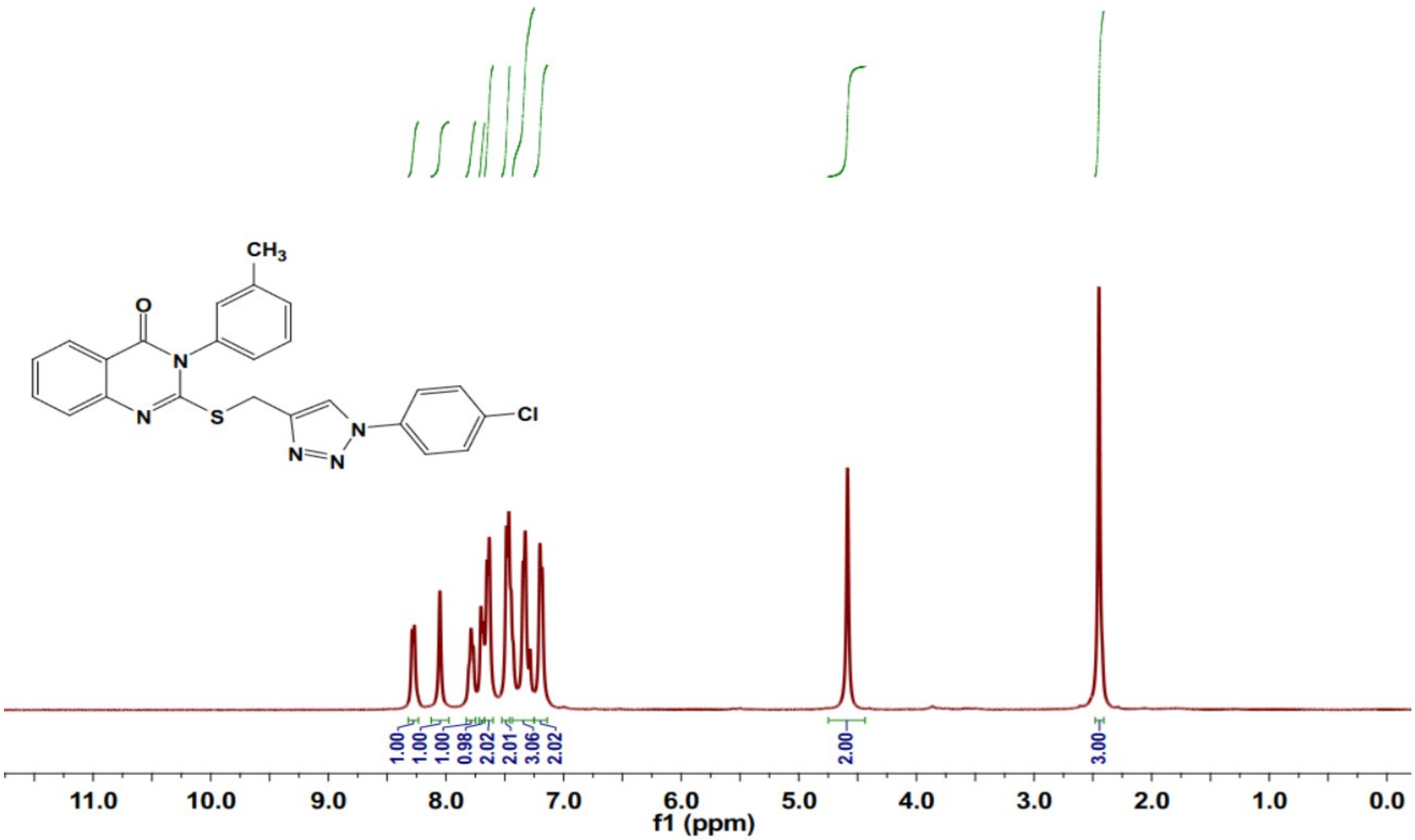

Figure S37: ^{1}H NMR spectrum (400 MHz, $CDCl_3$) of compound **8j**

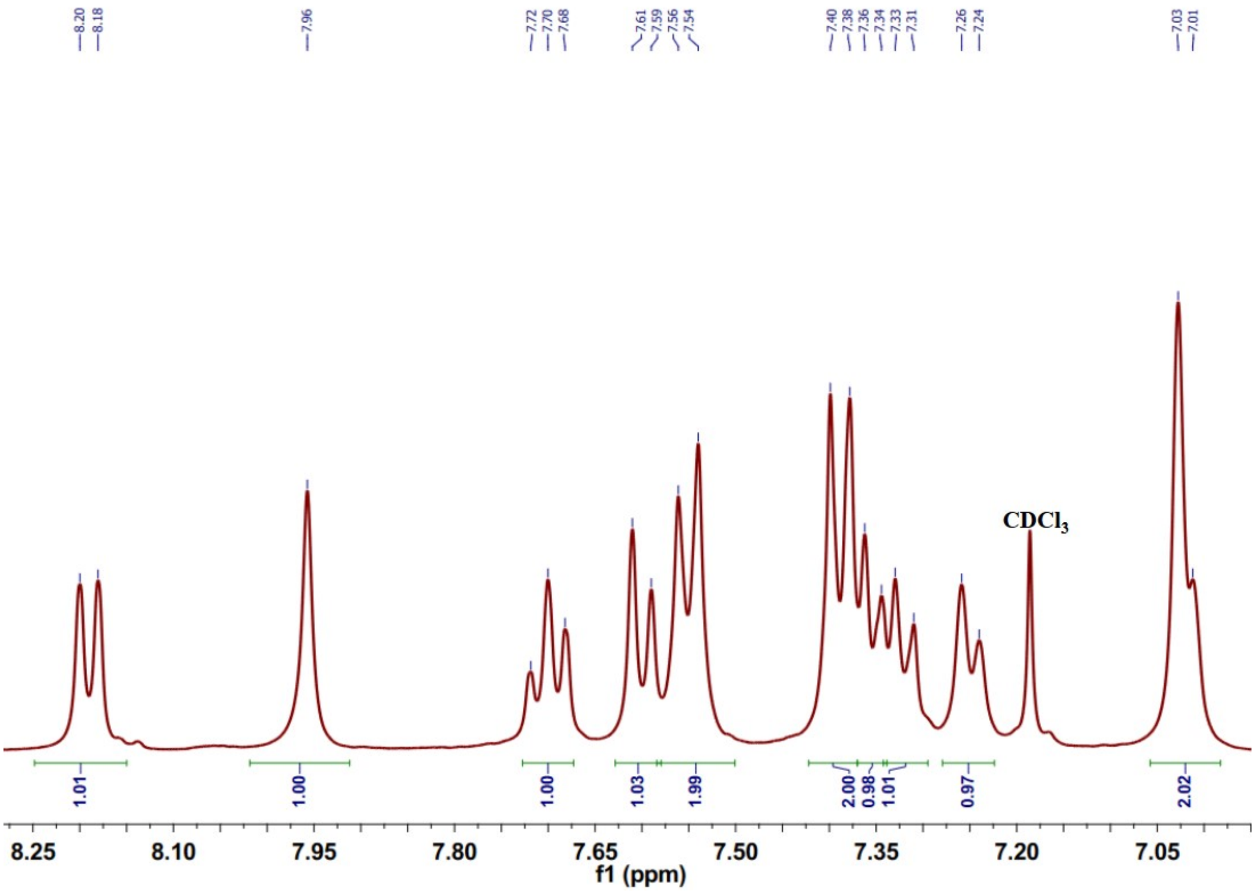

Figure S38: Expanded aromatic region of ^{1}H NMR spectrum (400 MHz, $CDCl_3$) of compound

8j

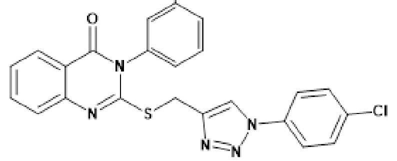

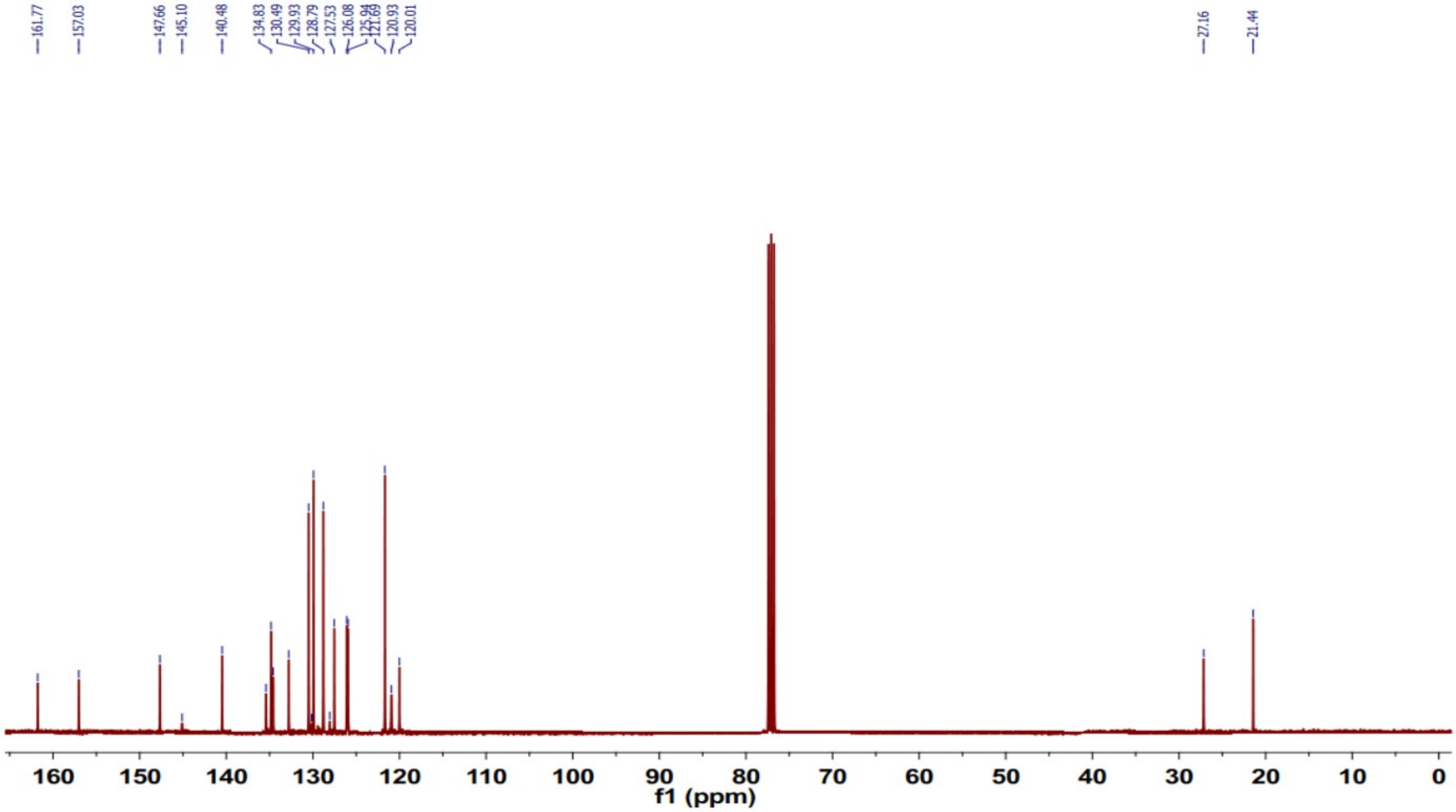

Figure S39: ^{13}C NMR spectrum (100 MHz, $CDCl_3$) of compound **8j**

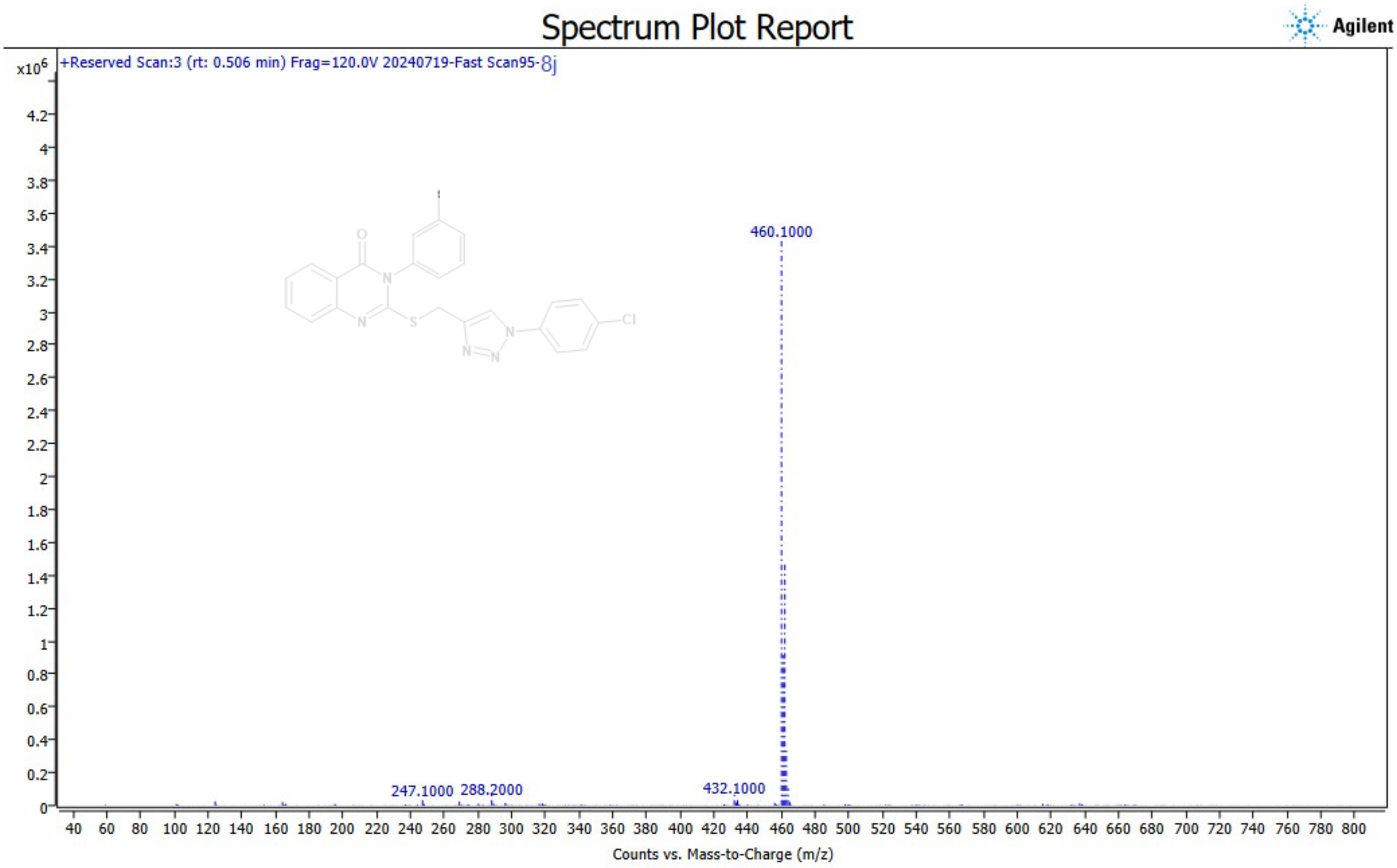

Figure S40: ESI-Mass spectrum of compound **8j**

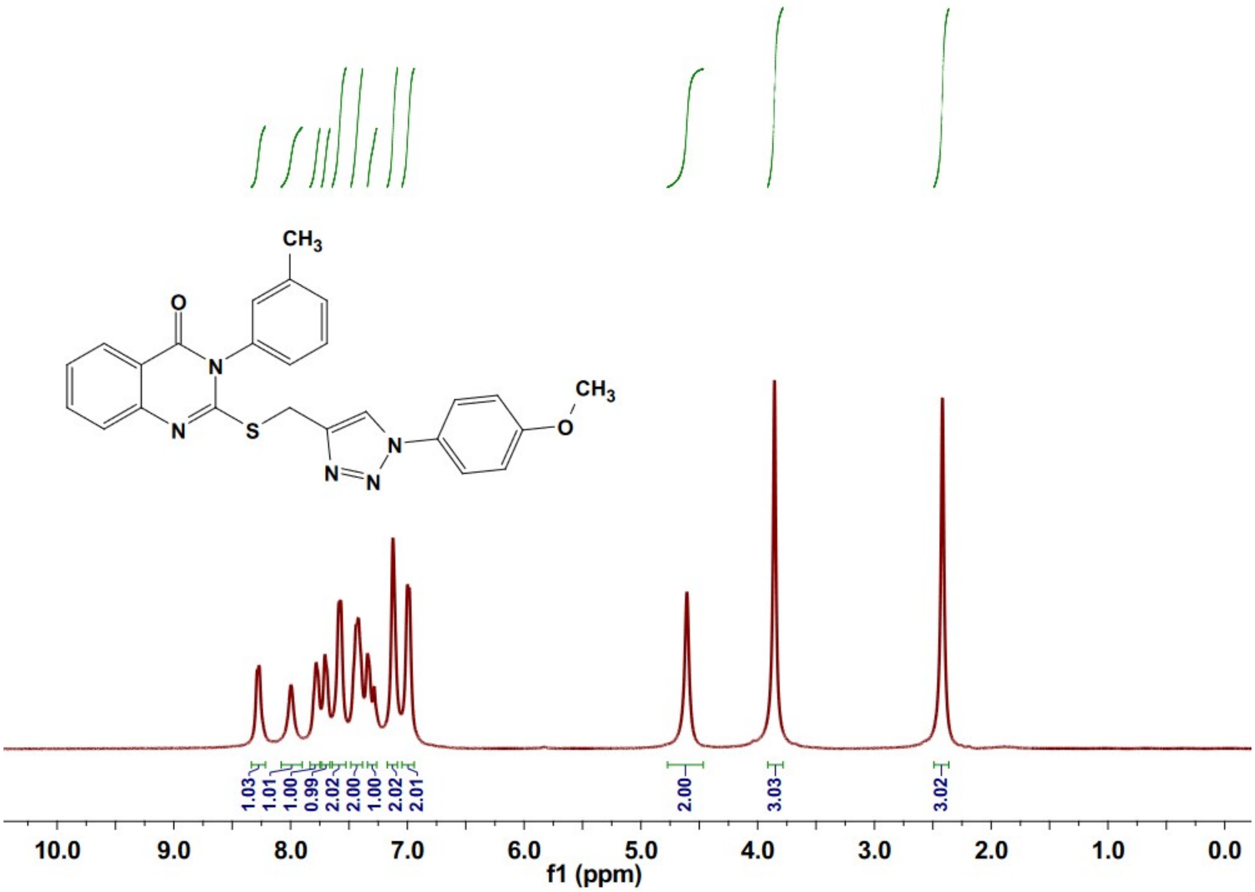

Figure S41: ^{1}H NMR spectrum (400 MHz, $CDCl_3$) of compound **8k**

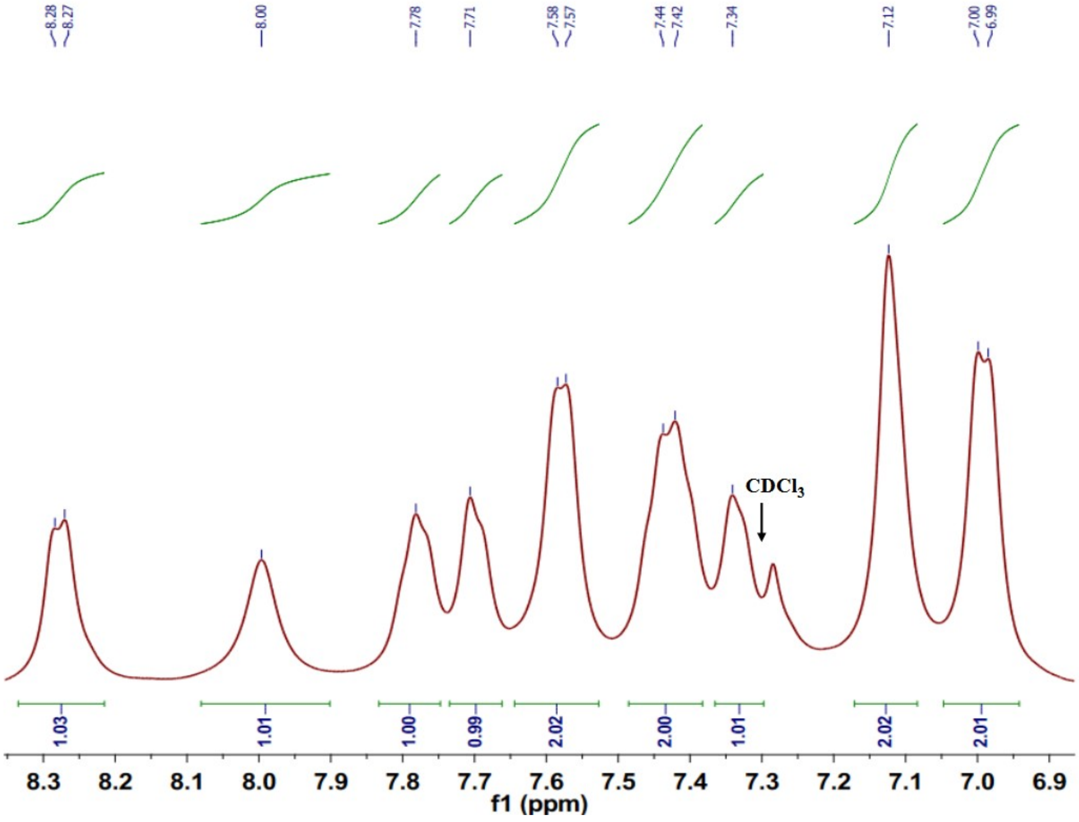

Figure S42: Expanded aromatic region of ^{1}H NMR spectrum (400 MHz, $CDCl_3$) of compound **8k**

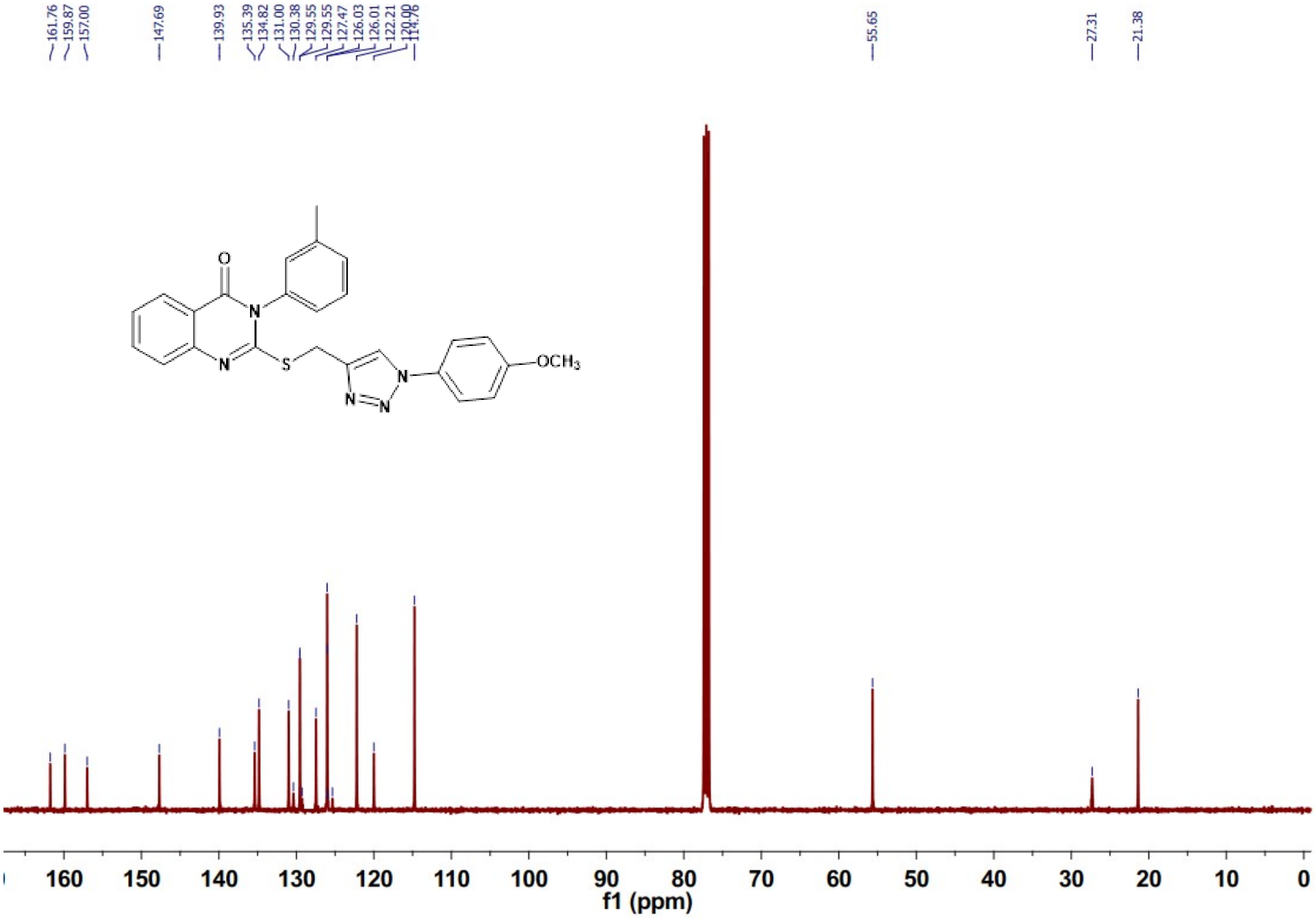

Figure S43: ^{13}C NMR spectrum (100 MHz, $CDCl_3$) of compound **8k**

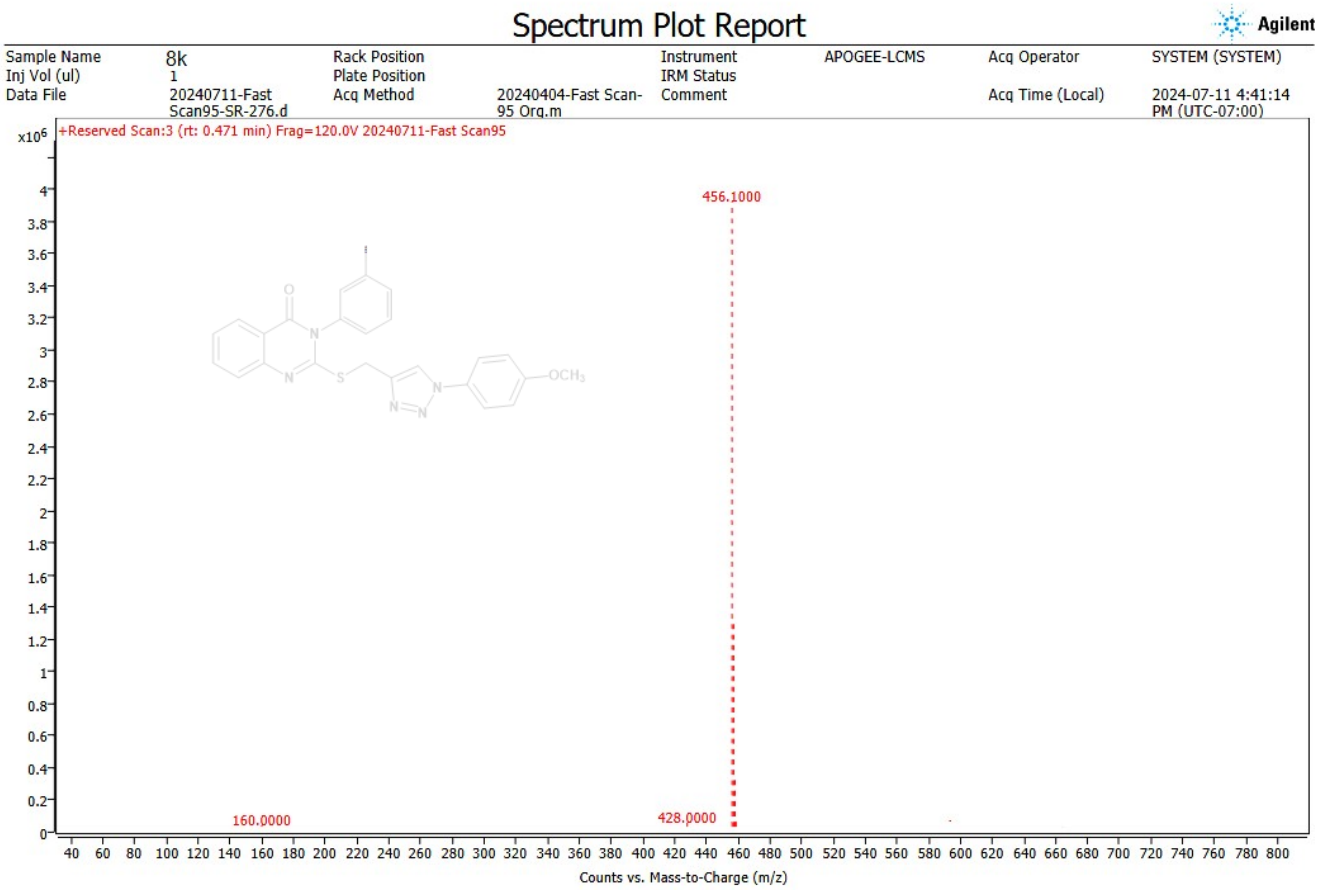

Figure S44: ESI-Mass spectrum of compound **8k**

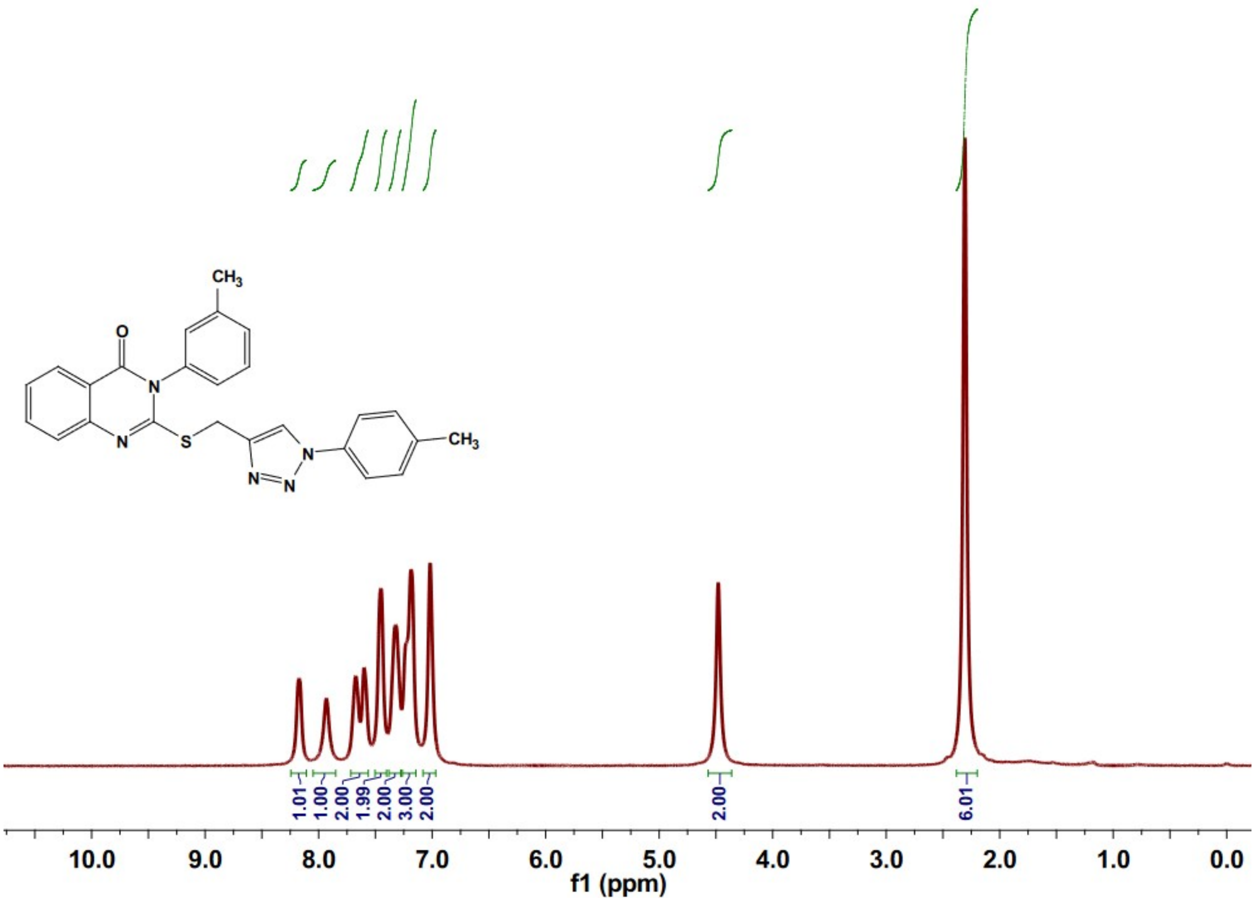

Figure S45: ^{1}H NMR spectrum (400 MHz, $CDCl_3$) of compound **8l**

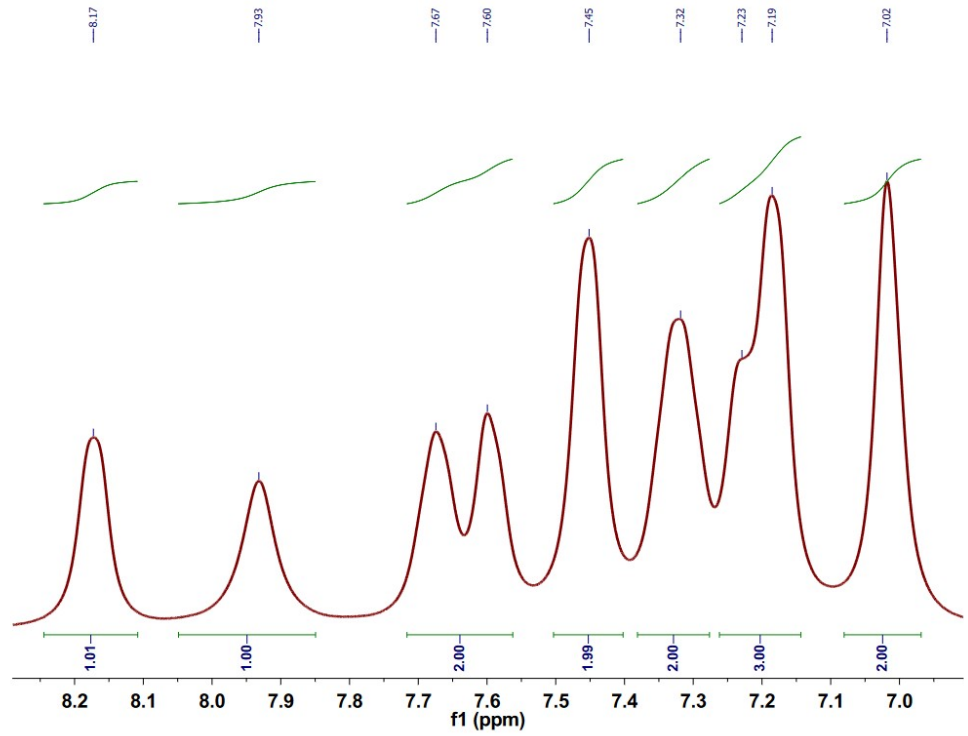

Figure S46: Expanded aromatic region of ^{1}H NMR spectrum (400 MHz, $CDCl_3$) of compound **8l**

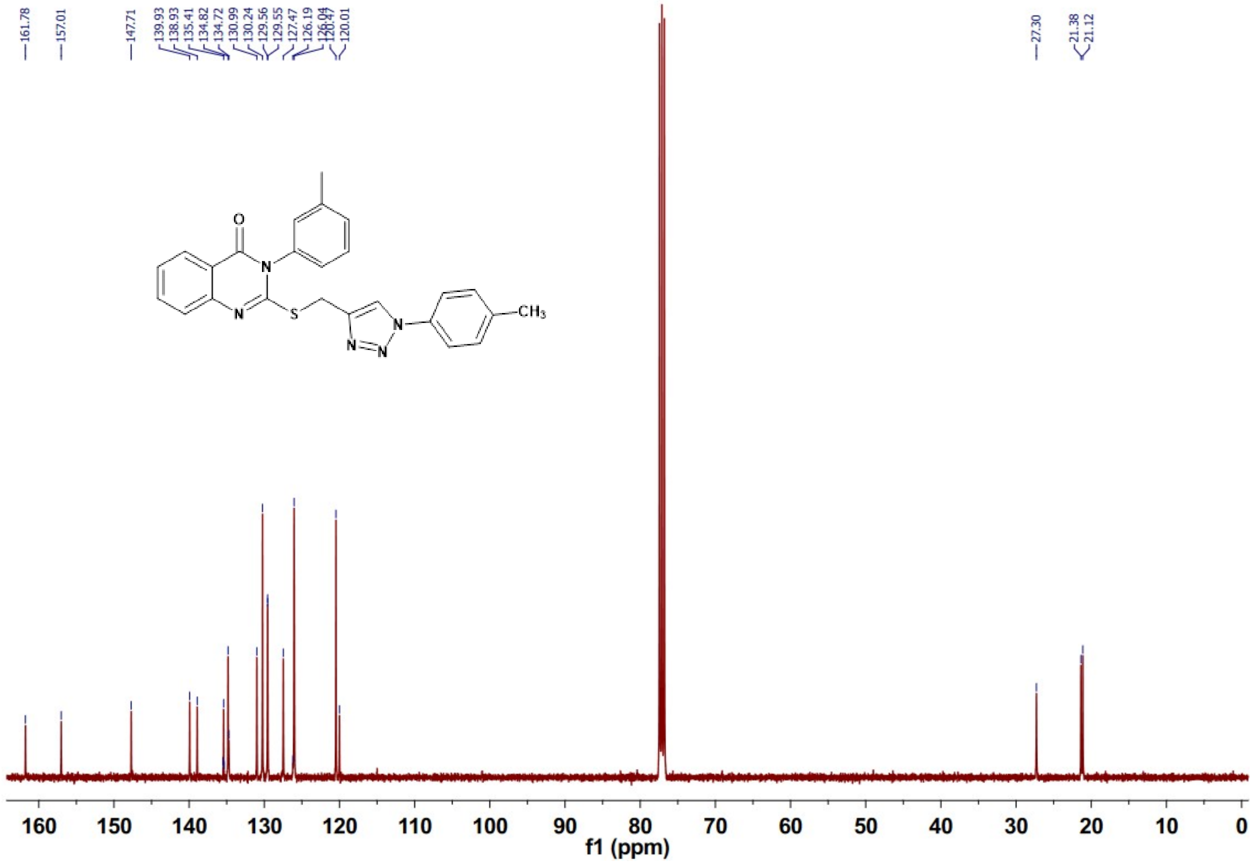

Figure S47: ^{13}C NMR spectrum (100 MHz, $CDCl_3$) of compound **8l**

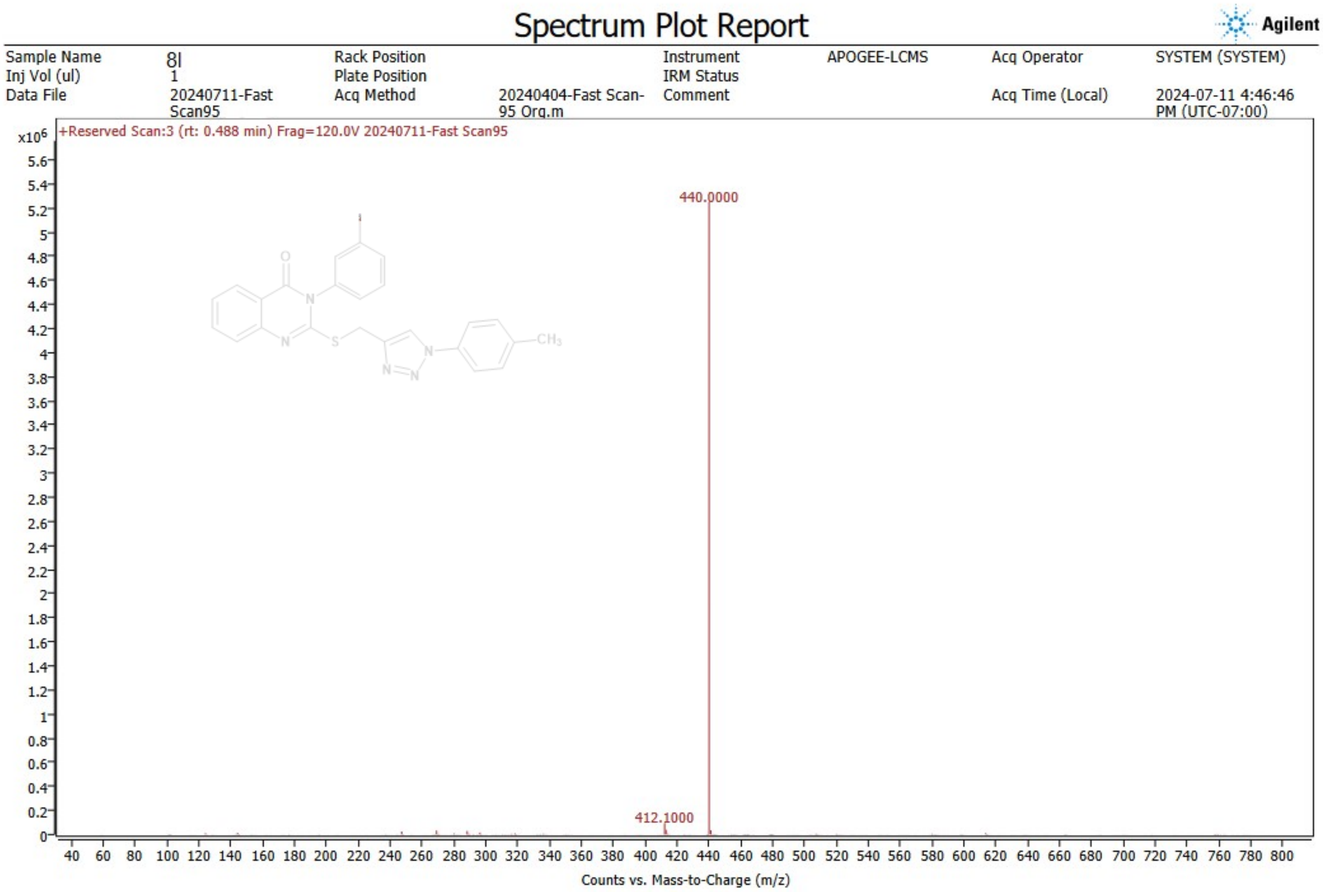

Figure S48: ESI-Mass spectrum of compound **8l**

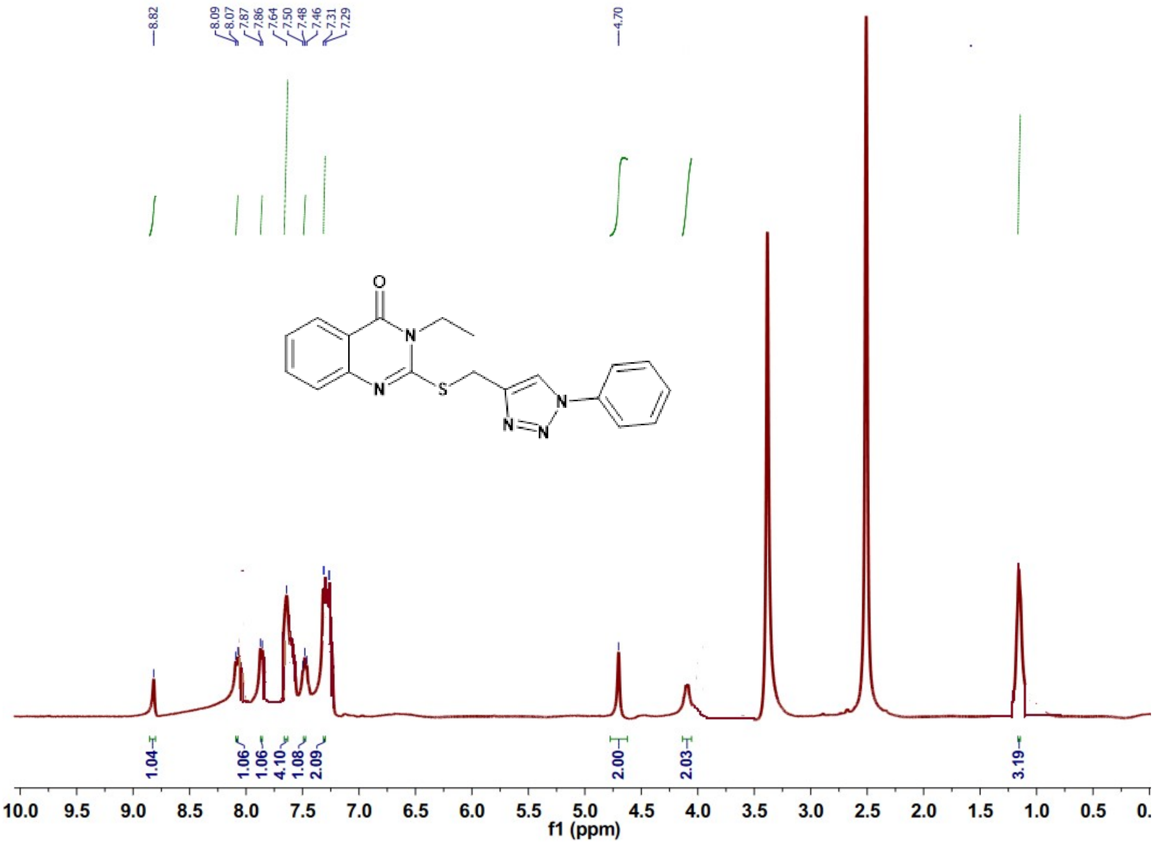

Figure S49: ^{1}H NMR spectrum (400 MHz, DMSO-d$_6$) of compound **8m**

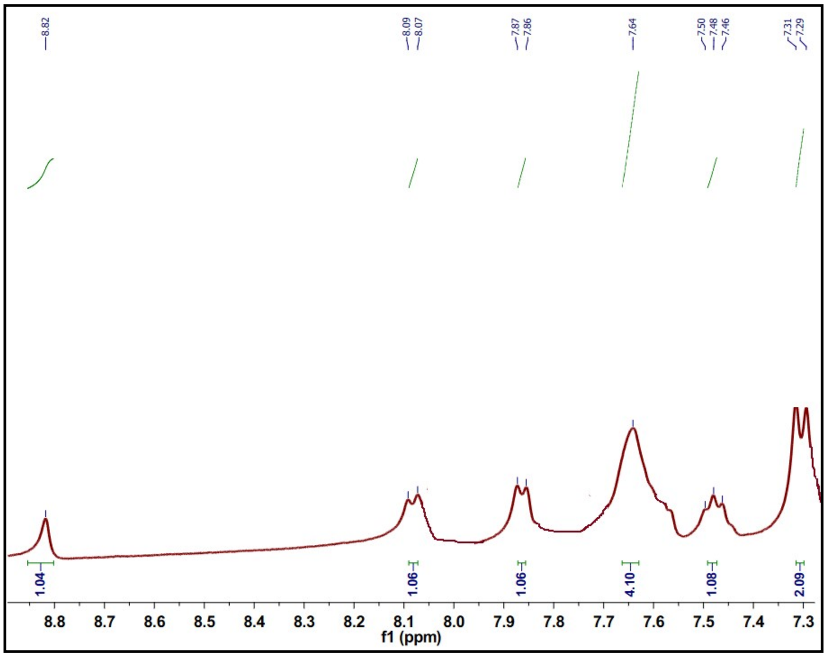

Figure S50: Expanded aromatic region of ^{1}H NMR spectrum (400 MHz, DMSO-d$_6$) of compound **8m**

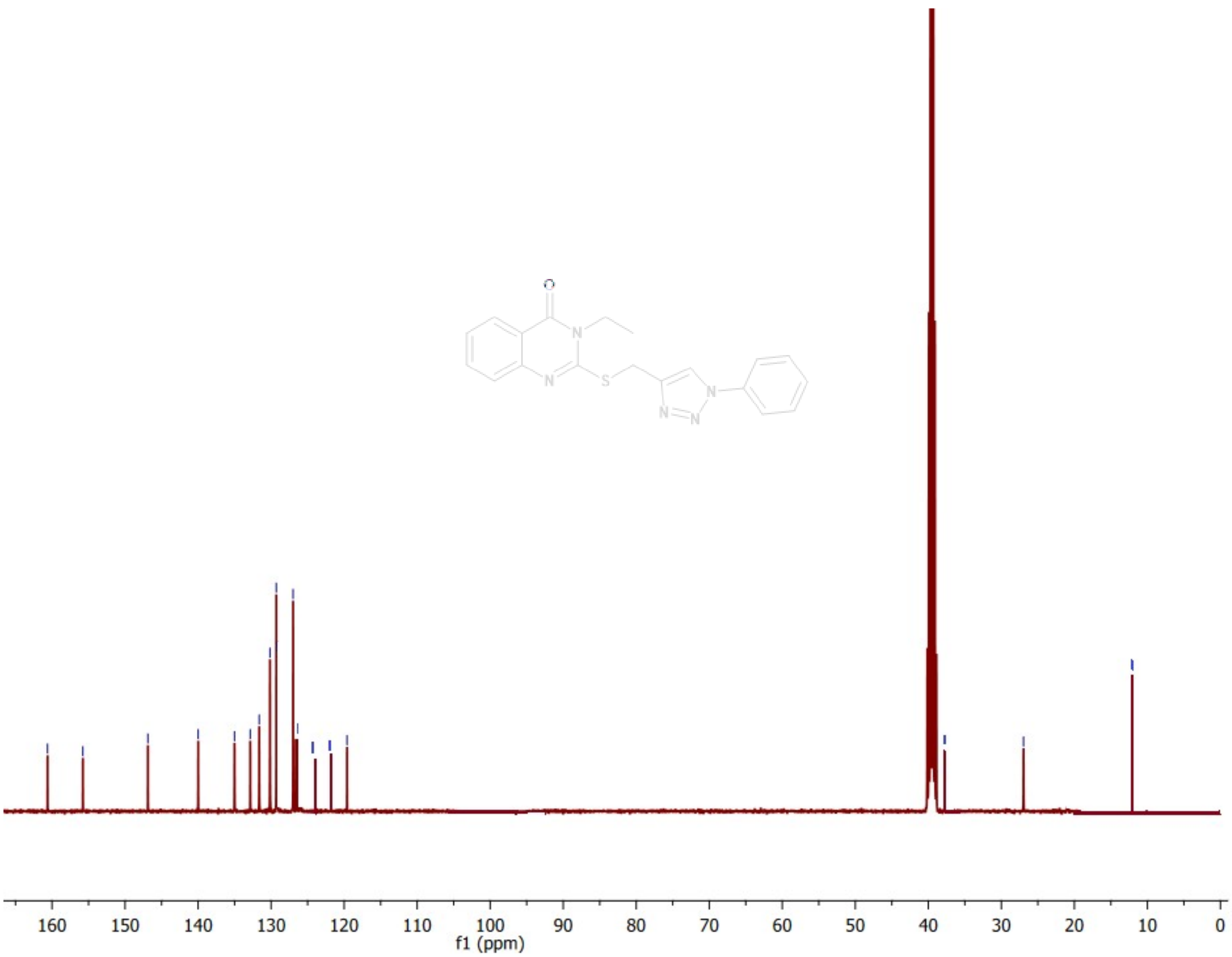

Figure S51: ^{13}C NMR spectrum (100 MHz, DMSO-d_6) of compound **8m**

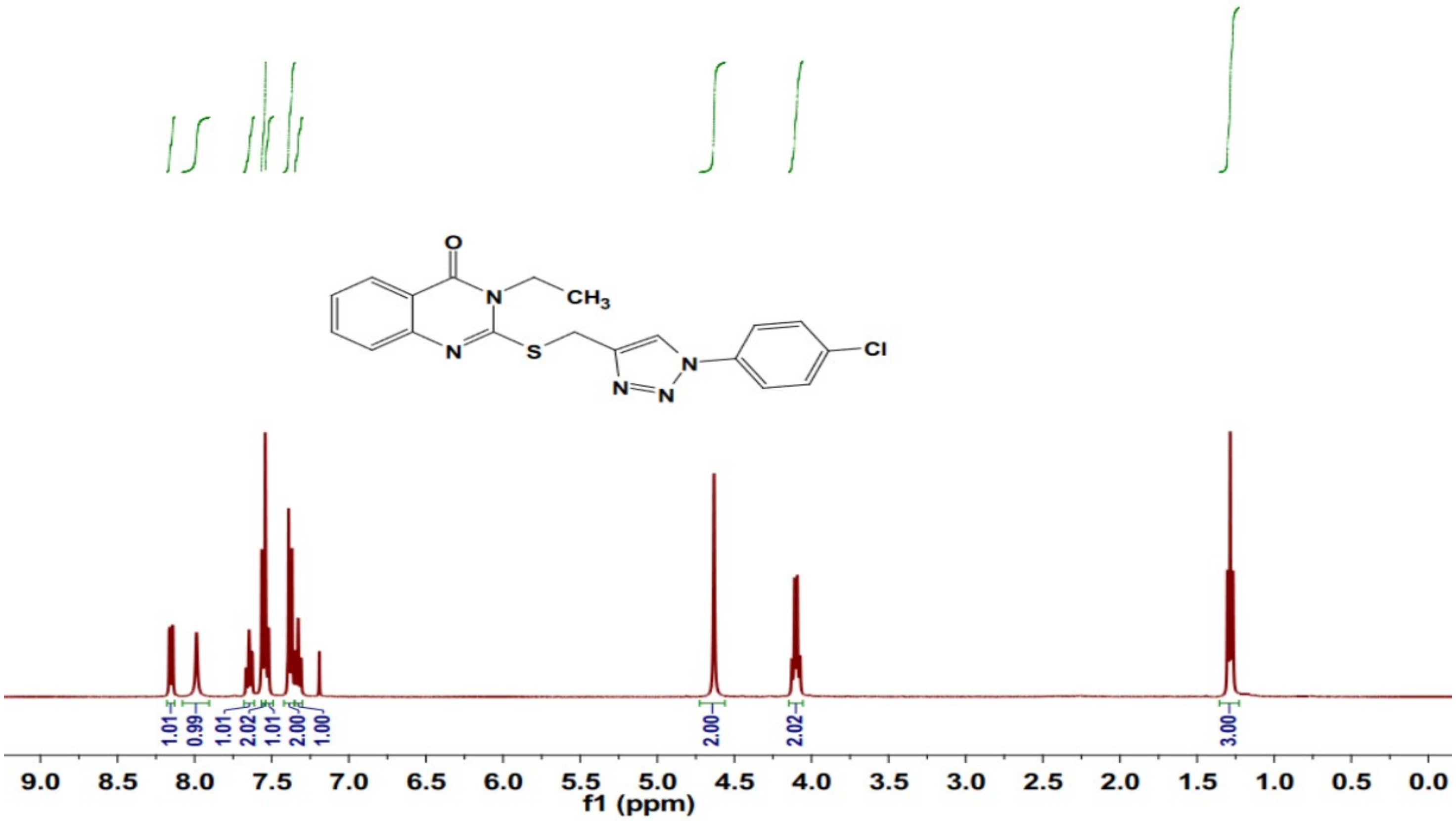

Figure S52: ^{1}H NMR spectrum (400 MHz, $CDCl_3$) of compound **8n**

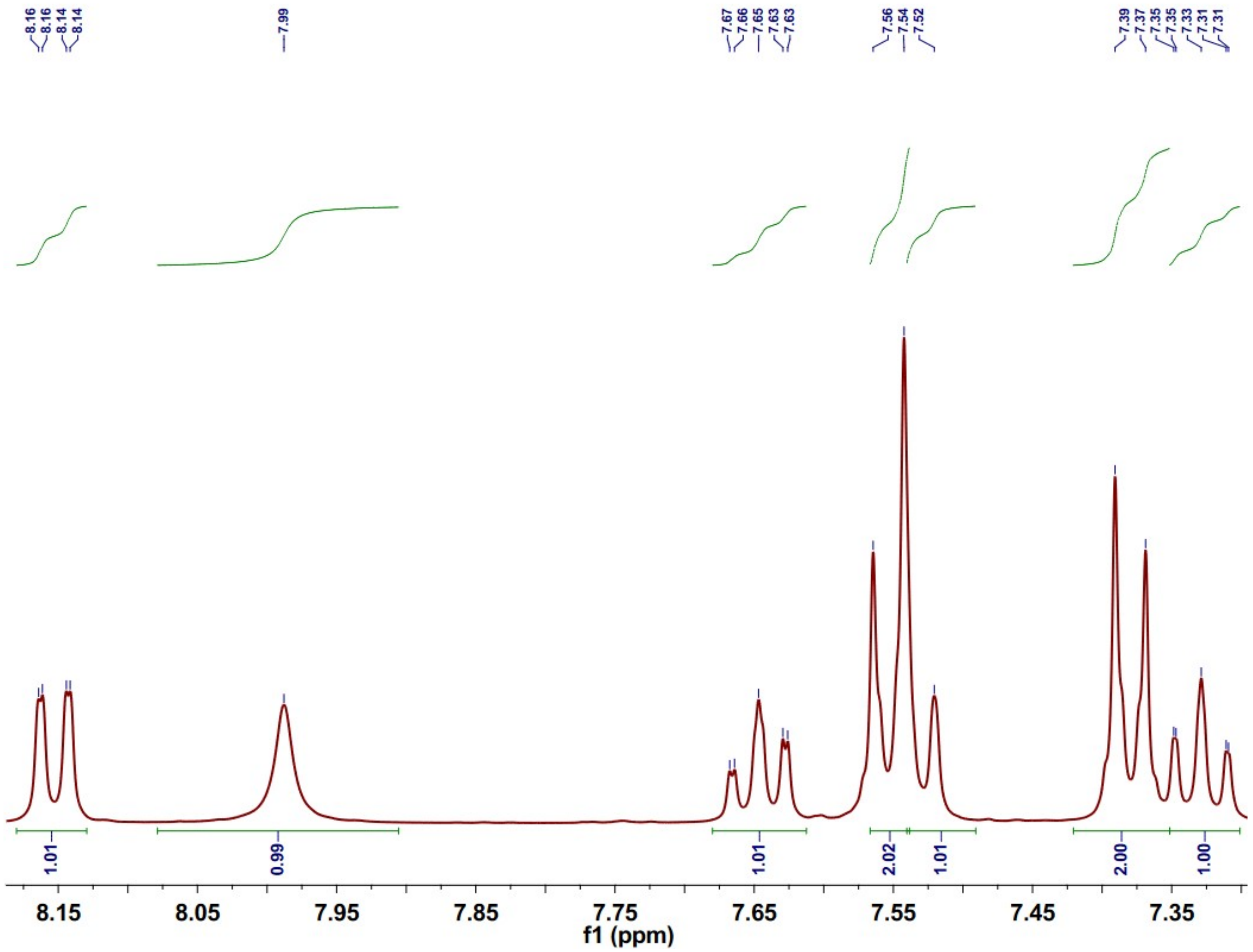

Figure S53: Expanded aromatic region of ^{1}H NMR spectrum (400 MHz, $CDCl_3$) of compound **8n**

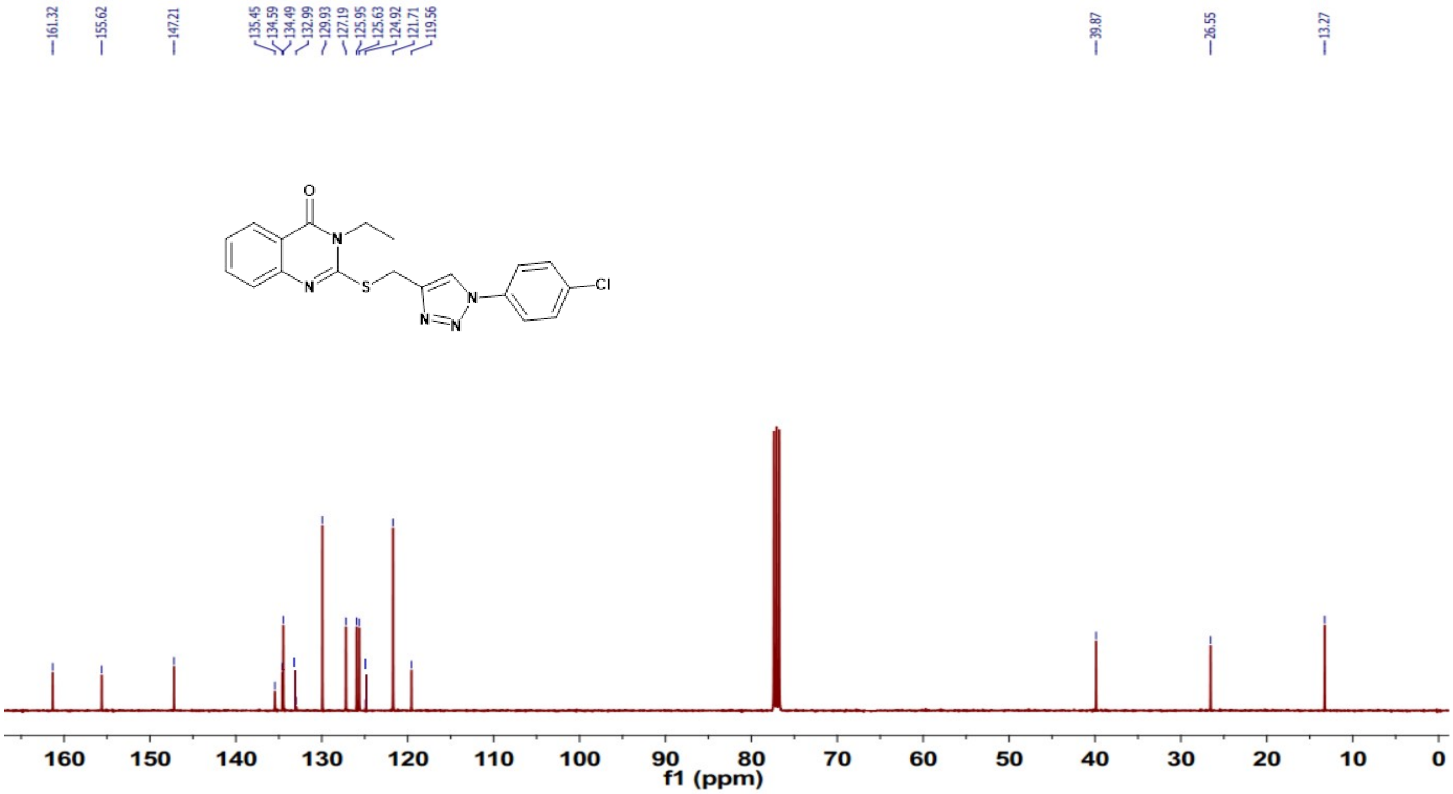

Figure S54: ^{13}C NMR spectrum (100 MHz, $CDCl_3$) of compound **8n**

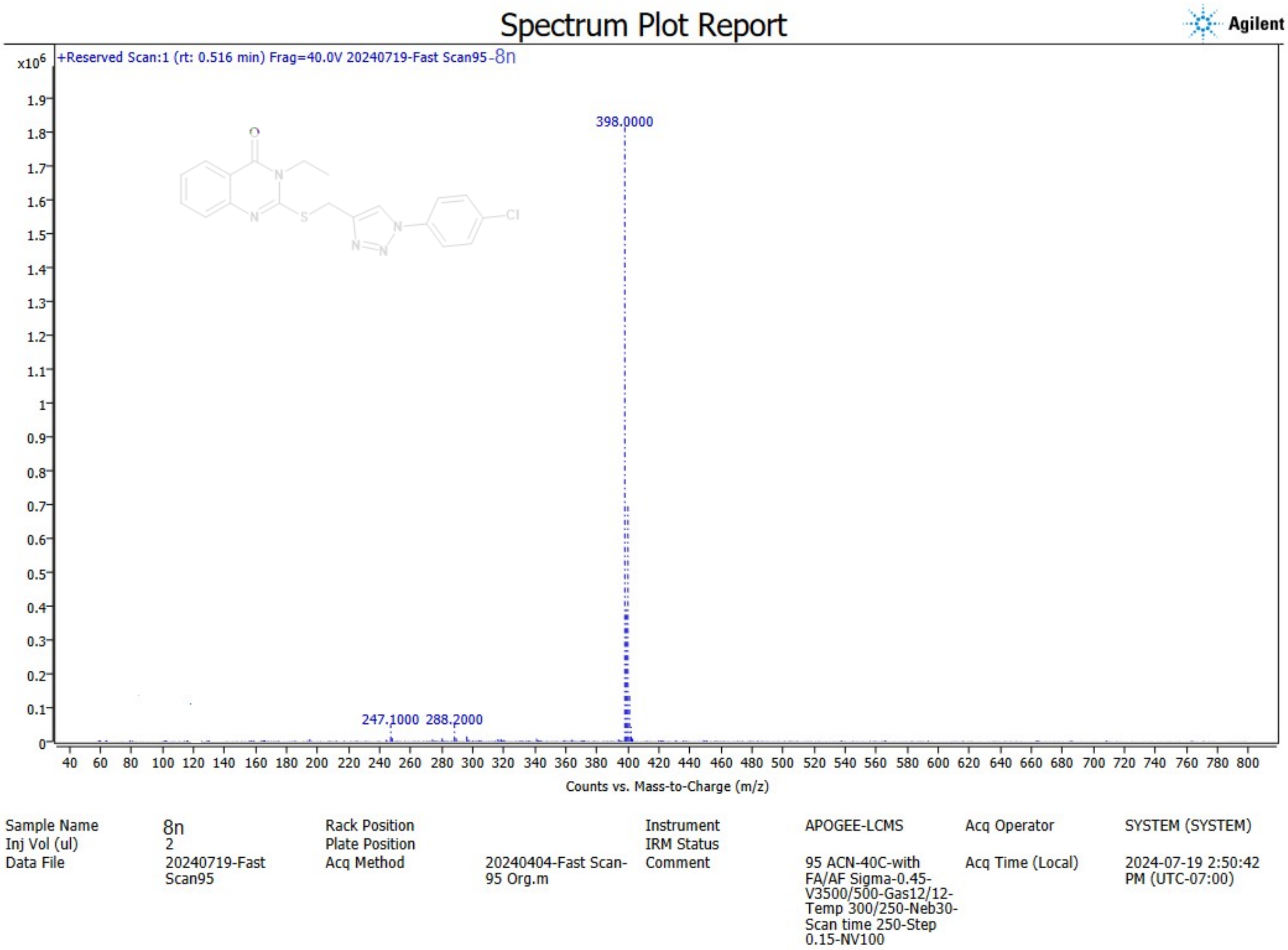

Figure S55: ESI-Mass spectrum of compound **8n**

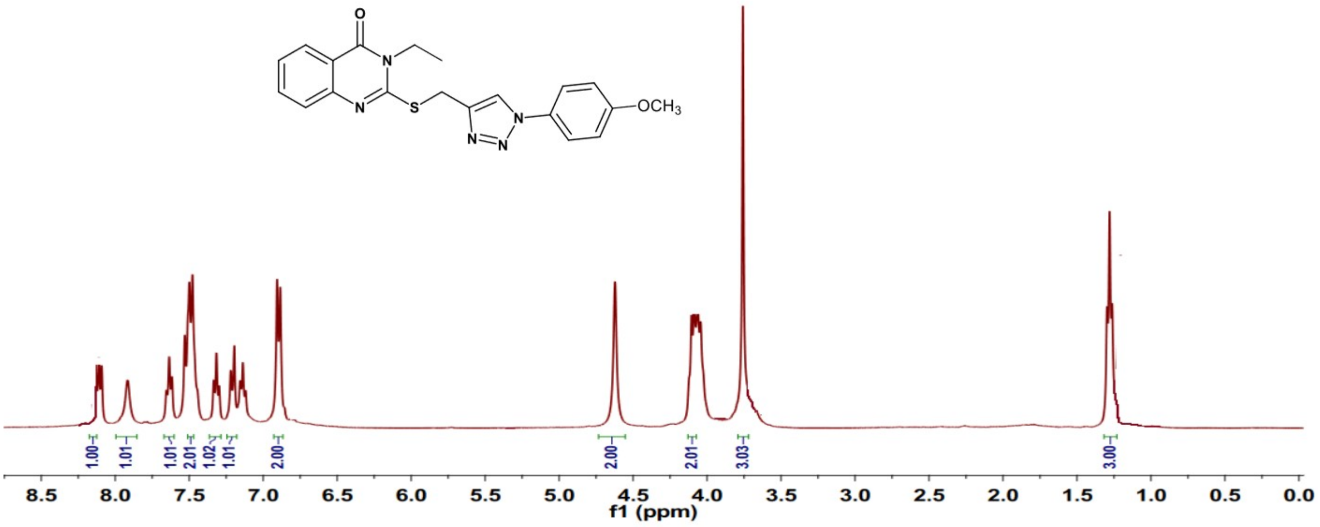

Figure S56: ^{1}H NMR spectrum (400 MHz, $CDCl_3$) of compound **8o**

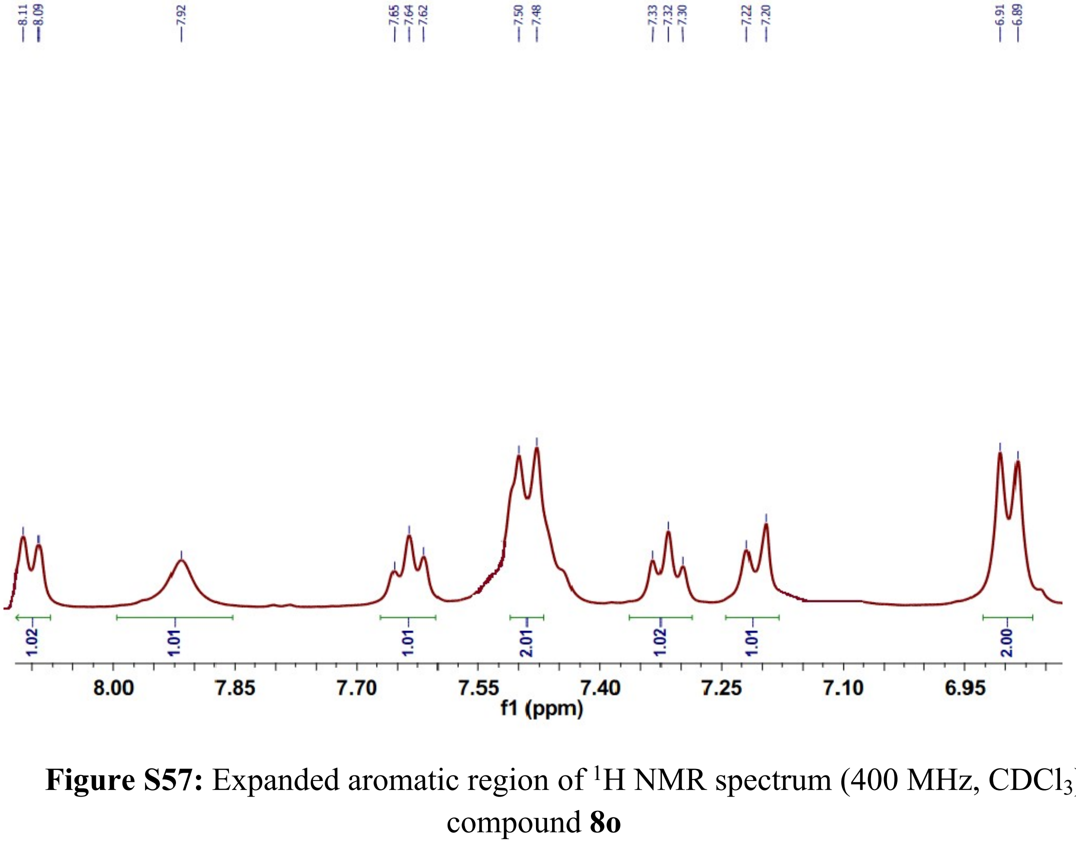

Figure S57: Expanded aromatic region of ^{1}H NMR spectrum (400 MHz, $CDCl_3$) of compound **8o**

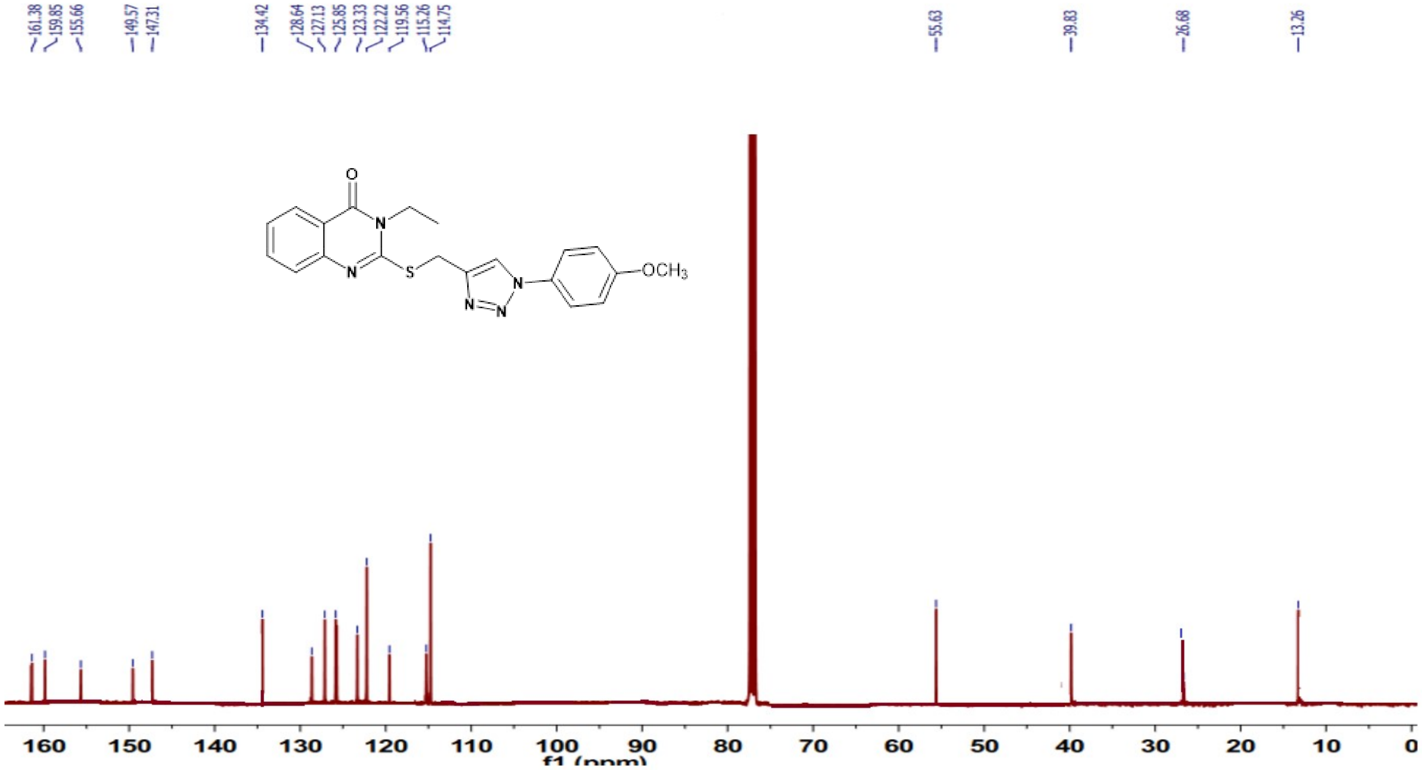

Figure S58: ^{13}C NMR spectrum (100 MHz, $CDCl_3$) of compound **8o**

Spectrum Plot Report

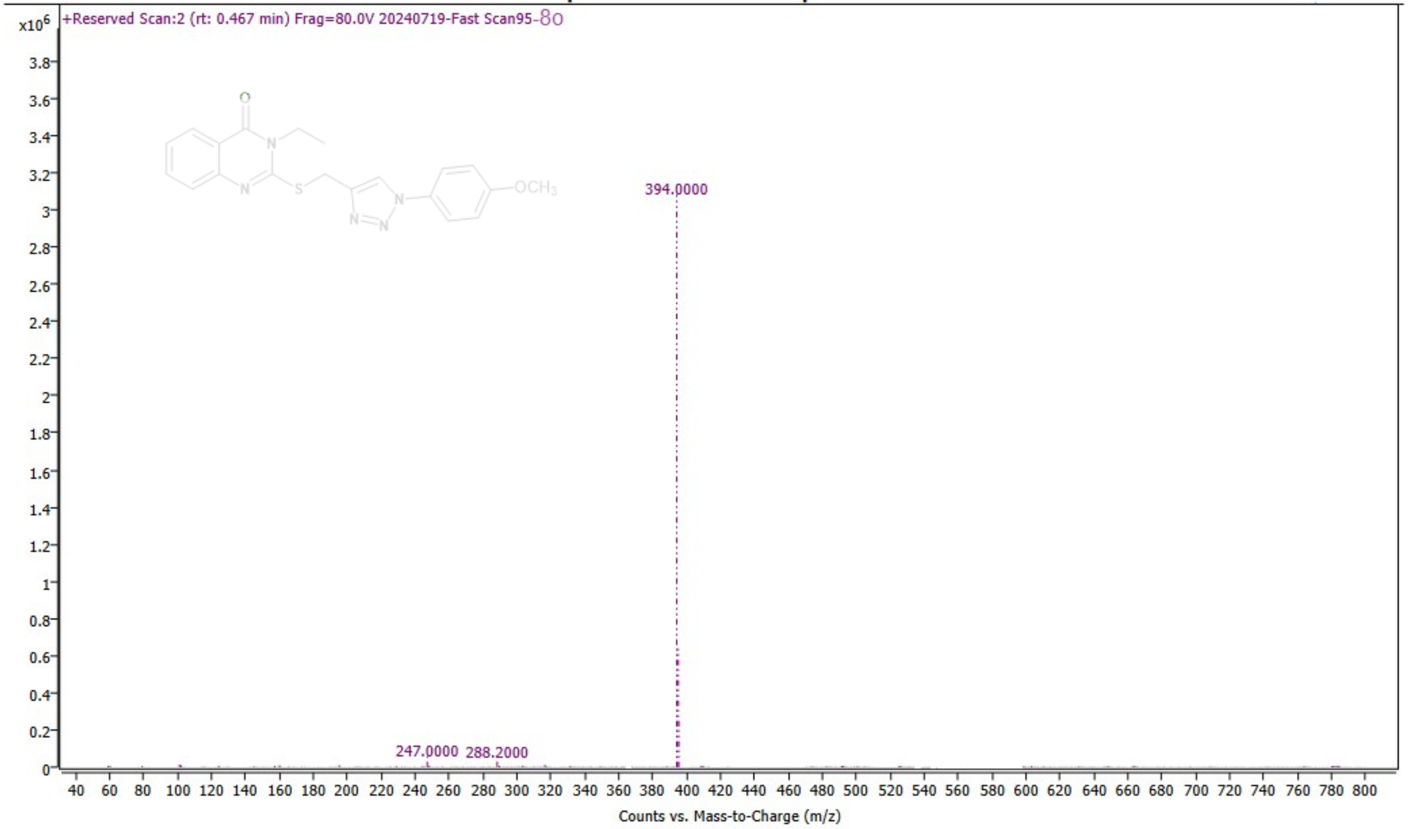

Sample Name	8o	Rack Position		Instrument	APOGEE-LCMS	Acq Operator	SYSTEM (SYSTEM)
Inj Vol (ul)	2	Plate Position		IRM Status			
Data File	20240719-Fast Scan95	Acq Method	20240404-Fast Scan-95 Org.m	Comment	95 ACN-40C-with FA/AF Sigma-0.45-V3500/500-Gas12/12-Temp 300/250-Neb30-Scan time 250-Step 0.15-NV100	Acq Time (Local)	2024-07-19 2:28:48 PM (UTC-07:00)

Figure S59: ESI-Mass spectrum of compound **8o**

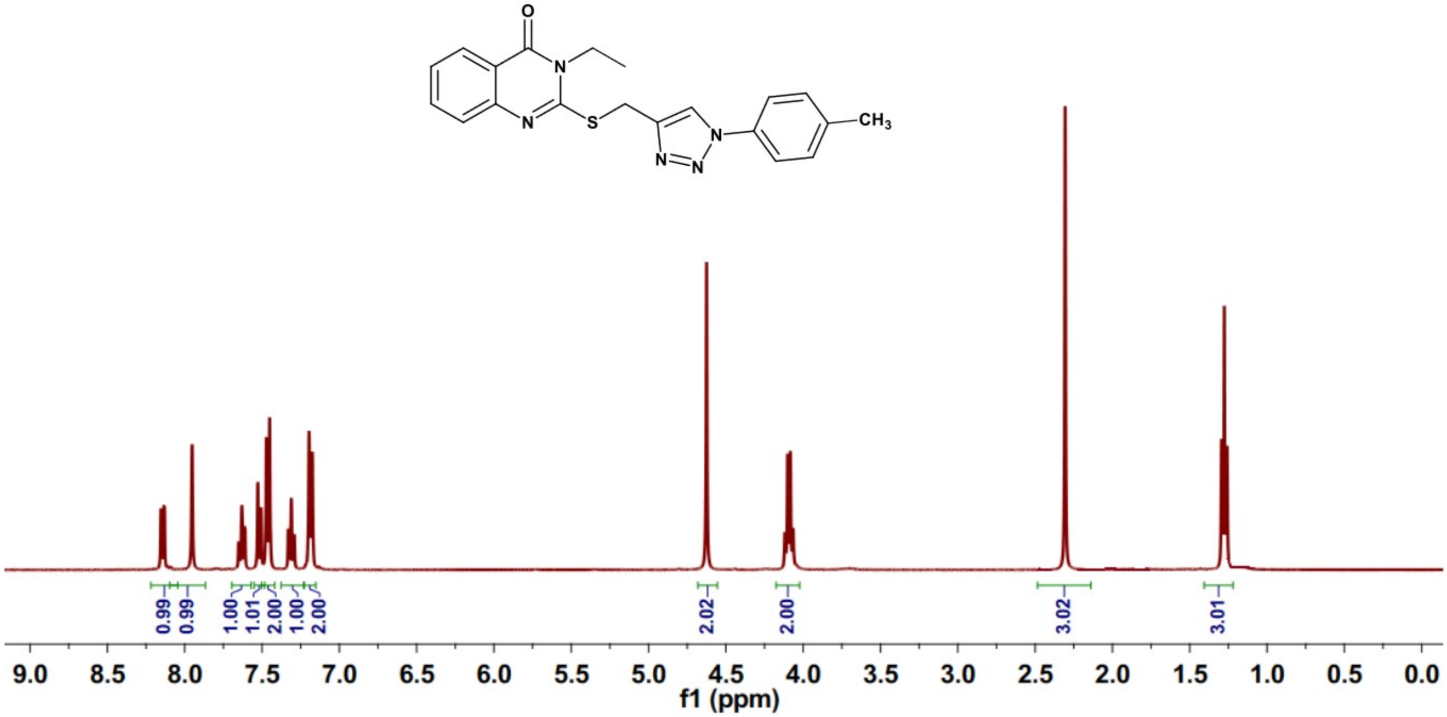

Figure S60: ^{1}H NMR spectrum (400 MHz, $CDCl_3$) of compound **8p**

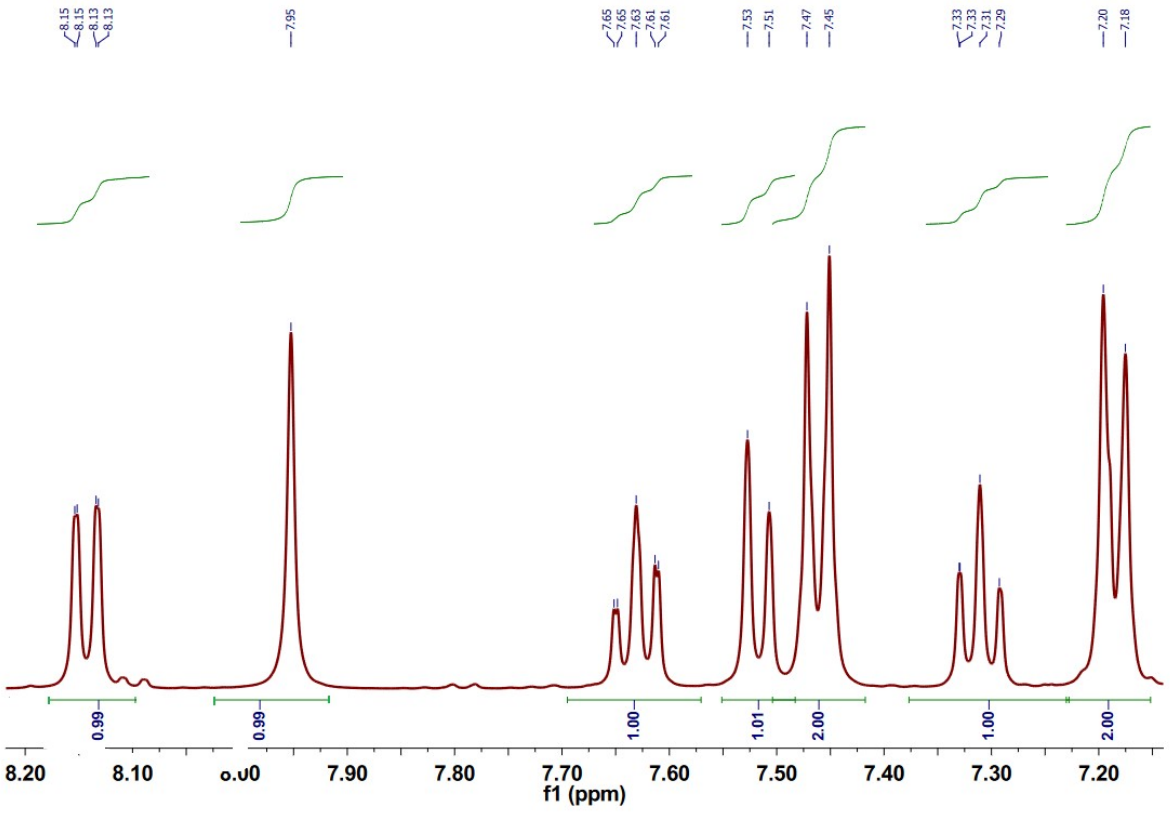

Figure S61: Expanded aromatic region of ^{1}H NMR spectrum (400 MHz, $CDCl_3$) of compound **8p**

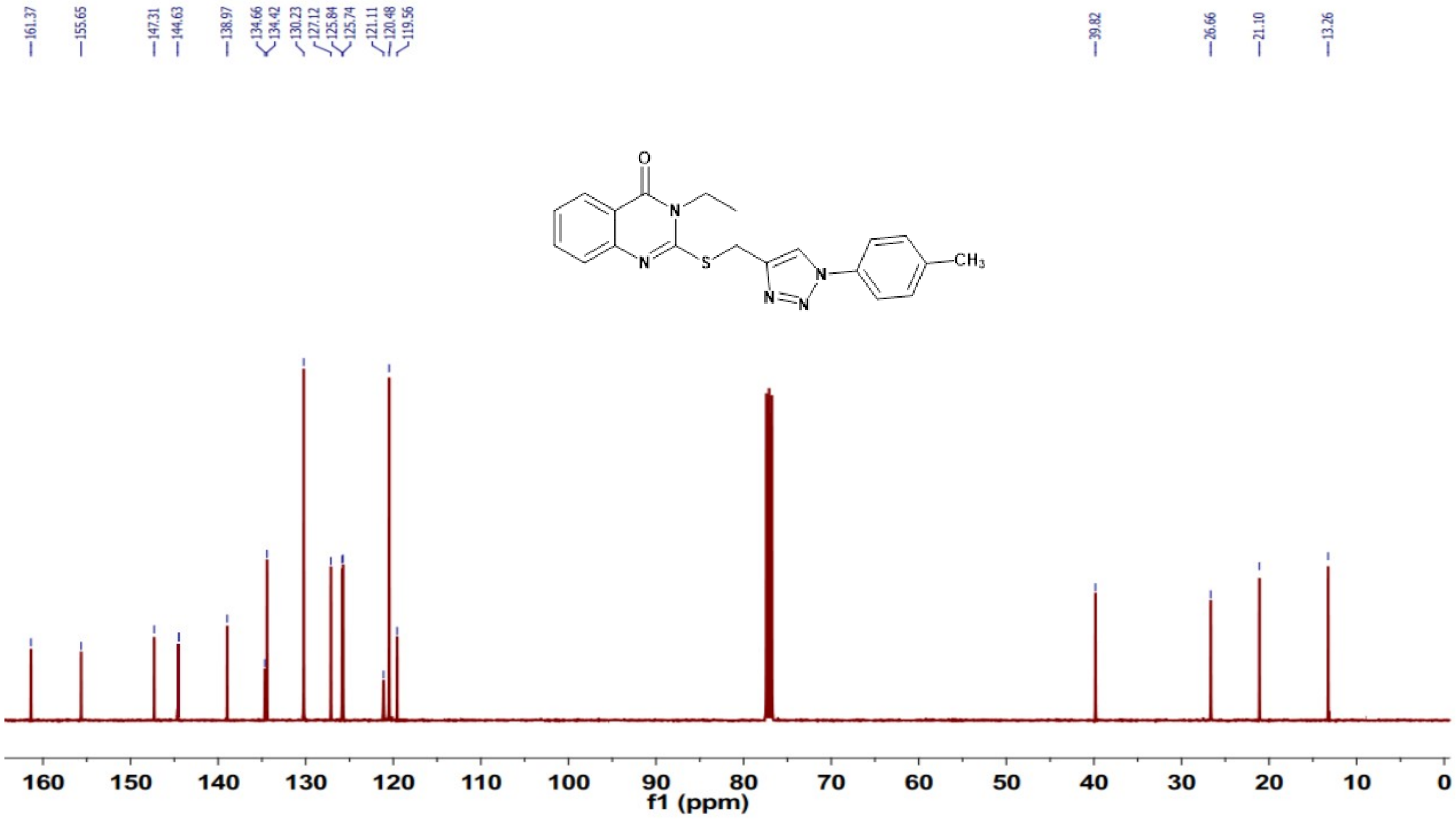

Figure S62: ^{13}C NMR spectrum (100 MHz, $CDCl_3$) of compound **8p**

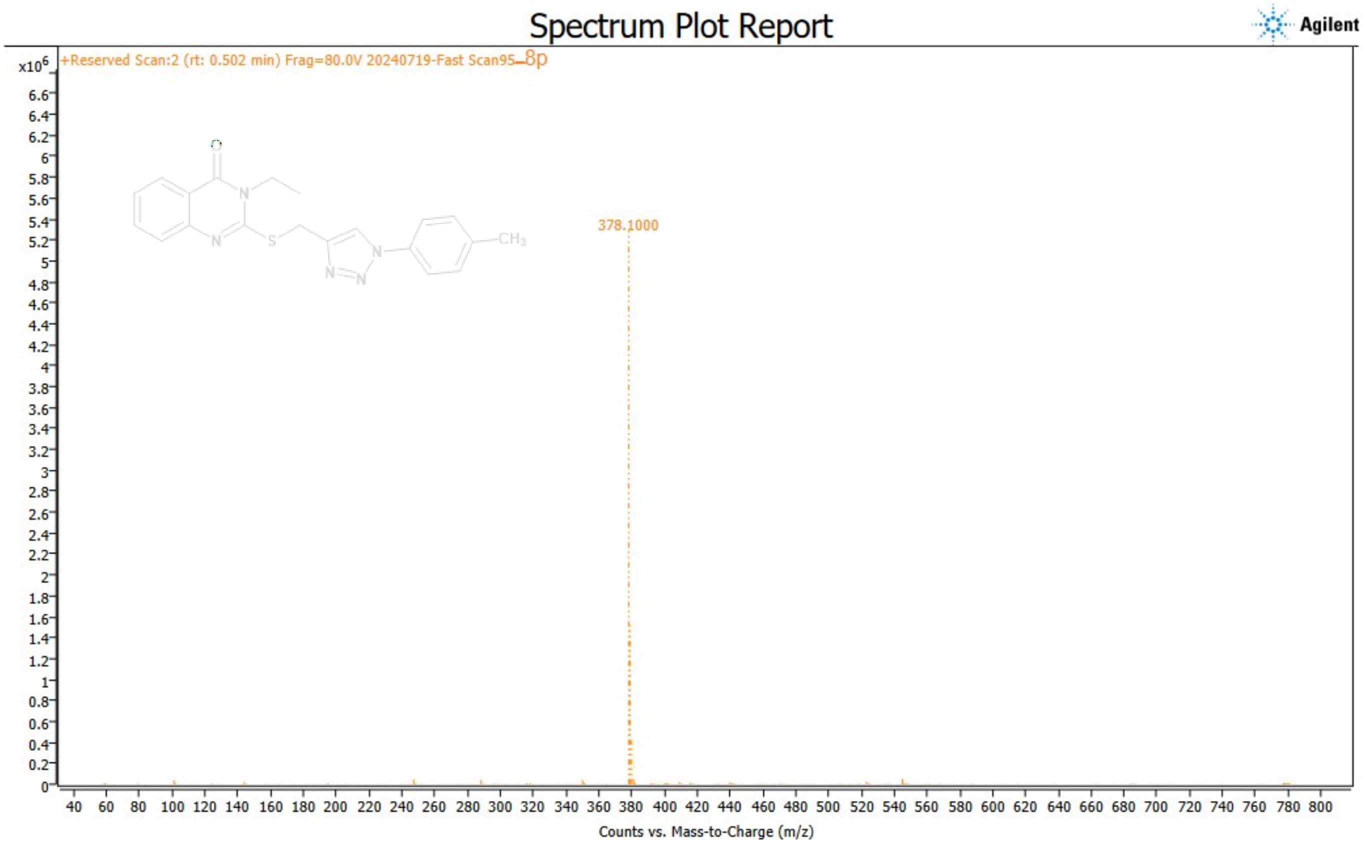

Sample Name	8p	Rack Position		Instrument	APOGEE-LCMS	Acq Operator	SYSTEM (SYSTEM)
Inj Vol (ul)	2	Plate Position		IRM Status			
Data File	20240719-Fast Scan95	Acq Method	20240404-Fast Scan-95 Org.m	Comment	95 ACN-40C-with FA/AF Sigma-0.45-V3500/500-Gas12/12-Temp 300/250-Neb30-Scan time 250-Step 0.15-NV100	Acq Time (Local)	2024-07-19 2:32:32 PM (UTC-07:00)

Figure S63: ESI-Mass spectrum of compound **8p**

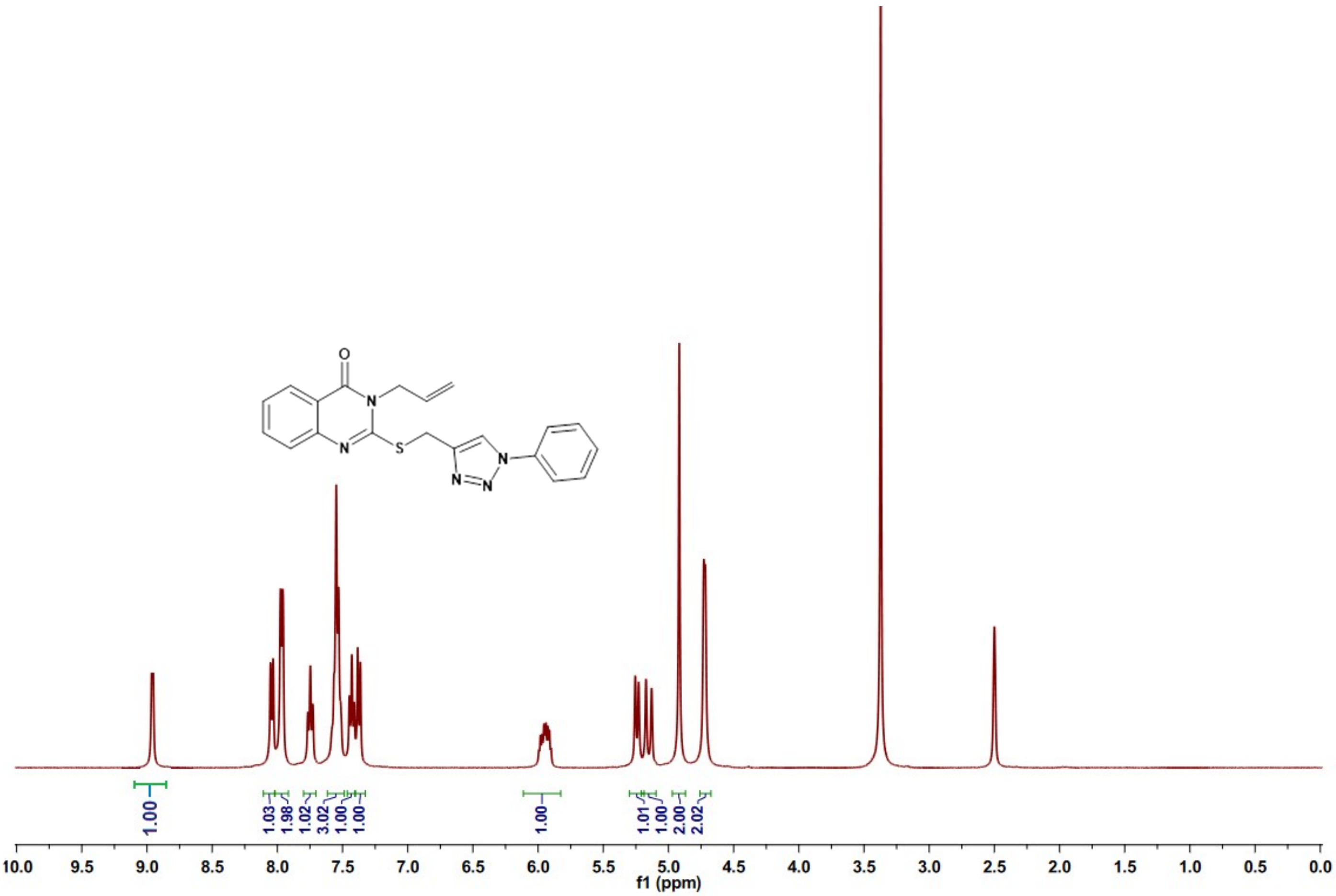

Figure S64: ^{1}H NMR spectrum (400 MHz, DMSO-d_6) of compound **8q**

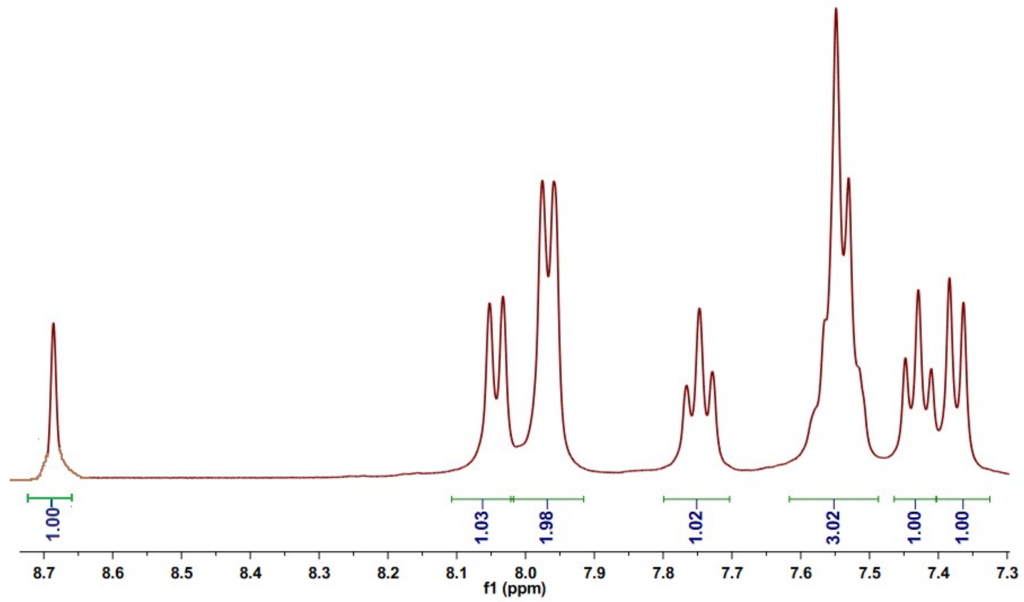

Figure S65: Expanded aromatic region of ^{1}H NMR spectrum (400 MHz, DMSO-d_6) of compound **8q**

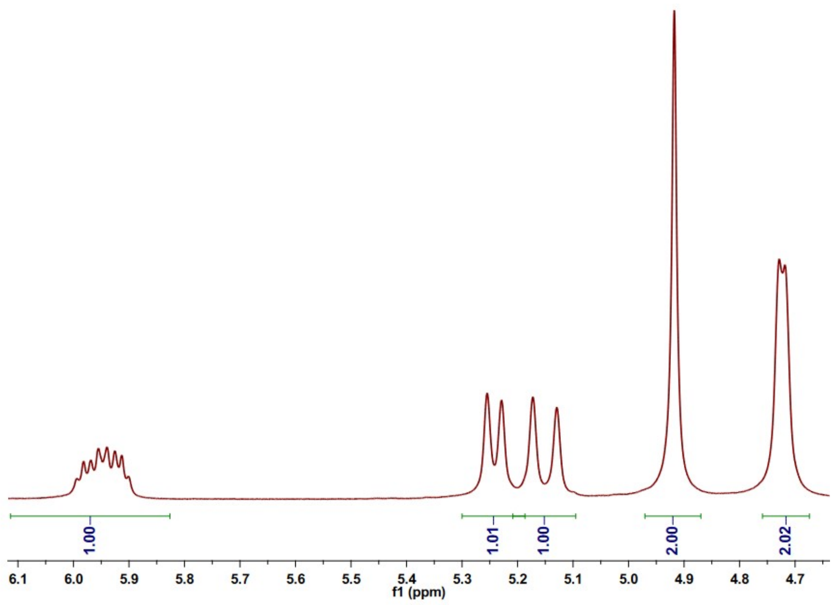

Figure S66: Expanded aliphatic and olefinic region regions of ^{1}H NMR spectrum (400 MHz, DMSO-d_6) of compound **8q**

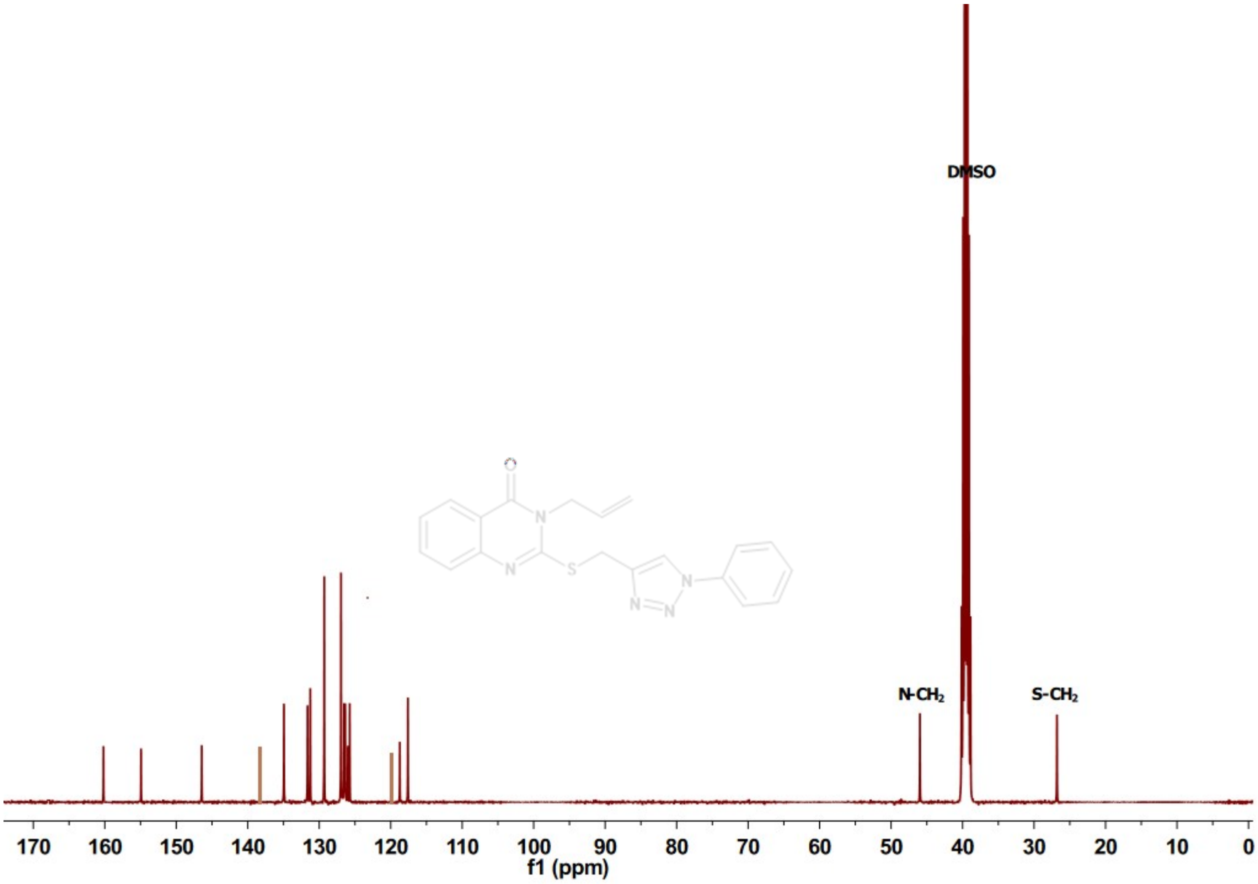

Figure S67: ^{13}C NMR spectrum (100 MHz, DMSO-d_6) of compound **8q**

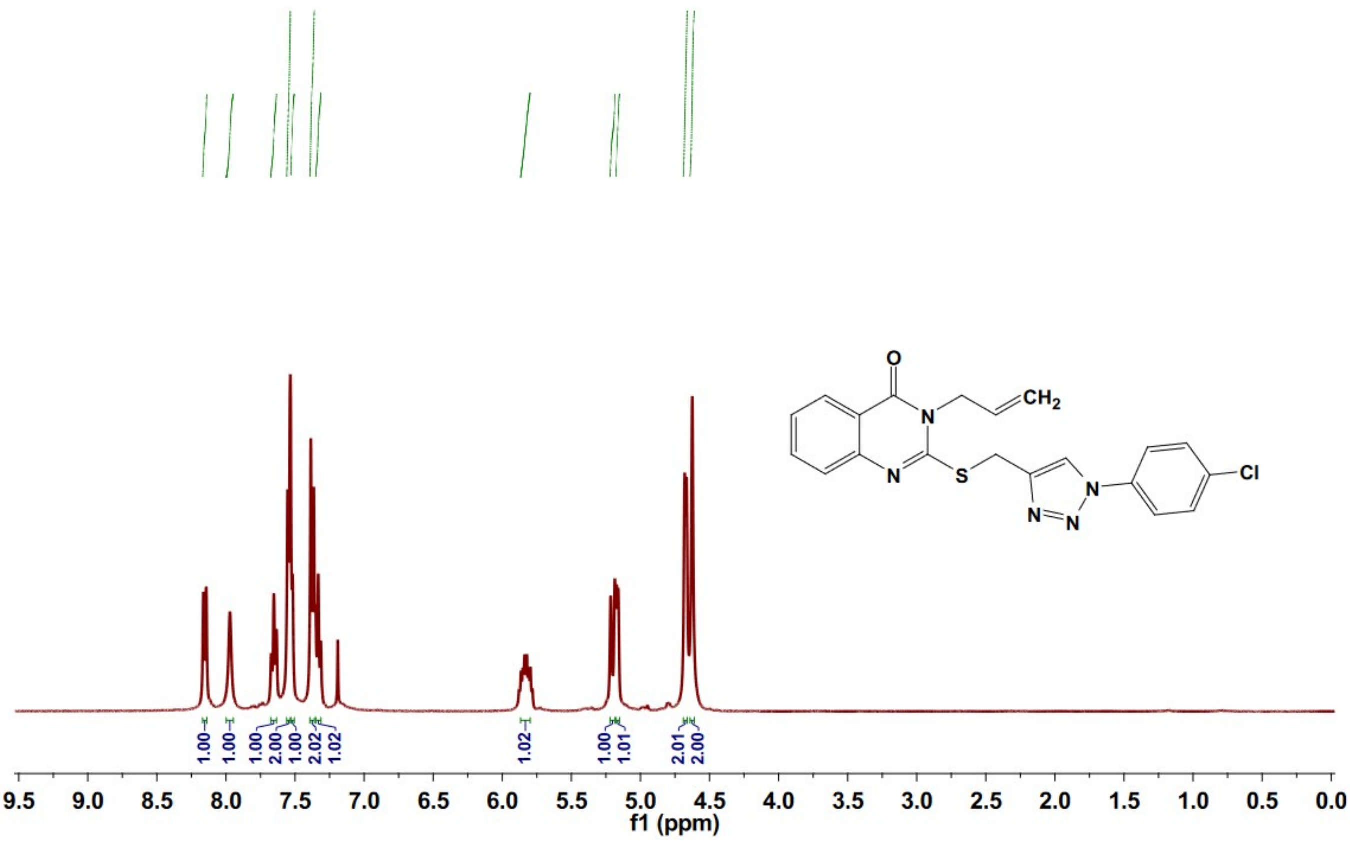

Figure S68: ^{1}H NMR spectrum (400 MHz, $CDCl_3$) of compound **8r**

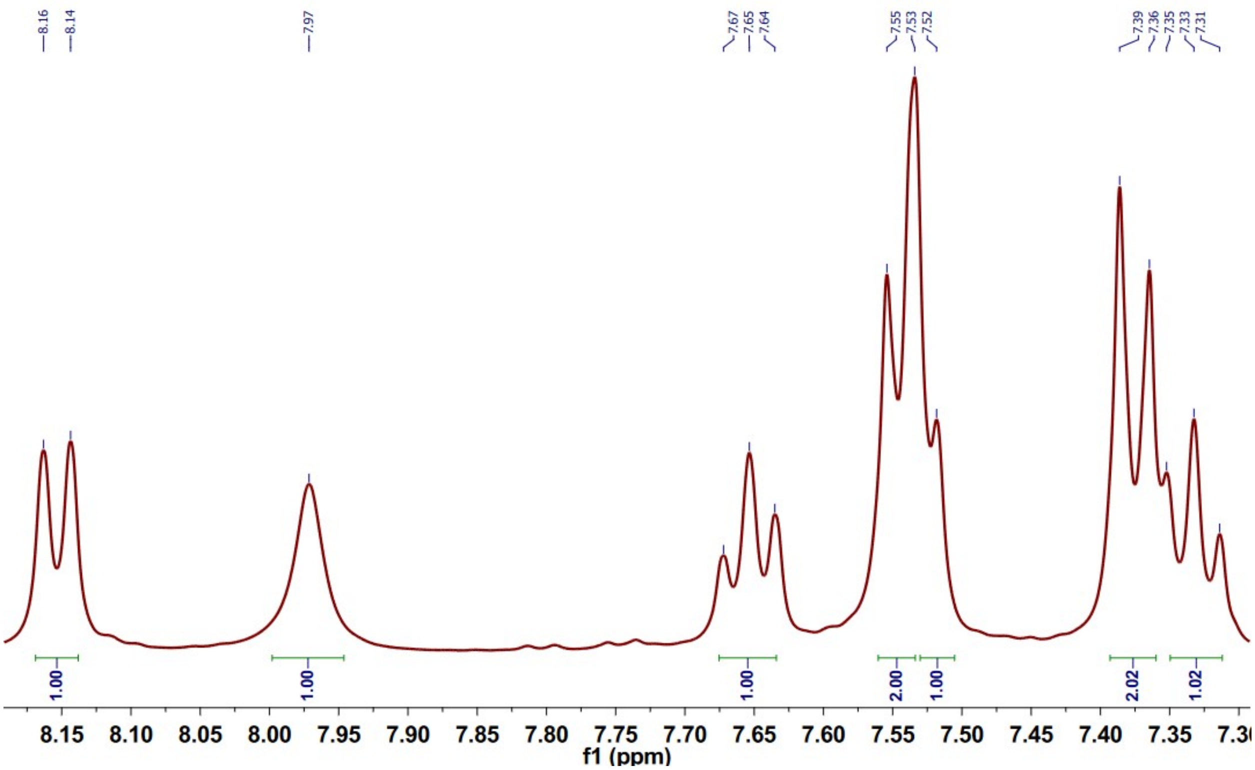

Figure S69: Expanded aromatic region of ^{1}H NMR spectrum (400 MHz, $CDCl_3$) of compound **8r**

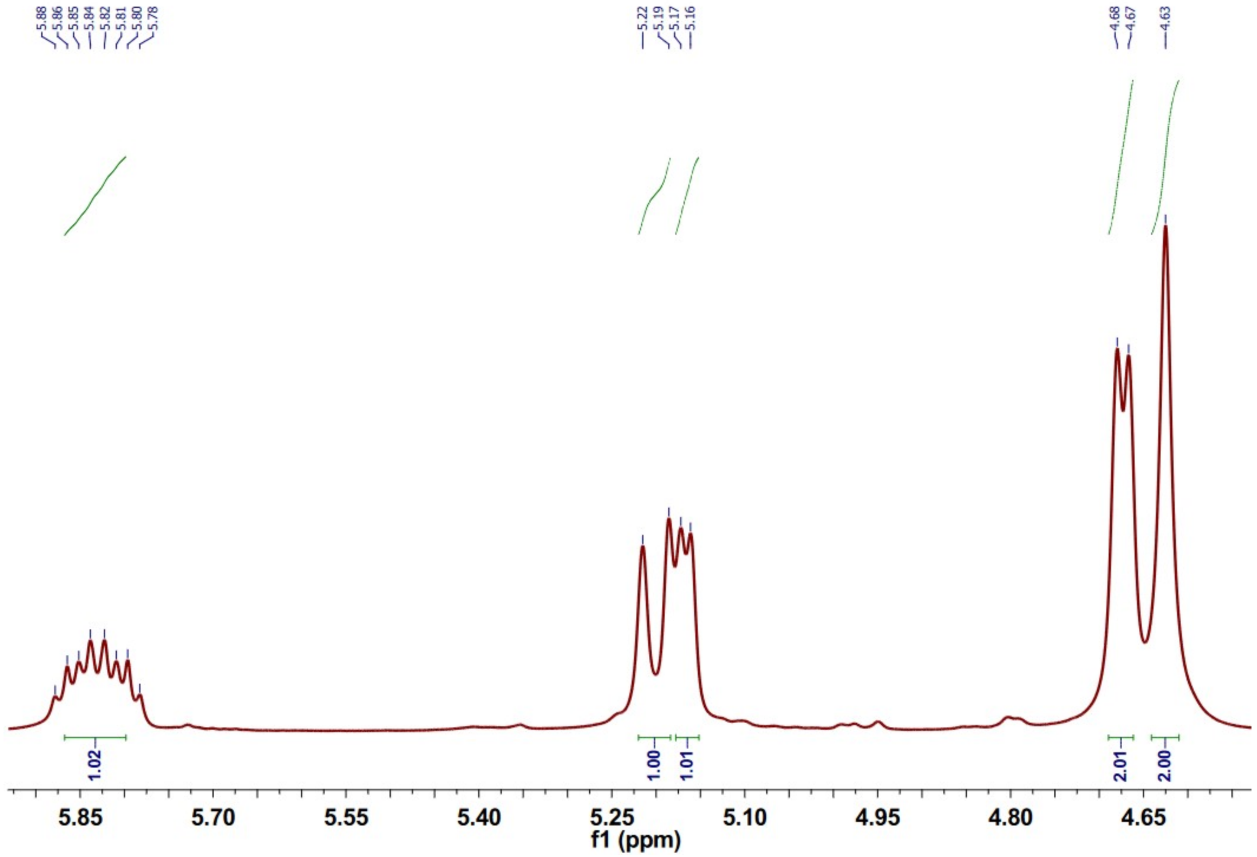

Figure S70: Expanded aliphatic and olefinic region regions of ^{1}H NMR spectrum (400 MHz, $CDCl_3$) of compound **8r**

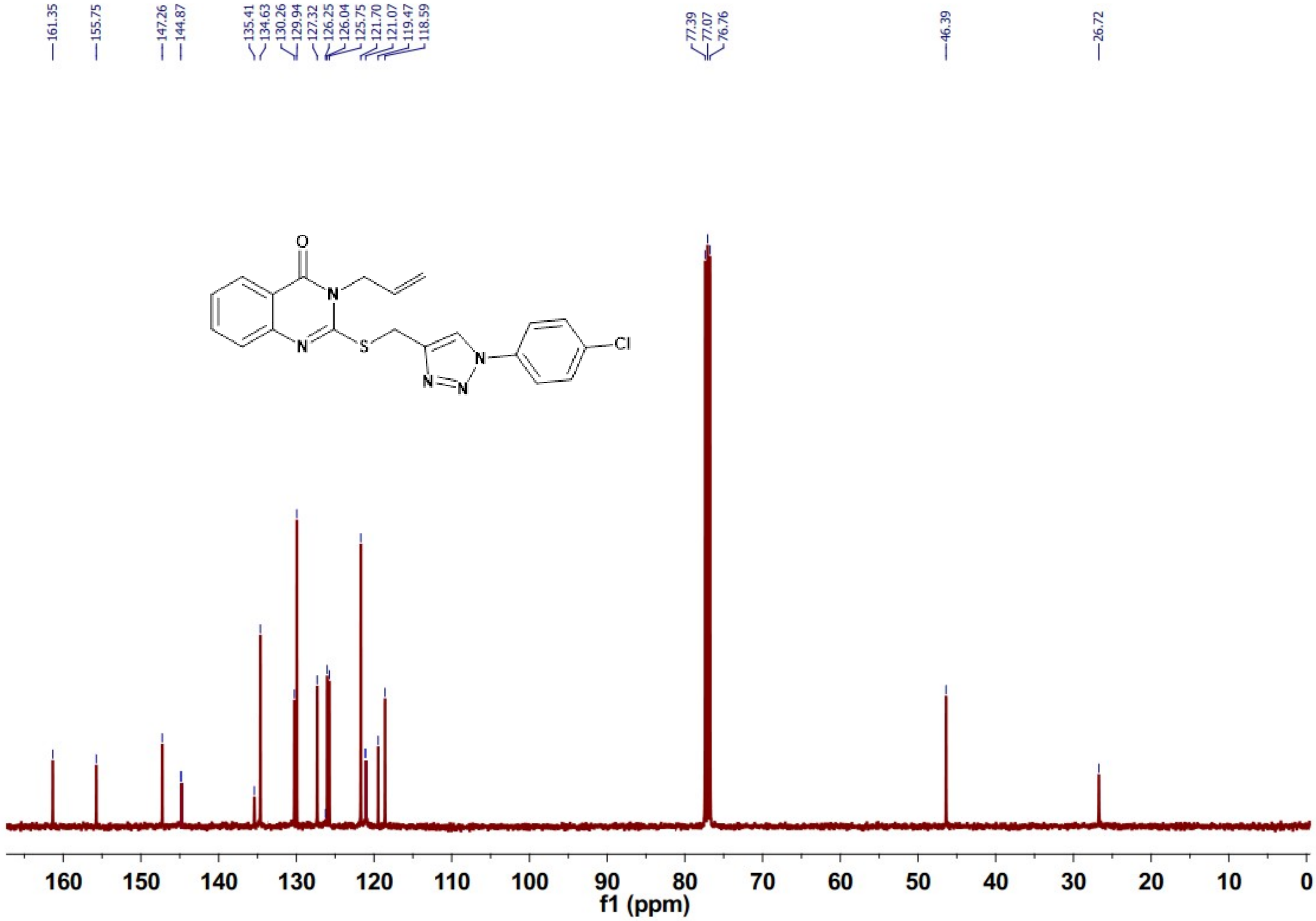

Figure S71: ^{13}C NMR spectrum (100 MHz, $CDCl_3$) of compound **8r**

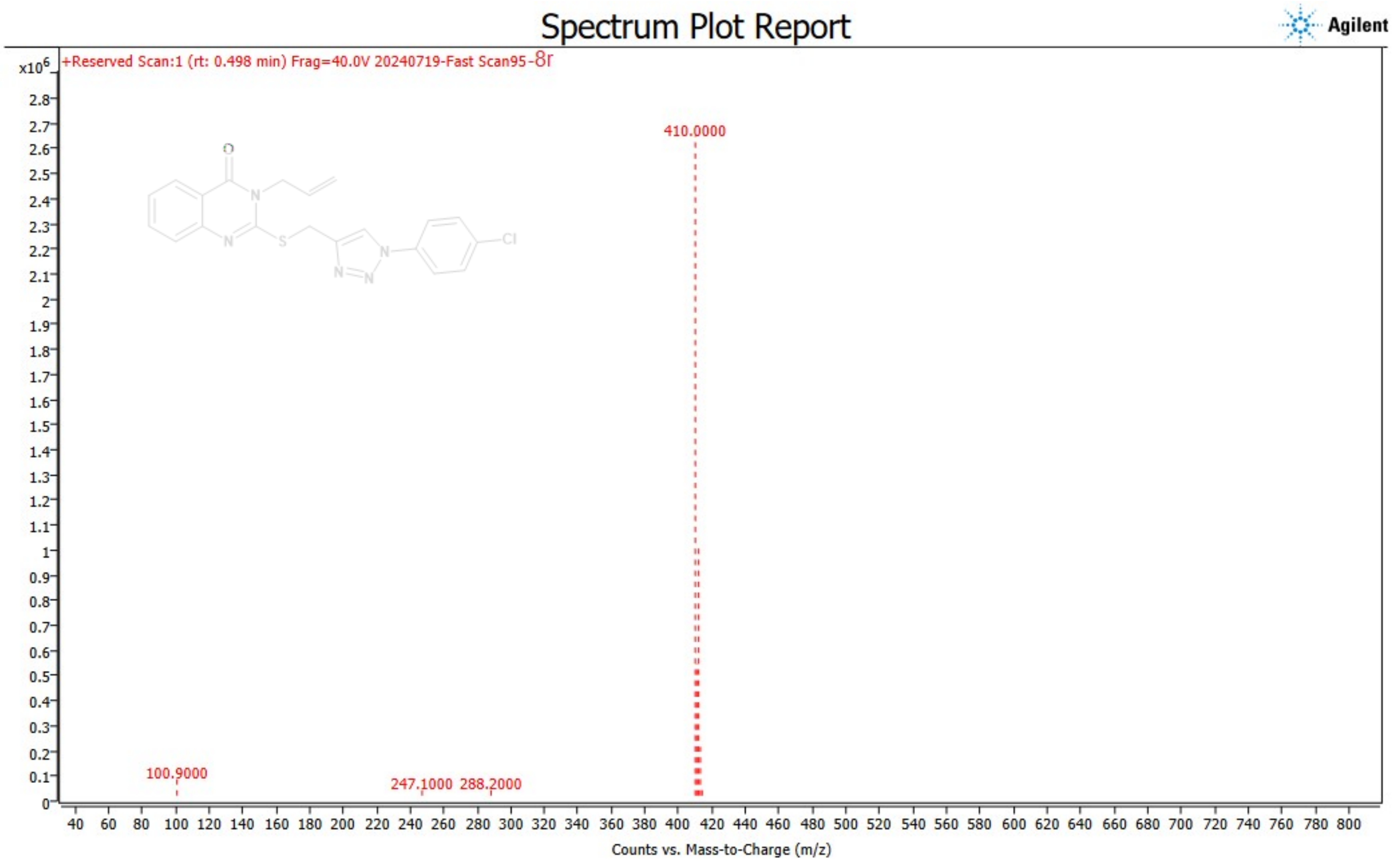

Sample Name	8r	Rack Position		Instrument	APOGEE-LCMS	Acq Operator	SYSTEM (SYSTEM)
Inj Vol (ul)	2	Plate Position		IRM Status			
Data File	20240719-Fast Scan95	Acq Method	20240404-Fast Scan-95 Org.m	Comment	95 ACN-40C-with FA/AF Sigma-0.45-V3500/500-Gas12/12-Temp 300/250-Neb30-Scan time 250-Step 0.15-NV100	Acq Time (Local)	2024-07-19 2:36:15 PM (UTC-07:00)

Figure S72: ESI-Mass spectrum of compound **8r**

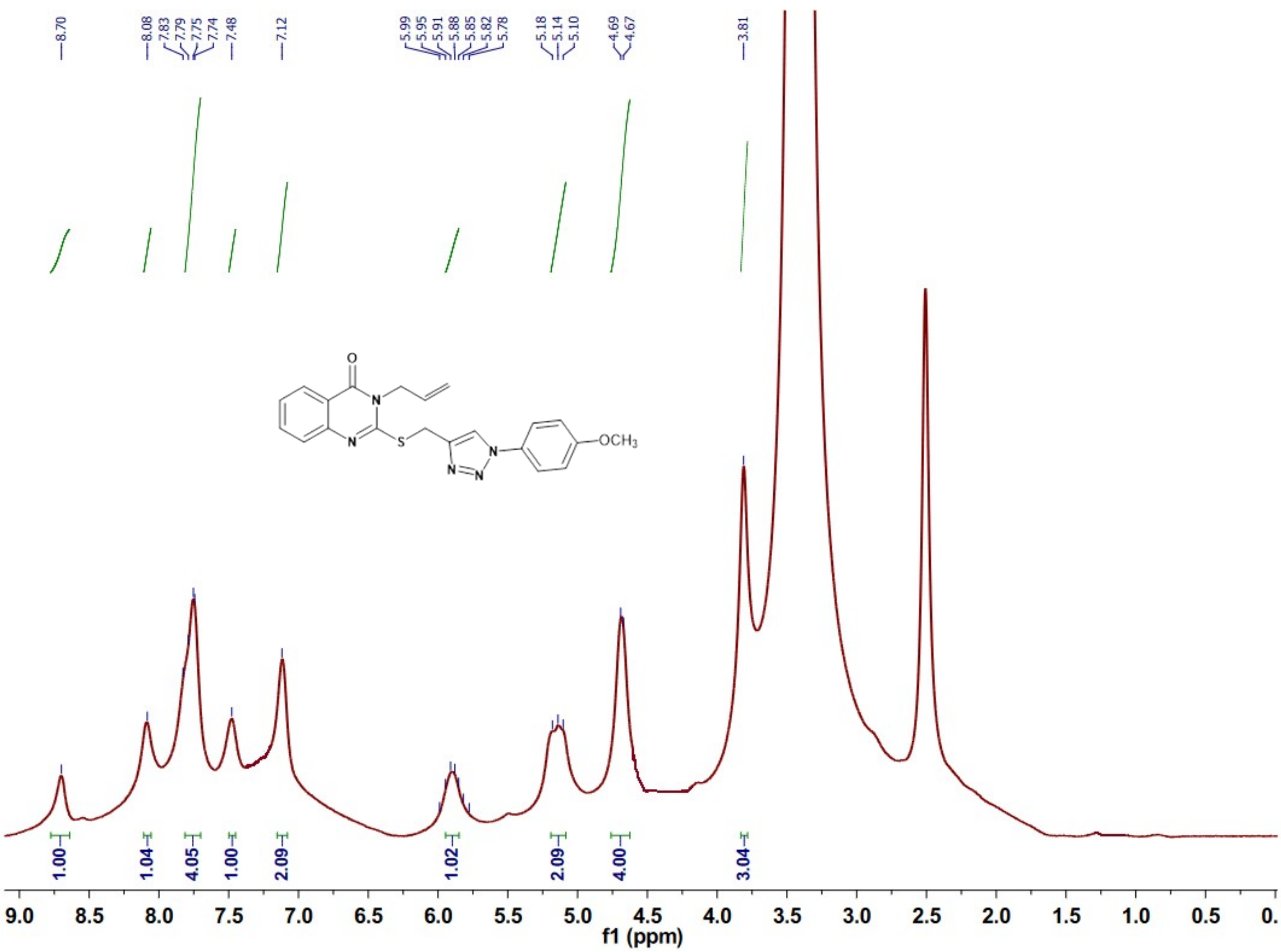

Figure S73: ^{1}H NMR spectrum (400 MHz, DMSO-d_6) of compound **8s**

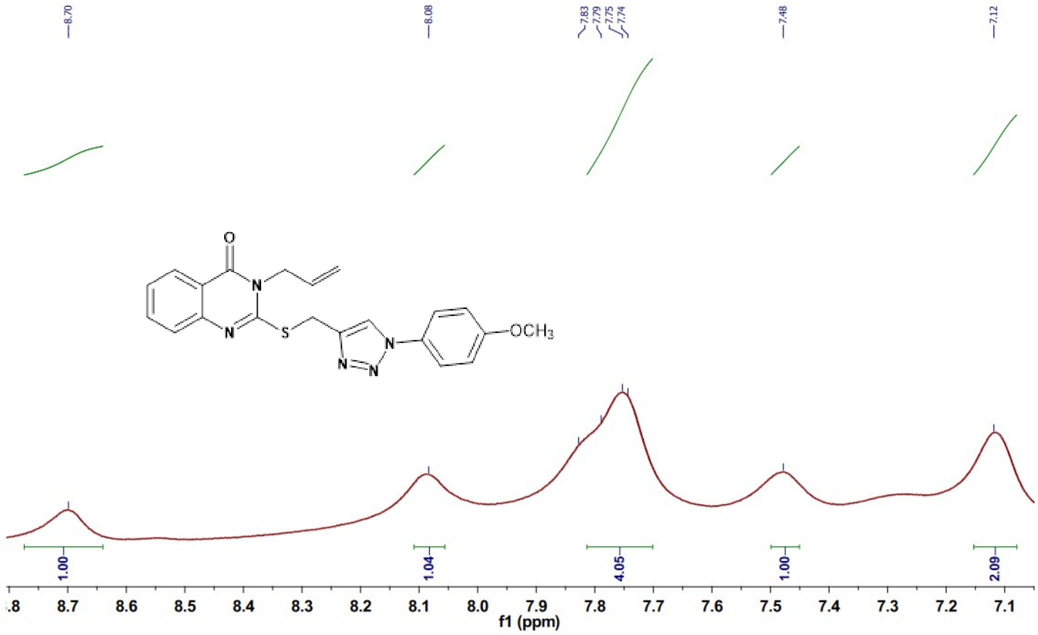

Figure S74: Expanded aromatic region of ^{1}H NMR spectrum (400 MHz, DMSO-d_6) of compound **8s**

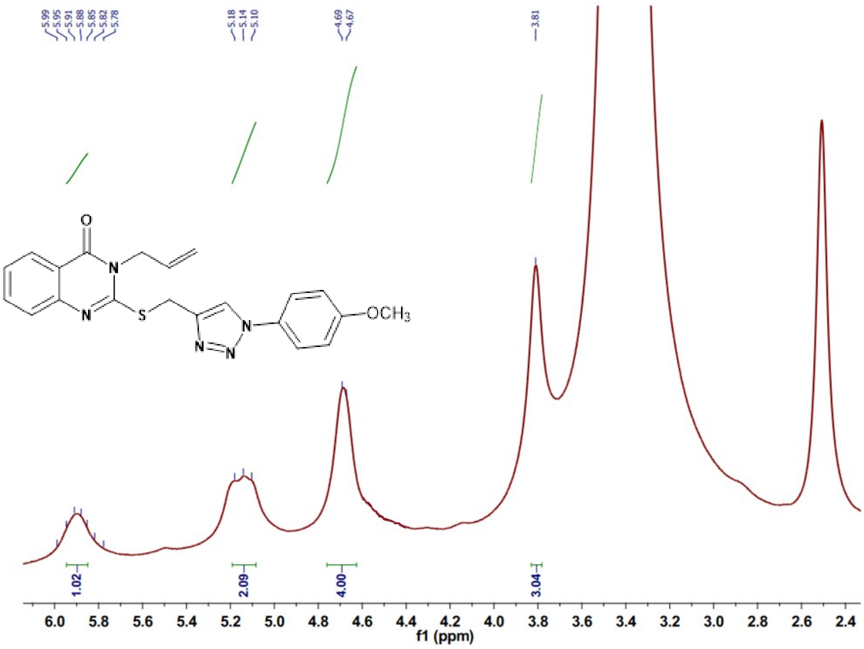

Figure S75: Expanded aliphatic and olefinic region regions of ^{1}H NMR spectrum (400 MHz, DMSO-d_6) of compound **8s**

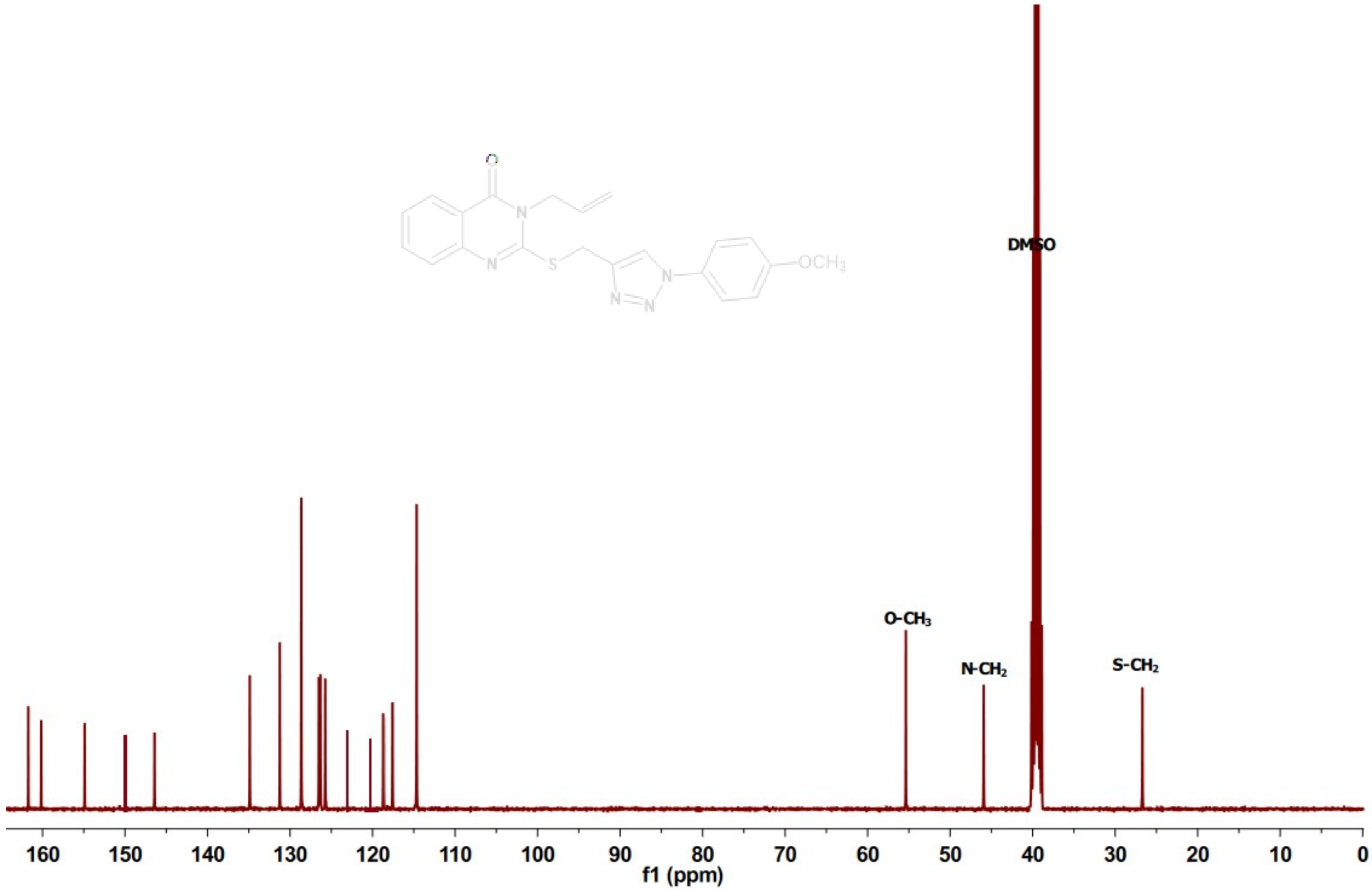

Figure S76: ^{13}C NMR spectrum (100 MHz, DMSO-d_6) of compound **8s**

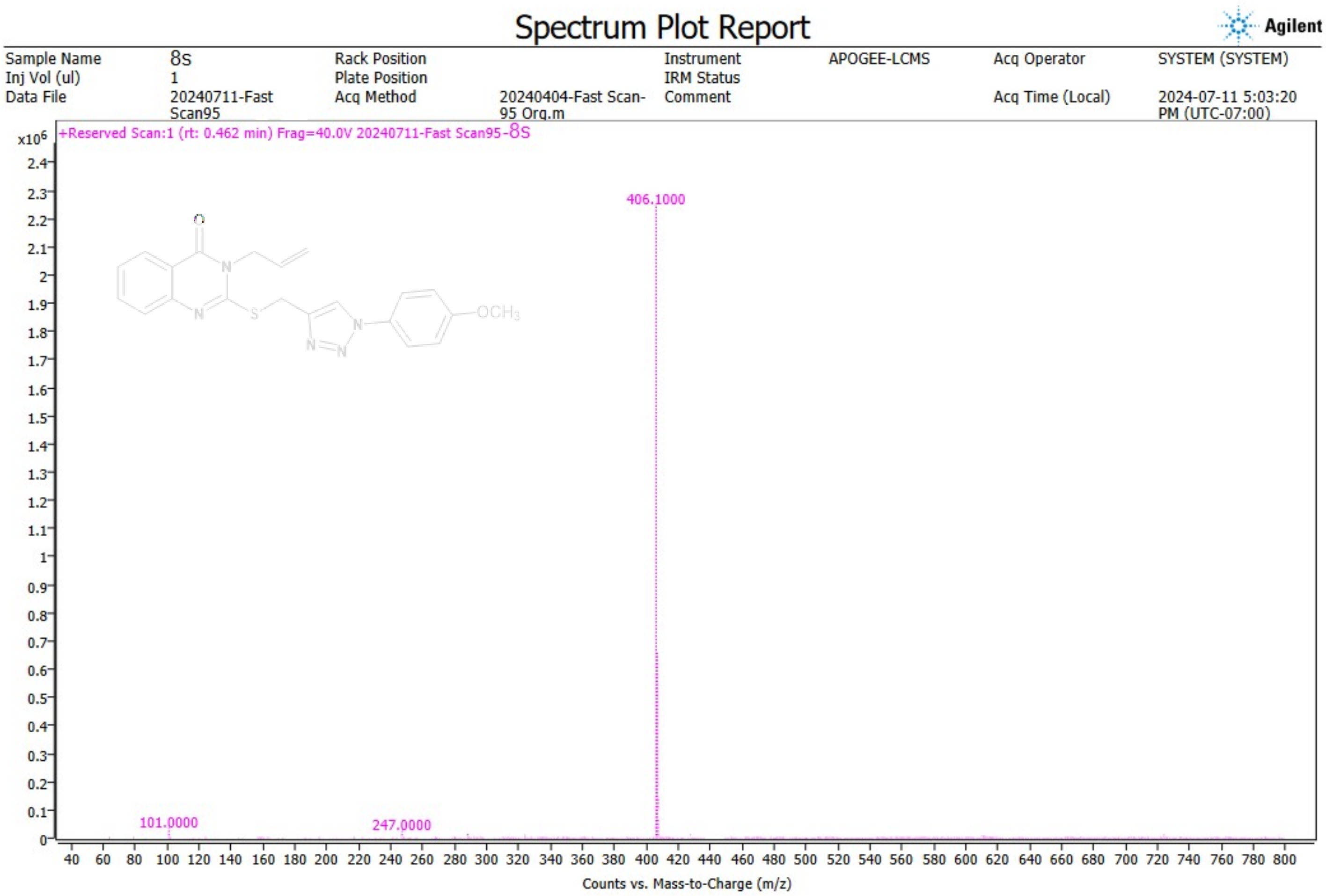

Figure S77: ESI-Mass spectrum of compound **8s**

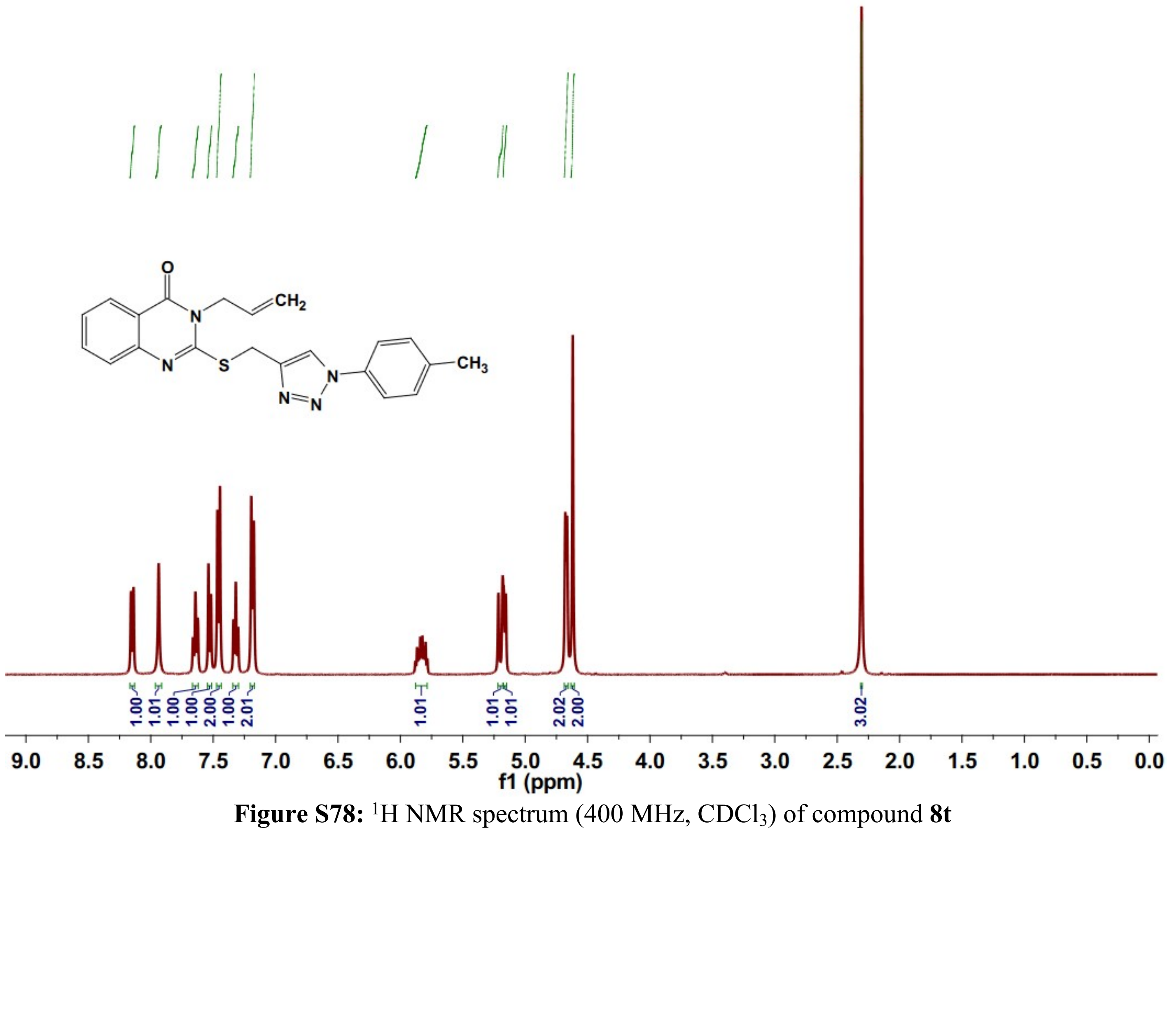

Figure S78: ^{1}H NMR spectrum (400 MHz, $CDCl_3$) of compound **8t**

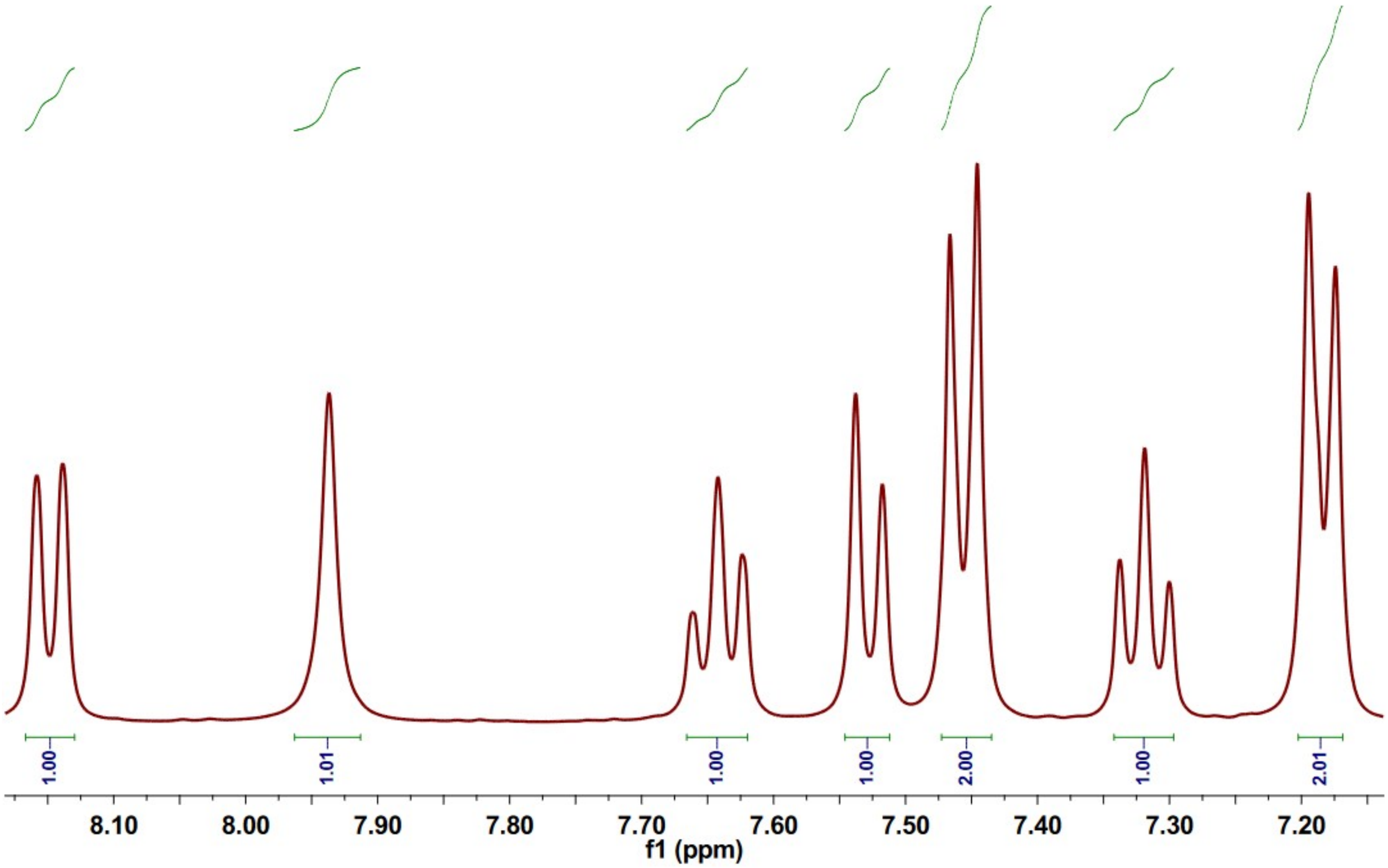

Figure S79: Expanded aromatic region of ^{1}H NMR spectrum (400 MHz, $CDCl_3$) of compound **8t**

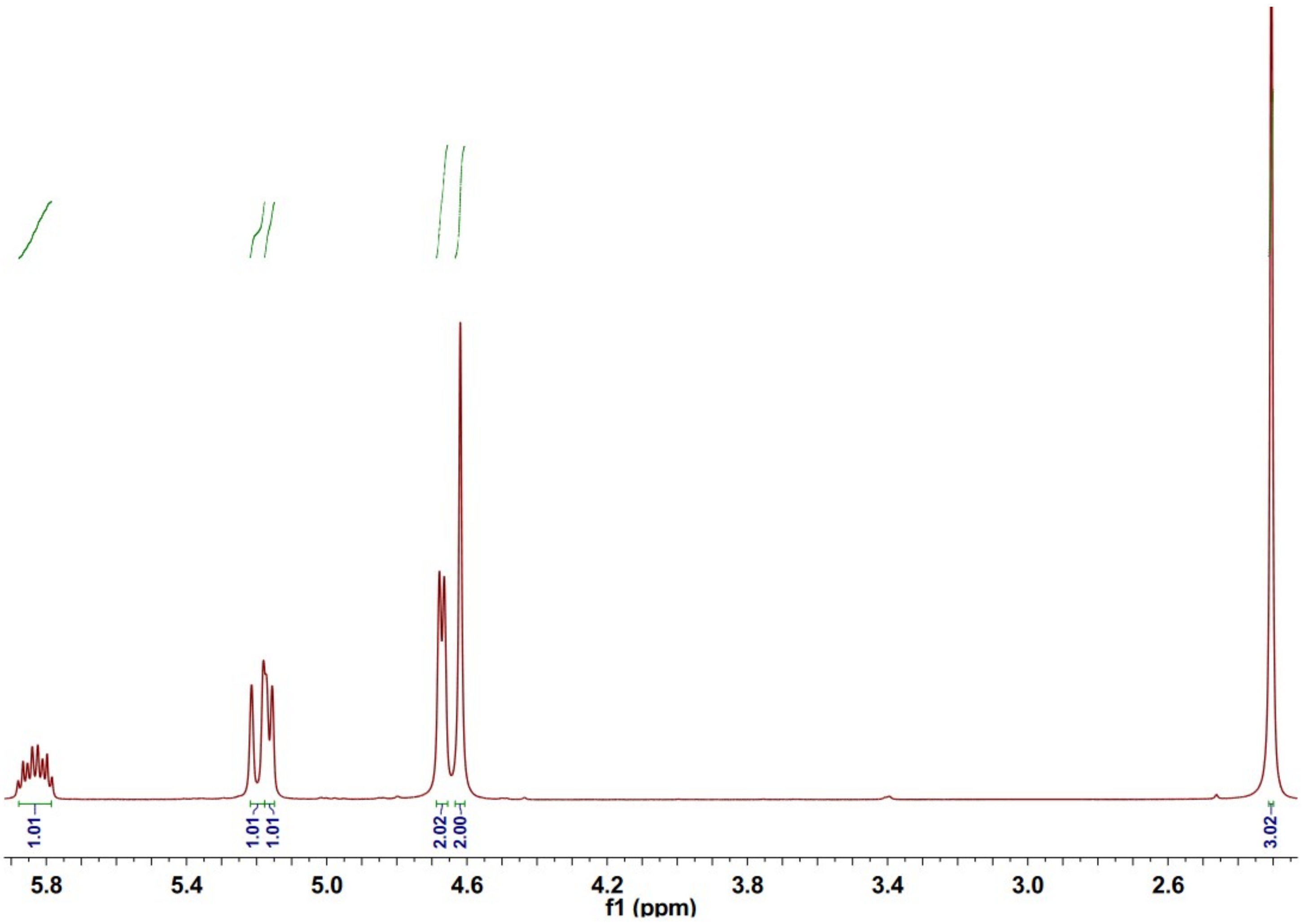

Figure S80: Expanded aliphatic and olefinic region regions of ^{1}H NMR spectrum (400 MHz, $CDCl_3$) of compound **8t**

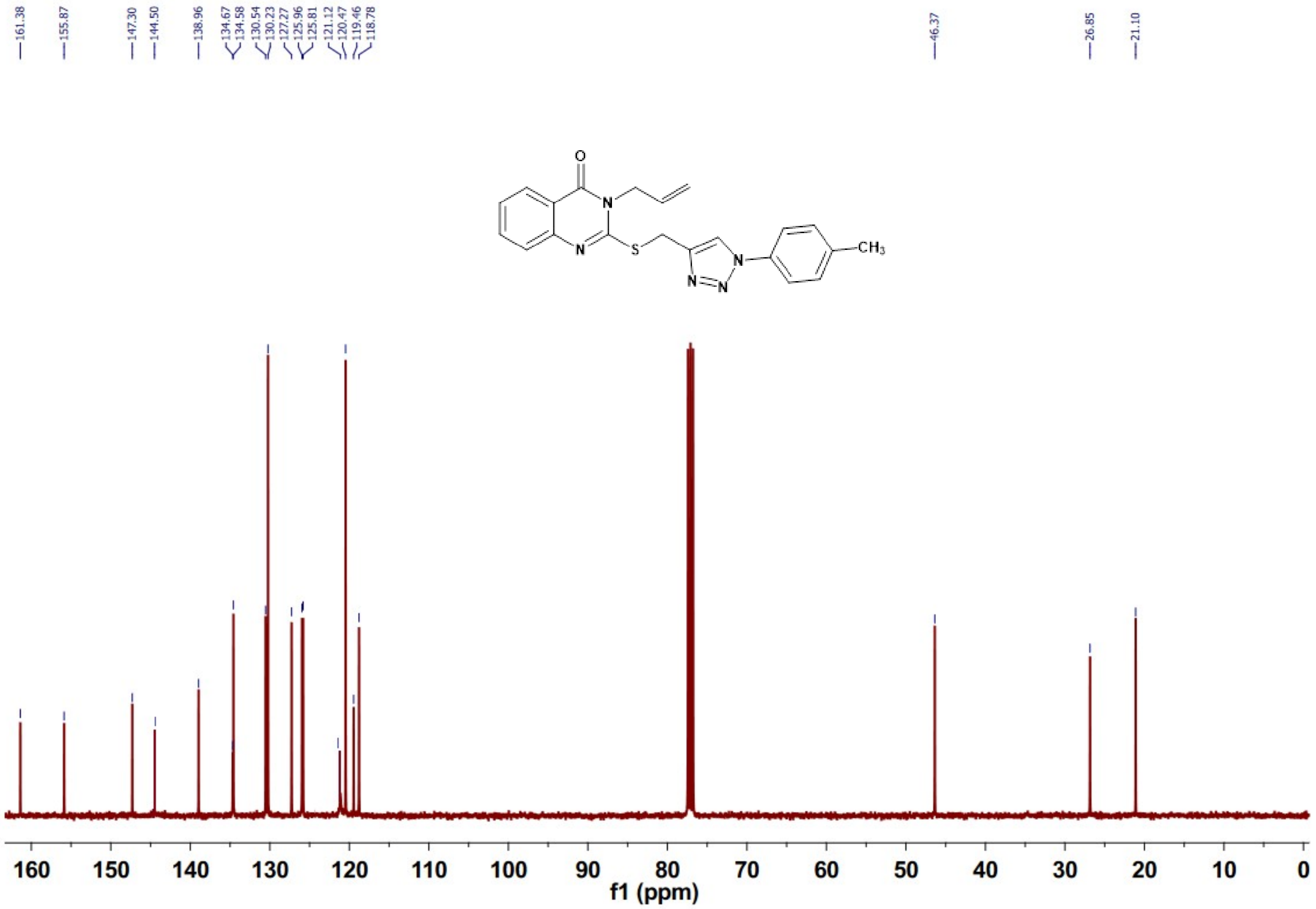

Figure S81: ^{13}C NMR spectrum (100 MHz, $CDCl_3$) of compound **8t**

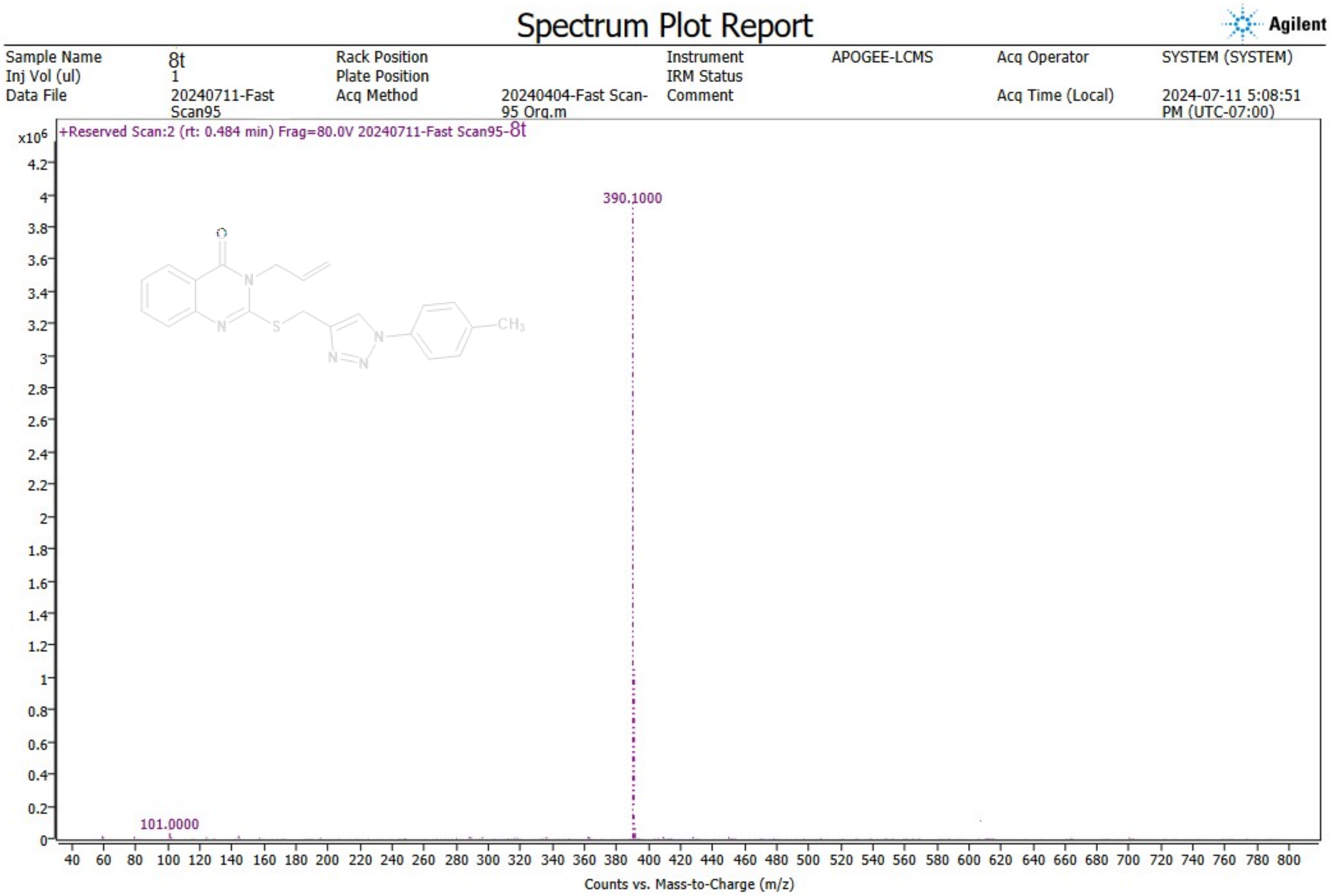

Figure S82: ESI-Mass spectrum of compound **8t**

4. EXPERIMENTAL

4.1. Chemistry

General details

All the reactions progress were monitored with TLC (thin-layer chromatography) on Merck alumina-backed TLC plates and visualized under UV light. All spectral data were measured in DMSO-d_6 on a Bruker AV-300 spectrometer (300 MHz for ^{1}H and 75 MHz for ^{13}C) in the Micro Analytical Center, Cairo University, Egypt. Chemical shifts are expressed in δ (ppm) versus internal Tetramethylsilane (TMS) = 0 ppm for ^{1}H and ^{13}C. Also, the chemical shifts (δ) are reported in parts per million (pm) relative to Tetramethylsilane (TMS) as internal standard, and the coupling constants (*J*) are reported in Hertz (Hz). Splitting patterns are denoted as follows: singlet (s), doublet (d), multiplet (m), triplet (t), quartet (q) and doublet of doublets (dd). Melting points (mp) were determined with a Stuart melting point instrument and are expressed in °C. Agilent Infinity 1290 connected to Triple Quad-6460 mass spectrometer was used for LC-MS.

4.2. Biological evaluation

4.2.1. Cytotoxic activity using MTT Assay and evaluation of IC_{50}

4.2.1.1. MTT assay

MTT assay was carried out to study the effect of compounds on mammary epithelial cells (MCF-10A). The medium in which cells were propagated contained Dulbecco's modified Eagle's medium (DMEM)/ Ham's F-12 medium (1:1) supplemented with epidermal growth factor (20 ng/mL), hydrocortisone (500 ng/mL), insulin (10 µg/mL), 2 mM glutamine and 10% foetal calf serum. After every 2-3 days, the cells were passaged using trypsin ethylenediamine tetra acetic acid (EDTA). The cells were seeded at a density of 10^4 cells mL^{-1} in flat-bottomed culture plates containing 96 wells each. After 24 h, medium was removed from the plates and the compounds in (in 0.1% DMSO) were added (in 200 µL medium to yield a final concentration of 0.1% v/v) to the wells of plates. A single compound was designated with four wells followed by incubation of plates for 96h at 37°C. After incubation, medium was removed completely from the plates followed by addition of MTT (0.4 mg/mL in medium) to each well and subsequent incubation of plates for 3h. MTT (along with the medium) was removed and DMSO (150µL) was added to each well of the culture plates, followed by vortexing and subsequent measurement of absorbance (at 540 nm) using microplate reader. The data are shown as percentage inhibition of proliferation in comparison with controls containing 0.1% DMSO.

4.2.1.2. Assay for antiproliferative effect

To explore the antiproliferative potential of compounds MTT assay was performed according to previously reported procedure using different cell lines to explore the antiproliferative potential of compounds propidium iodide fluorescence assay was performed using different cell lines. To calculate the total nuclear DNA, a fluorescent dye (propidium iodide, PI) is used which can attach to the DNA, thus offering a quick and precise technique. PI cannot pass through the cell membrane and its signal intensity can be considered as directly proportional to quantity of cellular DNA. Cells whose cell membranes are damaged or have changed permeability are counted as dead ones. The assay was performed by seeding the cells of different cell lines at a density of 3000-7500 cells/well (in 200µl medium) in culture plates followed by incubation for 24h at 37 °C in humidified 5% CO_2/95% air atmospheric conditions. The medium was removed; the compounds were added to the plates at 10 µM concentrations (in 0.1% DMSO) in triplicates, followed by incubation for 48 h. DMSO (0.1%) was used as control. After incubation, medium was removed followed by the addition of PI (25 µl, 50µg/mL in water/medium) to each well of the plates. At -80 °C, the plates were allowed to freeze for 24 h, followed by thawing at 25°C. A fluorometer (Polar-Star BMG Tech) was used to record the readings at excitation and emission wavelengths of 530 and 620 nm for each well. The percentage cytotoxicity of compounds was calculated using the following formula:

$$\% \text{ Cytotoxicity} = \frac{A_C - A_{TC}}{A_C} \times 100$$

Where A_{TC}= Absorbance of treated cells and A_C= Absorbance of control. Erlotinib was used as positive control in the assay.

4.2.1.3. EGFR inhibitory assay

EGFR-TK assay was performed to evaluate the inhibitory potency of the most potent compounds against EGFR. Baculoviral expression vectors including pBlueBacHis2B and pFASTBacHTc were used separately to clone 1.6 kb cDNA coding for EGFR cytoplasmic domain (EGFR-CD, amino acids 645–1186). 5′ upstream to the EGFR sequence comprised a sequence that encoded $(His)_6$. Sf-9 cells were infected for 72h for protein expression. The pellets of Sf-9 cells were solubilized in a buffer containing sodium vanadate (100 μM), aprotinin (10 μg/mL), triton (1%), HEPES buffer (50mM), ammonium molybdate (10 μM), benzamidine HCl (16 μg/mL), NaCl (10 mM), leupeptin (10 μg/mL) and pepstatin (10 μg/mL) at 0°C for 20 min at pH 7.4, followed by centrifugation for 20 min. To eliminate the non-specifically bound material, a Ni-NTA super flow packed column was used to pass through and wash the crude extract supernatant first with 10 mM and then with 100 mM imidazole. Histidine-linked proteins were first eluted with 250 and then with 500 mM imidazole subsequent to dialysis against NaCl (50 mM), HEPES (20 mM), glycerol (10%) and 1 μg/mL each of aprotinin, leupeptin and pepstatin for 120 min. The purification was performed either at 4 °C or on ice. To record autophosphorylation level, EGFR kinase assay was carried out on the basis of DELFIA/Time-Resolved Fluorometry. The compounds were first dissolved in DMSO absolute, subsequent to dilution to appropriate concentration using HEPES (25 mM) at pH 7.4. Each compound (10 μL) was incubated with recombinant enzyme (10 μL, 5 ng for EGFR, 1:80 dilution in 100 mM HEPES) for 10 min at 25°C, subsequent to the addition of 5X buffer (10 μL, containing 2 mM $MnCl_2$, 100 μM Na_3VO_4, 20 mM HEPES and 1 mM DTT) and ATP-$MgCl_2$ (20 μL, containing 0.1 mM ATP and 50 mM $MgCl_2$) and incubation for 1h. The negative and positive controls were included in each plate by the incubation of enzyme either with or without ATP-$MgCl_2$. The liquid was removed after incubation and the plates were washed thrice using wash buffer. Europium-tagged antiphosphotyrosine antibody (75 μL, 400 ng) was added to

each well followed by incubation of 1h and then washing of the plates using buffer. The enhancement solution was added to each well and the signal was recorded at excitation and emission wavelengths of 340 at 615 nm. The autophosphorylation percentage inhibition by compounds was calculated using the following equation:

$$100\% - [(negative\ control)/(positive\ control) - (negative\ control)]$$

Using the curves of percentage inhibition of eight concentrations of each compound, IC_{50} was calculated. Majority of signals detected by antiphosphotyrosine antibody were from EGFR because the enzyme preparation contained low impurities.

4.2.1.4. BRAF kinase assay

V^{600E} mutant BRAF kinase assay was performed to investigate the activity of tested compounds against BRAF. Mouse full-length GST-tagged $BRAF^{V600E}$ (7.5 ng, Invitrogen, PV3849) was pre-incubated with drug (1 µL) and assay dilution buffer (4 µL) for 60 min at 25°C. In assay dilution buffer, a solution (5 µL) containing $MgCl_2$ (30 mM), ATP (200 µM), recombinant human full length (200 ng) and *N*-terminal His-tagged MEK1 (Invitrogen) was added to start the assay, subsequent to incubation for 25 min at 25°C. The assay was stopped using 5X protein denaturing buffer (LDS) solution (5 µL). To further denature the protein, heat (70° C) was applied for 5 min. 4-12% precast NuPage gel plates (Invitrogen) were used to carry out electrophoresis (at 200 V). 10 µL of each reaction was loaded into the precast plates and electrophoresis was allowed to proceed. After completion of electrophoresis, the front part of the precast gel plate (holding hot ATP) was cut and afterwards cast-off. The dried gel was developed using a phosphor screen. A reaction without active enzyme was used as negative control while that containing no inhibitor served as positive control. To study the effect of compounds on cell-based pERK1/2 activity in

cancer cells, commercially available ELISA kits (Invitrogen) were used according to manufacturer's instructions.

4.2.1.5. Caspase-3 activation assay

Allow all reagents to reach room temperature before use. Gently mix all liquid reagents prior to use. Determine the number of 8-well strips needed for the assay. Insert these in the frame(s) for current use. Add 100 µl of the *Standard Diluent Buffer* to the zero standard wells. Well(s) reserved for chromogen blank should be left empty. Add 100 µl of standards and controls or diluted samples to the appropriate microtiter wells. The sample dilution chosen should be optimized for each experimental system. Tap gently on side of plate to mix. Cover wells with *plate cover* and incubate for 2 hours at room temperature. Thoroughly aspirate or decant solution from wells and discard the liquid, Wash wells 4 times. Pipette 100 µl of *Caspase-3 (Active) Detection Antibod*y solution into each well except the chromogen blank(s). Tap gently on the side of the plate to mix. Cover plate with *plate cover* and incubate for 1 hour at room temperature. Thoroughly aspirate or decant solution from wells and discard the liquid, Wash wells 4 times. Add 100 µl Anti-Rabbit IgG HRP Working Solution to each well except the chromogen blank(s). Prepare the working dilution as described in Preparing IgG HRP. Cover wells with the *plate cover* and incubate for 30 minutes at room temperature. Thoroughly aspirate or decant solution from wells and discard the liquid. Wash wells 4 times. Add 100 µl of *Stabilized Chromogen* to each well. The liquid in the wells will begin to turn blue. Incubate for 30 minutes at room temperature and in the dark. The incubation time for chromogen substrate is often determined by the microtiter plate reader used. Many plate readers have the capacity to record a maximum optical density (O.D.) of 2.0. The O.D. values should be

monitored, and the substrate reaction stopped before the O.D. of the positive wells exceeds the limits of the instrument. The O.D. values at 450 nm can only be read after the *Stop Solution* has been added to each well. If using a reader that records only to 2.0 O.D., stopping the assay after 20 to 25 minutes is suggested. Add 100 µl of *Stop Solution* to each well. Tap side of plate gently to mix. The solution in the wells should change from blue to yellow. Read the absorbance of each well at 450 nm having blanked the plate reader against a chromogen blank composed of 100 µl each of *Stabilized Chromogen* and *Stop Solution*. Read the plate within 2 hours after adding the *Stop Solution*. Use a curve fitting software to generate the standard curve. A four-parameter algorithm provides the best standard curve fit. Read the concentrations for unknown samples and controls from the standard curve. Multiply value(s) obtained for sample(s) by the appropriate dilution factor to correct for the dilution in step 3. Samples producing signals greater than that of the highest standard should be diluted in *Standard Diluent Buffer* and reanalyzed.

4.2.1.6. Caspase-8 activation assay

Cells were obtained from American Type Culture Collection, cells were grown in RPMI 1640 containing 10% fetal bovine serum at 37°C, stimulated with the compounds to be tested for caspase8, and lysed with Cell Extraction Buffer. This lysate was diluted in Standard Diluent Buffer over the range of the assay and measured for human active caspase-8 content. (*Cells are Plated in a density of 1.2 – 1.8 × 10,000 cells/well in a volume of 100µl complete growth medium + 100 ul of the tested compound per well in a 96-well plate for 24 hours before the enzyme assay for Tubulin.*). The absorbance of each microwell was read on a spectro-photometer at 450 nm. A standard curve is prepared from 7human Caspase-8 standard dilutions and human Caspase-8 concentration determined.

4.2.1.7. Bax activation assay

Bring all reagents, except the human Bax-α Standard, to room temperature for at least 30 minutes prior to opening. The human Bax-α Standard solution should not be left at room temperature for more than 10 minutes. All standards, controls and samples should be run in duplicate. Refer to the Assay Layout Sheet to determine the number of wells to be used and put any remaining wells with the desiccant back into the pouch and seal the ziploc. Store unused wells at 4 °C. Pipet 100 µL of Assay Buffer into the S0 (0 pg/mL standard) wells. Pipet 100 µL of Standards #1 through #6 into the appropriate wells. Pipet 100 µL of the Samples into the appropriate wells. Tap the plate gently to mix the contents. Seal the plate and incubate at room temperature on a plate shaker for 1 hour at ~500 rpm. Empty the contents of the wells and wash by adding 400 µL of wash solution to every well. Repeat the wash 4 more times for a total of 5 washes. After the final wash, empty or aspirate the wells and firmly tap the plate on a lint free paper towel to remove any remaining wash buffer. Pipet 100 µL of yellow Antibody into each well, except the Blank. Seal the plate and incubate at room temperature on a plate shaker for 1 hour at ~500 rpm. Empty the contents of the wells and wash by adding 400 µL of wash solution to every well. Repeat the wash 4 more times for a total of **5** washes. After the final wash, empty or aspirate the wells and firmly tap the plate on a lint free paper towel to remove any remaining wash buffer. Add 100 µL of blue Conjugate to each well, except the Blank. Seal the plate and incubate at room temperature on a plate shaker for 30 minutes at ~500 rpm. Empty the contents of the wells and wash by adding 400 µL of wash solution to every well. Repeat the wash 4 more times for a total of **5 washes**. After the final wash, empty or aspirate the wells and firmly tap the plate on a lint free paper towel to remove any remaining wash buffer. Pipet 100 µL of Substrate Solution into each well. Incubate for 30 minutes at room temperature on a plate shaker at ~500 rpm. Pipet 100 µL Stop Solution to each well. Blank the plate reader against the Blank wells, read the optical density at 450 nm. Calculate the average net Optical Density

(OD) bound for each standard and sample by subtracting the average Blank OD from the average OD for each standard and sample. Using linear graph paper, plot the Average Net OD for each standard versus Bax concentration in each standard. Approximate a straight line through the points. The concentration of Bax in the unknowns can be determined by interpolation.

4.2.1.8. Bcl-2inhibition assay

Mix all reagents thoroughly without foaming before use. Wash the microwells twice with approximately 300 µL Wash Buffer per well with thorough aspiration of microwell contents between washes. Take caution not to scratch the surface of the microwells. After the last wash, empty the wells and tap microwell strips on absorbent pad or paper towel to remove excess Wash Buffer. Use the microwell strips immediately after washing or place upside down on a wet absorbent paper for not longer than 15 minutes. Do not allow wells to dry. Add 100 µL of Sample Diluent in duplicate to all standard wells and to the blank wells. Prepare standard (1:2 dilution) in duplicate ranging from 32 ng/mL to 0.5 ng/mL. Add 100 µL of Sample Diluent, in duplicate, to the blank wells. Add 80 µL of Sample Diluent, in duplicate, to the sample wells. Add 20 µL of each Sample, in duplicate, to the designated wells. Add 50 µL of diluted biotin-conjugate to all wells, including the blank wells. Cover with a plate cover and incubate at room temperature, on a microplate shaker at 100 rpm if available, for 2 hours. Remove plate cover and empty the wells. Wash microwell strips 3 times as described in step 2. Add 100 µL of diluted Streptavidin-HRP to all wells, including the blank wells. Cover with a plate cover and incubate at room temperature, on a microplate shaker at 100 rpm if available, for 1 hour. Remove plate cover and empty the wells. Wash microwell strips 3 times as described in step 2. Proceed to the next step. Pipette 100 µl of mixed TMB Substrate Solution to all wells, including the blanks. Incubate the microwell strips at room temperature (18° to 25°C) for about 15 minutes, if available on a rotator set at 100 rpm.

Avoid direct exposure to intense light. The point, at which the substrate reaction is stopped, is often determined by the ELISA reader. Many ELISA readers record absorbance only up to 2.0 O.D. Therefore, the color development within individual microwells must be watched by the person running the assay and the substrate reaction stopped before positive wells are no longer properly detectable. Stop the enzyme reaction by quickly pipetting 100 μL of Stop Solution into each well, including the blank wells. It is important that the Stop Solution is spread quickly and uniformly throughout the microwells to completely inactivate the enzyme. Results must be read immediately after the Stop Solution is added or within one hour if the microwell strips are stored at 2 - 8°C in the dark. Read absorbance of each microwell on a spectrophotometer using 450 nm as the primary wavelength.

4.2.1.9. Cell apoptosis assay

Apoptosis was determined by flow cytometry based on the Annexin-V-fluoresce in isothiocyanate (FITC) and propidium iodide (PI) staining kit (BD Pharmingen, San Diego, USA) [25, 26]. Apoptosis was determined by flow cytometry based on the Annexin-V-fluoresce in isothiocyanate (FITC) and propidium iodide (PI) staining kit (BD Pharmingen, San Diego, USA). Apoptotic cells were defined as Annexin-V-positive. Cells were grown to approximately ~70% confluence and exposed to different concentrations of compounds (0, 2, 4, 6 and8 μmol/L) for 24 h. Treated cells were trypsinzed, washed twice with PBS and transferred into micro centrifuge tubes for centrifugation at1000 rpm for 5 min at room temperature, then resuspended in binding buffer, 5 μL of FITC and PI were added to Eppendorf tube, cells were vortexed, incubated for 15 min at room temperature in dark. Subsequently, cells were analyzed by flow cytometry (Becton Dickinson, Franklin Lakes, and USA).

4.3. Molecular Docking

Molecular modeling and visualization processes were performed within the active sites of epidermal growth factor receptor (EGFR) and mutant B-Raf protein ($BRAF^{V600E}$ using Molecular Operating Environment (MOE®) software (Chemical Computing Group, Montreal, QC, Canada). The co-crystal structures were retrieved from the RCSB Protein Data Bank (PDB ID: 1M17 and 4RZV; respectively). First, the test compounds were prepared with the standard protocol designated in MOE software. The energy of the docked compounds was minimized using MMF94FX forcefield with gradient RMS of 0.0001kcal/mol, and then the protein structure was prepared by using the MOE LigX protocol. To validate the docking study at the binding sites, the native ligands were re-docked into the binding site using the same set of parameters as described above. The RMSD values of the best-docked poses were 1.28 Å for Erlotinib in 1M17 and 0.62 Å for Vemurafenib in 4RZV active sites, thus validating the docking experiment. Test compounds **8f**, **8g**, **8h**, **8j**, and **8l** were then docked into the binding sites using the Alpha triangle placement method. Refinement was carried out using Forcefield and scored using the Affinity dG scoring system (S; Kcal/mol). The resulting docking poses were visually inspected, and the pose of the lowest binding free energy value was considered for assessment of binding interactions.

4.4. Statistical analysis

Computerized Prism 5 program was used to statistically analyzed data using one-way ANOVA test followed by Tukey's as post ANOVA for multiple comparison at $P \leq .05$. Data were presented as mean ± SEM.
